# Reactions of a Four‐Membered Borete with Carbon, Silicon, and Gallium Donor Ligands: Fused and Spiro‐Type Boracycles

**DOI:** 10.1002/chem.202200673

**Published:** 2022-04-21

**Authors:** Zeynep Güven, Lars Denker, Hadi Dolati, Daniela Wullschläger, Bartosz Trzaskowski, René Frank

**Affiliations:** ^1^ Department of Inorganic and Analytical Chemistry Technische Universität Braunschweig Hagenring 30 38106 Braunschweig Germany; ^2^ Centre of New Technologies University of Warsaw Banacha 2 C 02-097 Warszawa Poland

**Keywords:** donor-acceptor cyclobutanes, inorganic polycycles, inorganic spirocycles, gallium carbenoids, N-heterocyclic carbenes, ring expansion reactions, silicon carbenoids

## Abstract

Donor‐acceptor cyclopropanes or cyclobutanes are dipolar reagents, which are widely used in the synthesis of complex organic (hetero)cycles in ring expansion reactions. Applying this concept to boron containing heterocycles, the four‐membered borete *cyclo*‐*i*Pr_2_N‐BC_10_H_6_ reacted with the carbon donor ligands 2,6‐xylylisonitrile and the carbene IMes :C(NMesCH)_2_ with ring expansion and ring fusion, respectively. In particular, the tetracyclic structure formed with IMes displays zwitterionic character and absorption in the visible region. In contrast to the carbene IMes, the heavier carbenoids :Si(NDippCH)_2_ and :Ga(AmIm) with a two‐coordinate donor atom afford *spiro*‐type bicyclic compounds, which display four‐coordinate geometry at silicon or gallium. (TD‐)DFT calculations provide deeper insight into the mechanism of formation and the absorption properties of these new compounds.

## Graphical abstract

**A four‐membered** naphthalene‐supported borete is investigated as a masked 1,4‐dipolar donor‐acceptor cyclobutane. Ring expansion reactions confirm such type of reactivity and afford complex cyclic structures including fused and *spiro*‐type polycyclics.

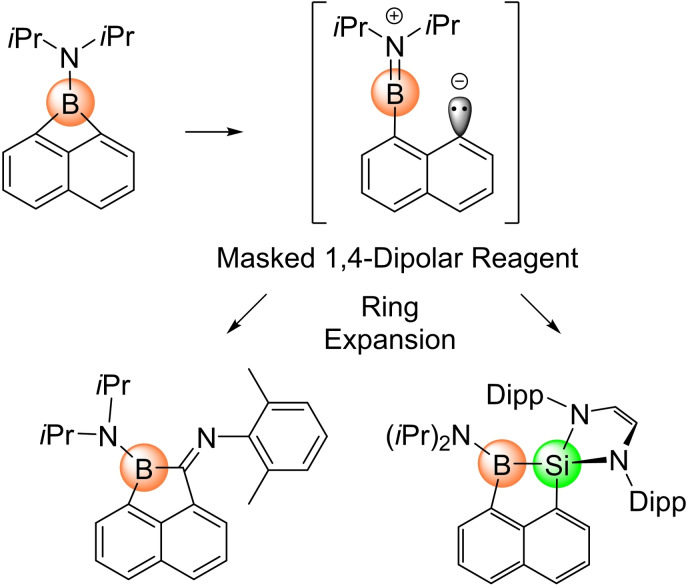

## Introduction

**Scheme 1:**
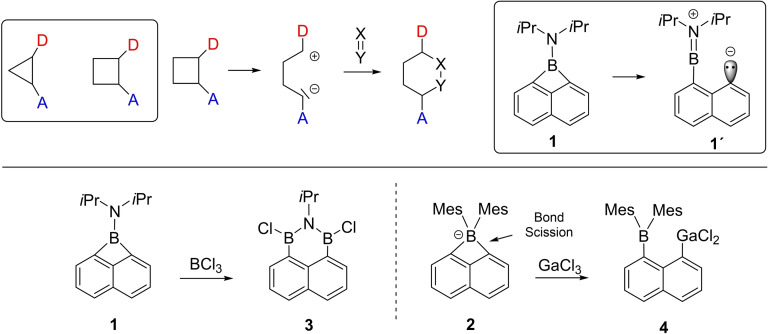
Donor‐acceptor‐substituted cyclopropanes and cyclobutanes. D (donor) or A (acceptor) denote substituents of +M‐ or ‐M‐effect, respectively. The 1,8‐substituted naphthalene platform stabilises four‐membered borete **1** or boretate **2**, which reacted exclusively with electrophiles in the past. Counter cations for **2** were omitted for clarity. Mes=2,4,6‐trimethylphenyl.

The generation of complex cyclic structures is one of the key areas in synthetic chemistry and includes the formation of *spiro*‐type or fused ring systems. Among many other methods, ring opening reactions of three‐ or four‐membered (hetero)cycles have been widely exploited in the past.^[1]^ In particular, in organic chemistry this concept was applied for donor‐acceptor cyclopropanes or cyclobutanes, which can be considered as 1,3 or 1,4‐dipolar reagents based on their (hypothetically) opened form to undergo ring expansion, Scheme 1.^[2]^ This concept has never been considered in the context of boron‐containing heterocycles, which may be partly due to the limited number of boracycles. In the case of four‐membered boron containing rings, the 1,8‐disubsitituted naphthalene platform has been employed in the synthesis of the four‐membered borete **1** or boretate **2**.^[3]^ The *peri*‐substitution pattern in the naphthalene scaffold essentially stabilises the four‐membered ring architecture with a bridged single boron centre. Interestingly, besides **1** and **2** only a few other examples of boretanes or boretes have been reported,^[4]^ and a comprehensive understanding of their reactivity is currently missing. The synthetic interest in four‐membered (hetero)cycles can be traced back to their significant inherent ring strain, which mostly originates from contributions of Baeyer strain.^[5]^ In line with these facts, ring scission reactions are facile for **1** and **2**, but such behaviour is currently documented only for electrophilic reagents. Examples include the reaction gallium(III)‐chloride with **2** under ring opening towards **4**. Ring expansion reactions to afford extended cycles have only been reported for one example, i. e. the reaction of **1** with boron trichloride to give the six‐membered diboracycle **3**.^[1a]^

We hypothesise that inherent donor‐acceptor reactivity may be operative in **1** based on the formal formulation of hypothetical **1'** with both electrophilic behaviour at boron and nucleophilic reactivity at the carbanionic centre. With this in mind and in consideration of the fact that the four‐membered boracycles **1** and **2** were exclusively subjected to electrophiles in the past, we set out to investigate the reactivity of borete **1** with reagents, which typically display donor acceptor reactivity, for example in transition metal complexes. Representative examples include carbon monoxide, isonitriles, N‐heterocyclic carbenes, and carbenoid reagents (silylenes, gallylenes). The availability of both donor and acceptor orbitals in these species lend themselves to be excellent reagents in ring expansion reagents involving the donor acceptor heterocycle concept.

## Results and Discussion

**Scheme 2:**
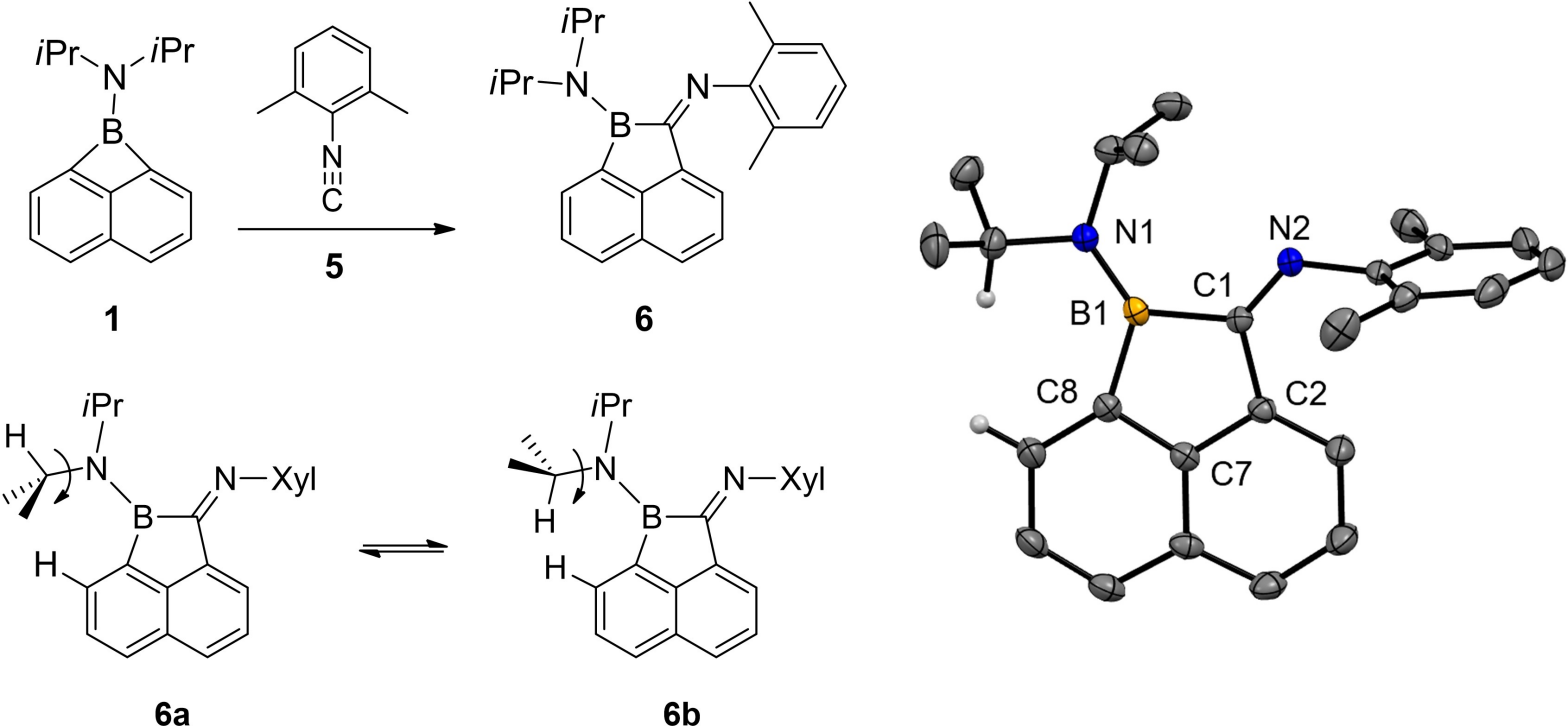
Conditions: toluene, rt, 5 min. Molecular structure of compound **6**. Thermal ellipsoids are presented on the 50 % level of probability. Hydrogen atoms except for relevant C−H moieties are omitted for clarity. Selected bond lengths (Å) and bond angles (°): N1‐B1 1.394(2), N2‐C1 1.280(2) N2‐C1 1.280(2), C2‐C7 1.422(2), C7‐C8 1.432(2), C8‐B1 1.591(2), C2‐C1‐B1 106.24(13), C7‐C2‐C1 108.71(13), C7‐C8‐B1 106.19(13), C8‐B1‐C1 102.88(13). Xyl=2,6‐dimethylphenyl.

While no reaction with carbon monoxide (pressure up to 10 bar) was observed, borete **1** rapidly reacted with 2,6‐dimethylphenyl isonitrile **5**, Scheme 2. The resulting orange crystalline compound **6** may be considered as an insertion product of **5** into the B−C bond in **1** with concomitant expansion to five‐membered cyclic boraimine **6**. The insertion of isonitriles into B−C bonds has occasionally been reported for cases in which the boron atom was highly Lewis acidic and π‐donor substituents were absent.^[6]^ Thus, the facile reaction of borete **1** with isonitrile **5** is a result of the significant Baeyer strain and underpins the analogy in view of the reactivity of **1** as the donor‐acceptor boracycle.

**Figure 1:**
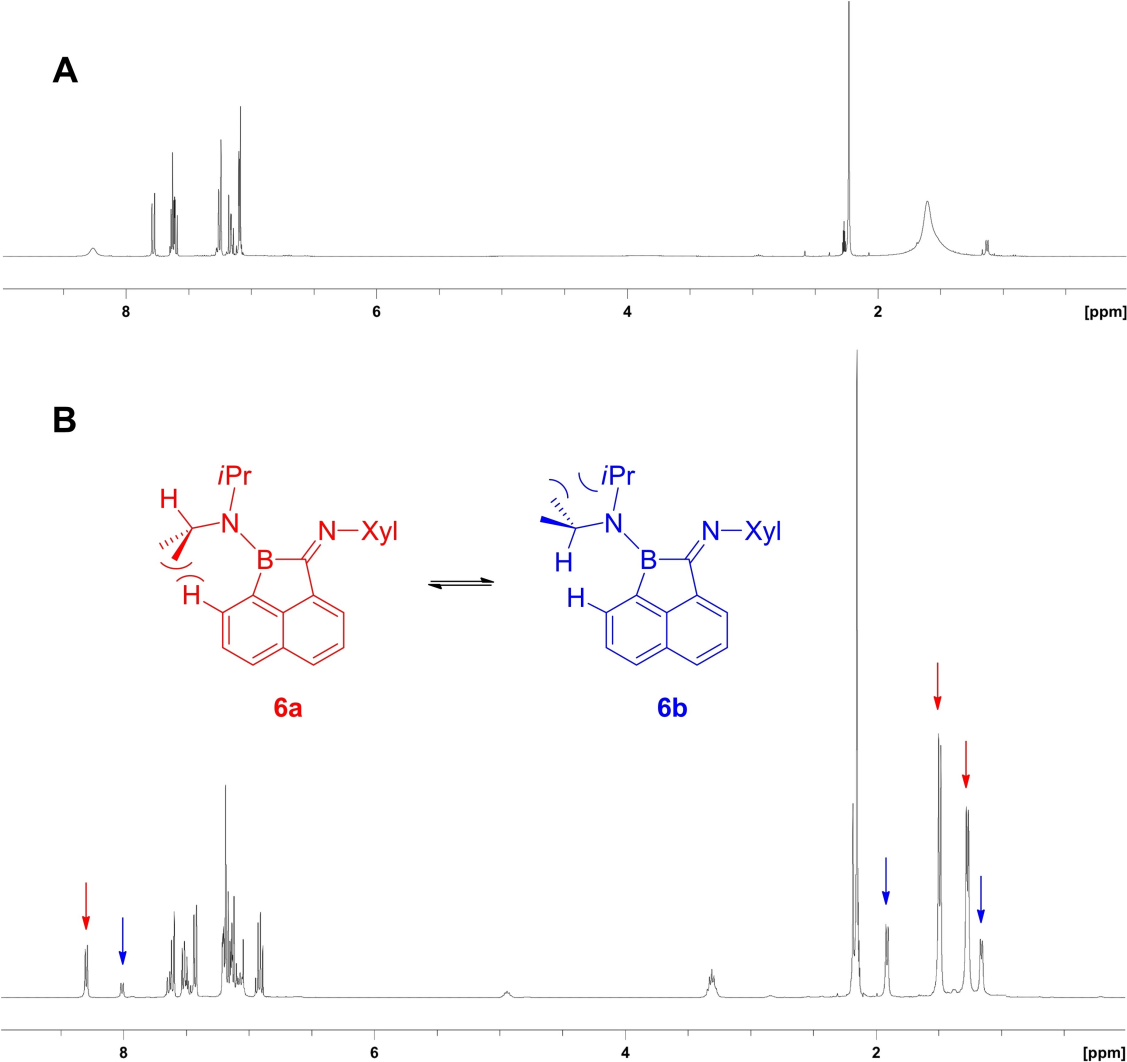
^1^H NMR spectra of compound **6** in toluene‐D_8_. A) 25° C. B) −60 °C. The arrows indicate diagnostic signals of conformers **6 a** or **6 b**. The complete assignment can be found in the Supporting Information. Xyl=2,6‐di‐methylphenyl.

The identity of **6** was unambiguously confirmed by X‐ray crystallo‐graphic analysis. The nitrogen atom N1 at the diisopropylamino group and the boron centre B1 both appear in a trigonal planar geometry, and the lone pair (at N1) is found in π‐conjugation coplanar to the vacant p‐orbital (at B1). The ^11^B{^1^H} NMR spectrum of compound **6** displayed a broad singlet at 37.4 ppm (s, ω_1/2_=191 Hz) compared to the narrower signal of starting material **1** at 35.8 ppm (ω_1/2_=408 Hz). The ^1^H NMR spectrum of **6** showed broad signals at ambient temperature that indicate dynamic behaviour. Variable temperature NMR experiments (vt ^1^H NMR spectroscopy, rt to −80 °C, toluene‐D_8_) gave rise to significant line narrowing and led to the detection of two interconvertible species **6 a** and **6 b**, Figure 1. Further 2D NMR experiments (H,H‐COSY, ROESY) at −60 °C facilitated the assignment of distinct signals of species **6 a** and **6 b**. In particular, the ROESY spectra for **6 b** revealed cross peaks of the internal proton in Me_2_C*H* with the adjacent naphthalene proton. The conformer **6 b** was crystallographically observed, and both forms **6 a** and **6 b** are distinguishable due to a hindered rotation process of the outward‐oriented *i*Pr group around the N−C bond axis. The rotation barrier of **6** can be traced back to the steric repulsion of the methyl or hydrogen *i*Pr substituents with the adjacent proton of the naphthalene scaffold and the second isopropyl substituent, Scheme 2. We did not detect any involvement of the imine moiety, which might display conceivable E/Z double bond isomerism at the C=N‐Xyl moiety. The characteristic methyl groups in the 2,6‐xylyl moiety gave a sharp singlet over the whole temperature range, which excludes a contribution to the dynamic process observed. The thermodynamic van't Hoff plot ln K vs. 1/T (equilibrium constant K with consideration of the two‐fold population for **6 b**, see Supporting Information) reveals a linear correlation. The enthalpy and entropy at standard conditions for the conversion **6 a**→**6 b** were found to be ▵H°=(3.8±1.3) kJ⋅mol^−1^ and ▵S°=(3.2±1.5) J⋅K^−1^⋅mol^−1^, which indicates the energetic preference of **6 a** over **6 b** at low temperature.

**Scheme 3:**
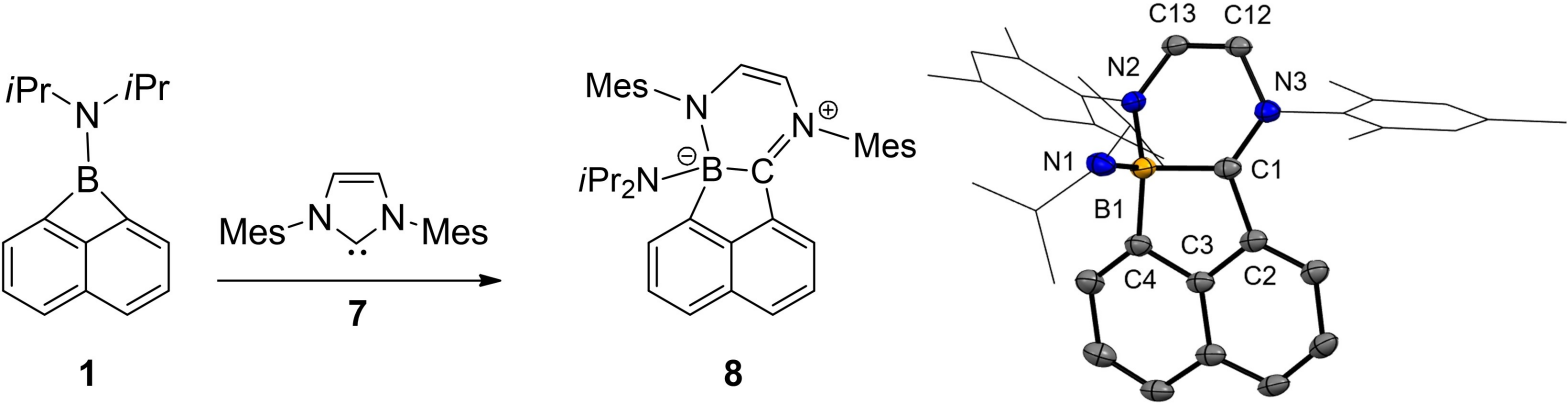
Conditions: toluene, rt, 60 min. Molecular structures of compound **7**. Thermal ellipsoids are illustrated at the 50 % level of probability. Hydrogen atoms are omitted for clarity. Selected bond lengths (Å) and bond angles (°): N1‐B1 1.5328(15), N2‐B1 1.5905(15), C1‐B1 1.6346(17), C4‐B1 1.6360(16), N3‐C1 1.3253(14), N3‐C12 1.4028(14), N2‐C13 1.3527(14), C2‐C1 1.4664(14), C1‐B1 1.6346(17), N3‐C1‐C2 125.22(10), N3‐C1‐B1 123.98(9), C2‐C1‐B1 109.69(9). Mes=2,4,6‐trimethylphenyl.

The N‐heterocyclic carbene **7** (IMes) was found to react with borete **1** in toluene solution, Scheme 3. The reaction reached completion within one hour with the formation of a red precipitate. The latter afforded crystals suitable for X‐ray crystallography, which led to the identification of the molecular structure, compound **8**.

**Figure 2:**
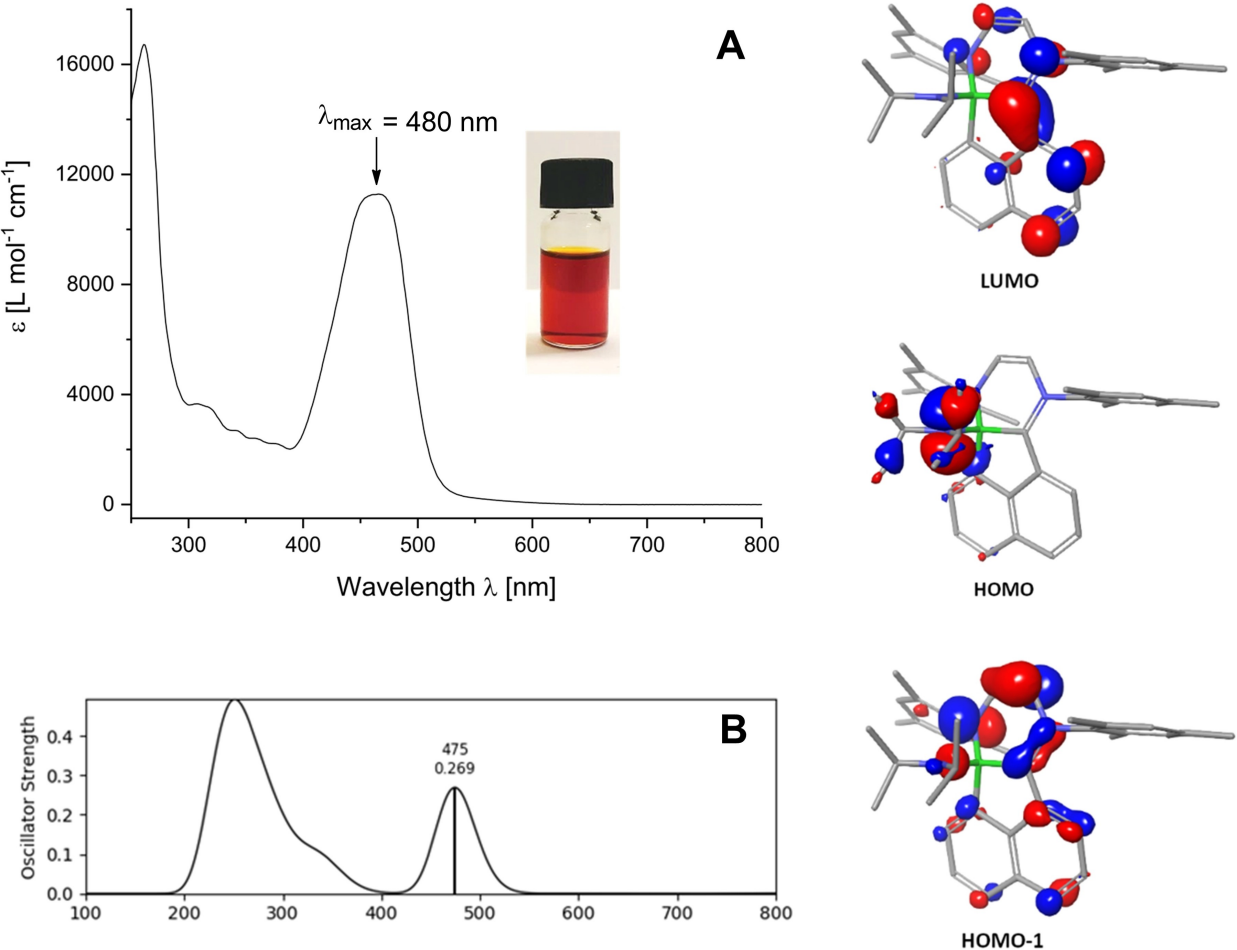
A) Experimental UV/VIS spectrum of 8 in CH_2_Cl_2_. B) Calculated UV/VIS spectrum of **8**. C) Relevant frontier orbitals of **8**.

The formation of **8** can be viewed as a twofold ring expansion reaction involving both the four‐membered borete **1** (C−B bond insertion) and the five‐membered carbene IMes **7** (N−C bond insertion), which concomitantly gives rise to the highly annulated (tetracyclic) ring system. Remarkably compound **8** displays a strong inherent charge separation (zwitterionic nature). The positive formal charge is located at N3 and carbon atom C1 displays trigonal planar geometry [sum of angles 358.9(3)°], while the negative counter charge resides at the four‐coordinate boron atom B1. The iminium type nature of compound **8** is in line with the short N3−C1 bond distance [1.3253(14) Å] due to π‐donation of the lone electron pair located at N3. The four‐coordinate nature of B1 is also corroborated by the narrow ^11^B{^1^H} signal at −1.2 (s, ω_1/2_=85 Hz). In accordance with the intense red colour of compound **8**, the UV/VIS spectrum recorded in dichloromethane displays a strong single absorption band with maximum absorption at λ_exp_=480 nm, Figure 2. For further insight, the molecular orbitals were calculated employing the DFT methods (see Supporting Information). While the canonical HOMO is mainly centred on N1 and represents the nitrogen lone pair, the orbitals HOMO‐1 and LUMO are delocalised over the arene rings. The LUMO exhibits a considerable contribution at the cationic carbon atom C1. The calculated UV/VIS spectrum (TD‐DFT level) is in excellent agreement with the experiment and reproduces the band in the visible region with a calculated maximum λ_calc_=475 nm compared to the experimental value of λ_max_=480 nm. The absorption band can be traced back mainly to electronic excitation from the HOMO‐1 to LUMO level, which corresponds to an S_0_→S_2_ transition.

**Scheme 4:**
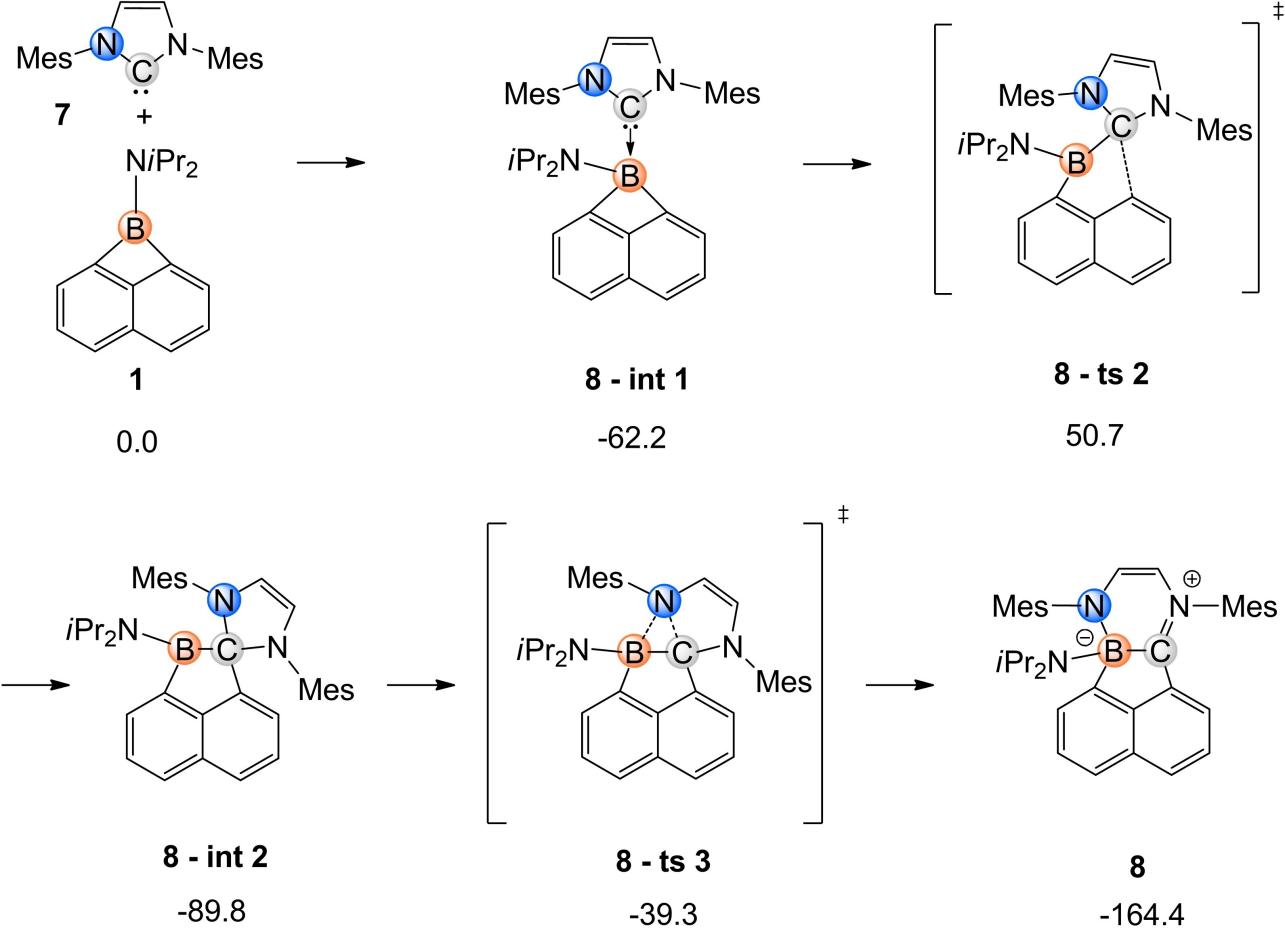
Proposed mechanism for the formation of **8** based on DFT calculations. Values of the standard free enthalpy of intermediates and transition states (▵G^°^ or ▵G^ǂ^) are reported in kJ/mol. Mes=2,4,6‐trimethyl‐phenyl.

Ring expansion reactions in N‐heterocyclic carbenes (NHCs) have been studied in the past, and in their event the insertion of atomic moieties into the C−N bond afforded rings expanded by one vertex atom.[^7^, ^8^] In the formation of compound **8**, a double ring expansion process in borete **1** and NHC **7** gives rise to a fused ring system with the common site C1−B1. DFT calculations suggest a stepwise mechanism (Scheme 4) with the barrier‐free nucleophilic attack of IMes **7** to **1** to form the borete‐NHC‐adduct **8 ‐ int 1**.^[9]^ In the first insertion event the distinct carbon atom in the coordinated carbene opens up the borete ring (C−B bond scission, **8 ‐ ts 2**) with a Gibbs free activation energy ▵G^ǂ^ of 50.7 kJ/mol, which is considered low enough to proceed at an ambient temperature, despite being the rate‐limiting step of the entire reaction. In product **8 ‐ int 2**, the four‐coordinate geometry of the carbene carbon atom is usually considered unfavourable and a second ring insertion of the boron atoms occurs (N−C bond scission, **8 ‐ ts 3**). The fused ring system **8** is formed in a highly exergonic reaction (▵G^°^=−164.7 kJ/mol), which is mostly due to ring strain relaxation of the four‐membered borete, see **8 ‐ int 2**.

**Scheme 5:**
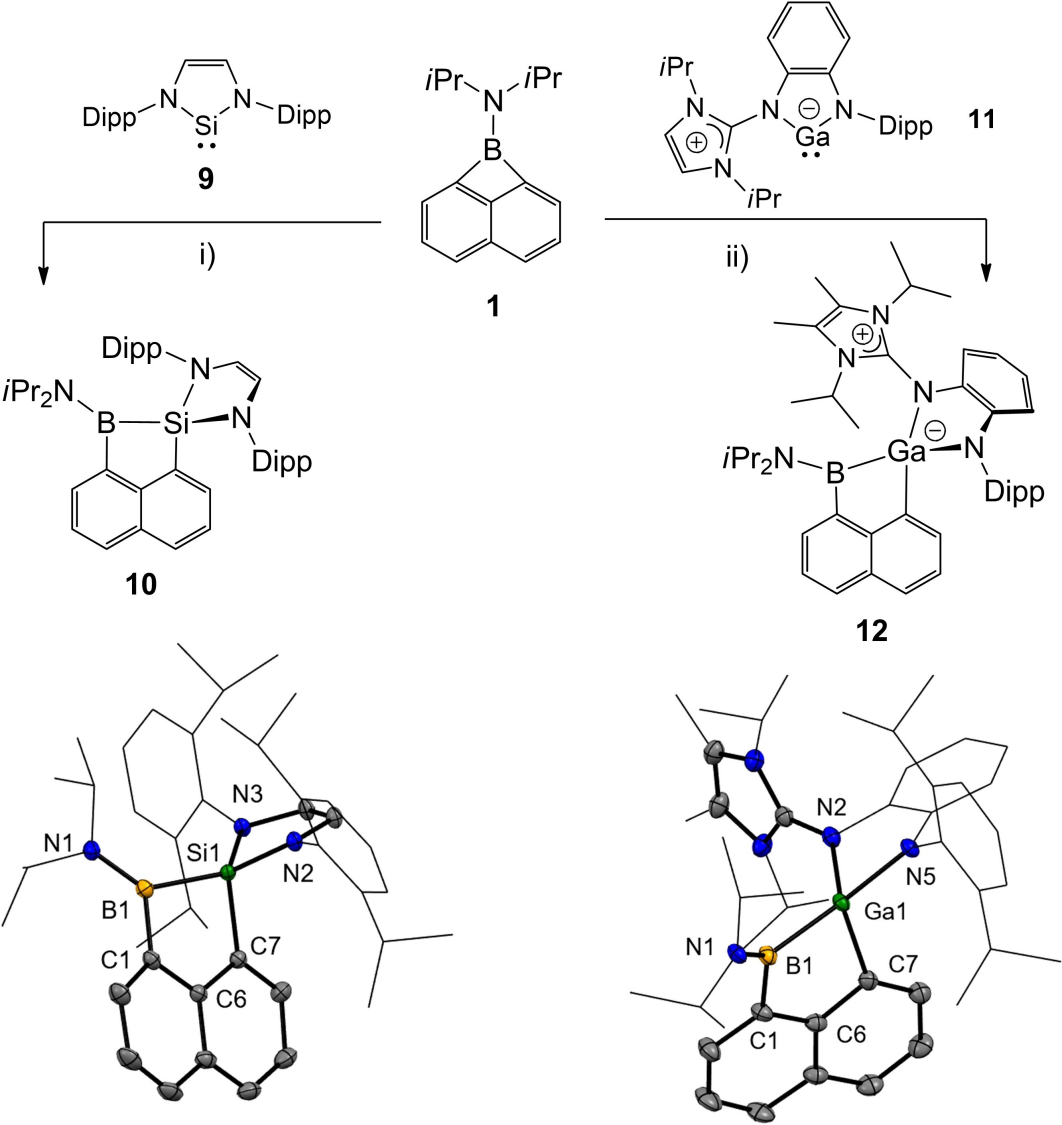
Conditions: i) toluene, 80 °C, overnight. ii) THF, 50 °C, overnight. Molecular structures of compounds **10** and **12**. Thermal ellipsoids are illustrated at the 50 % level of probability. Hydrogen atoms are omitted for clarity. Selected bond lengths (Å) and bond angles (°): For **10**: Si1‐N3 1.7570(7), Si1‐N2 1.7587(7), Si1‐C7 1.8825(8), Si1‐B1 2.0508(9), N1‐B1 1.3972(11), C1‐B1 1.5877(12), N3‐Si1‐N2 91.07(3), N3‐Si1‐C7 114.23(4), N2‐Si1‐C7 109.65(3), N3‐Si1‐B1 129.00(3), N2‐Si1‐B1 123.47(3), C7‐Si1‐B1 90.00(4), C1‐B1 ‐Si1 102.07(5). For **12**: Ga1‐N5 1.9436(17), Ga1‐N2 2.0047(19), Ga1‐C7 2.023(2), Ga1‐B1 2.102(3), N1‐B1 1.400(3), N5‐Ga1‐N2 82.98(8), N5‐Ga1 ‐C7 114.87(9), N2‐Ga1‐C7 103.64(8), N5‐Ga1‐B1 139.16(9), N2‐Ga1‐B1 125.51(9), C7‐Ga1‐B1 88.44(9). Dipp=2,6‐diisopropylphenyl.

The observed reactivity of **1** with IMes **7** as a representative member of N‐heterocyclic carbenes stimulated our curiosity to investigate heavier analogues of carbenoids. In contrast to **7**, the reaction of borete **1** with N‐heterocyclic silylene **9** or *N*‐heterocylic gallylene **11** ^[10]^ did not proceed at ambient temperature, but required gentle heating, Scheme 5. The ^11^B{^1^H} NMR spectrum displays broad signals at 44.5 (for **10**, ω_1/2_=1033 Hz) or 53.5 (for **12**, ω_1/2_=1394 Hz), both downfield shifted and broader compared to starting material **1**. This observation is in line with the lower symmetry and the formation of a B−Si or a B−Ga bond, respectively. Compounds **10** and **12** are formed as insertion products of the two‐coordinate silicon or gallium atom into the C−B bond with concomitant ring expansion of borete **1** and shift to a four‐coordinate silicon or gallium centre, respectively. Reported insertions of N‐heterocyclic silylenes into B−X bonds are rare and usually occur only for highly electronegative substituents X.^[11]^ Only one single example of a B−C bond scission is documented, in which silylene inserts into the B−C_6_F_5_ structural moiety.^[12]^ With this in mind, the ring expansion towards **10** is found facile for the more electropositive naphthalene platform and ring strain relaxation of **1** is again considered to be a significant driving force. On the contrary, the insertion of N‐heterocyclic gallylenes into B−X bonds has never been observed, and **12** provides the first example. In contrast to related **8**, the heavier silicon and gallium carbenoids lead to **10** and **12** adopting a *spiro*‐cyclic structure with the connecting metalloid between two five‐membered rings. In particular, the two angles at silicon or gallium, which enclose the five‐membered cycles, are close to or significantly lower than 90°. In particular, this holds for **12**, in which the angle values of 82.98(8)° for N5‐Ga1‐N2 and 88.44(9)° for C7‐Ga1‐B1 are found. DFT calculations support that the higher temperature required for these reactions is a consequence of the relatively high free enthalpy of the borete‐carbenoid adducts with respect to the separated species; see species **10**‐**int 1** and **12**‐**int 1** in the Supporting Information.

Further conversion of the *spiro*‐cyclic structures **10** and **12** in analogy to the formation of compound **8** is thermodynamically unfavourable. The calculated values of compounds **10**‐**hypothetical** (Si) and **12**‐**hypothetical** (Ga) reveal significantly higher free enthalpy (∼120–160 kJ/mol) than for **10** and **12**, respectively, see Supporting Information. This finding is also in line with the lack of stability for the three‐coordinate metalloid centre. While compound **8** is well stabilised by the cationic iminium moiety with C1=N3 double bond motive such a scenario would be unlikely for the hypothetical metalloid centres of silicon and gallium, for which π‐bonding is unfavourable.

## Conclusion

The four‐membered borete **1** was hypothesised to undergo ring expansion reactions within the context of donor‐acceptor functionalised cyclobutanes. Reactions of **1** with representative carbon, silicon, and gallium donor ligands confirmed the assumed behaviour and gave rise to complex cyclic systems, including new tetracyclic (**8**) and spirocyclic (**10**, **12**) compounds. The Baeyer strain of borete **1** was identified to have a significant contribution to the driving force of the ring expansion reactions, and we propose the donor‐acceptor concept of boracycles to be considered for the construction of complex boron architectures to a broader community.

## Experimental Section

Deposition Numbers 2145545 (for **6**), 2145546 (for **8**), 2145547 (for **10**), 2145548 (for **12**) contain the supplementary crystallographic data for this paper. These data are provided free of charge by the joint Cambridge Crystallographic Data Centre and Fachinformationszentrum Karlsruhe Access Structures service.

## Conflict of interest

The authors declare no conflict of interest.

1

## Supporting information

As a service to our authors and readers, this journal provides supporting information supplied by the authors. Such materials are peer reviewed and may be re‐organized for online delivery, but are not copy‐edited or typeset. Technical support issues arising from supporting information (other than missing files) should be addressed to the authors.

Supporting Information

## Data Availability

The data that support the findings of this study are available from the corresponding author upon reasonable request.
