# Supplementary material for: Reactions of a Four‐Membered Borete with Carbon, Silicon, and Gallium Donor Ligands: Fused and Spiro‐Type Boracycles
Source: Chemistry. 2022 Apr 21;28(32):e202200673. doi: 10.1002/chem.202200673 (PMC9322404; doi:10.1002/chem.202200673)
Supplement: Supplementary file 1 — Supporting Information [file CHEM-28-0-s001.pdf]

Chemistry–A European Journal

Supporting Information

Reactions of a Four-Membered Borete with Carbon, Silicon, and Gallium Donor Ligands: Fused and Spiro-Type Boracycles

Zeynep Güven, Lars Denker, Hadi Dolati, Daniela Wullschläger, Bartosz Trzaskowski,* and René Frank*

1. Synthetic and Analytical Procedures

1.1. General Information

All procedures were performed under dry nitrogen atmosphere using Schlenk techniques or in a glove box (M. Braun 200B model) unless stated otherwise. Solvents were purified and dried using a Solvent Purification System (M. Braun) and stored over sodium or potassium, except for halogenated solvents, which were stored over molecular sieves (3-4 Å). All commercially available compounds (Abcr, Deutero, Sigma Aldrich, TCI) were used without further purification. Deuterated solvents (C_6D_6, THF-D_8, toluene-D_8) were dried over sodium-potassium alloy, filtered and stored under nitrogen. CD_2Cl_2 and $CDCl_3$ were dried over CaH_2, distilled and stored under nitrogen. Compounds **1** [1], **7** [2], **9** [3] and **11** [4] were prepared according to literature methods. NMR spectra were recorded on Bruker Avance II-300, Avance III-HD, Avance III-400 and AVII-600 spectrometers. The chemical shifts (δ) are reported in parts per million (ppm).

For ^{11}B-NMR spectra an external calibration with $BF_3{\cdot}Et_2O$ was used. Coupling constants are stated in Hertz (Hz), multiplicities are defined as br (broad), s (singlet), d (doublet), t (triplet), q (quartet), qu (quintet), sept (septet) or m (multiplet). If necessary, 2D-NMR experiments (H,H-COSY, H,C-HSQC, H,C-HMBC) were used to aid the assignment of the signals.

Elemental analyses were accomplished by combustion and gas chromatographic analysis using a Vario MICRO Tube and HW detection. Values are reported in mass-%.

Optical measurements were carried out in quartz glass cuvettes. UV-VIS absorption spectra were recorded on a Varian Cary 50 device in dichloromethane of spectroscopic purity, which was dried over CaH_2.

2. Synthesis of Compounds

2.1. Synthesis of Compound 1

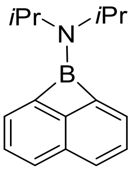

1

1,8-Dibromonaphthalene (4.00 g, 14.00 mmol, 1.0 eq.) was dissolved in diethyl ether (35 mL), and the mixture cooled to -78°C. *n*-Butyllithium (17.5 mL, 28.00 mmol, 2.0 eq., 1.6 M in hexane) was added dropwise to the mixture and stirred for 40 minutes, until a cloudy precipitate being formed. The reaction mixture was then warmed to room temperature and stirred for a further 10 minutes, until the solution became clear. The mixture was cooled to 0 °C and added dropwise to a cold (−20 °C) solution of diisopropylaminoboron dichloride (3.82 g, 21.00 mmol, 1.5 eq.) in hexane (30 mL). Lithium chloride started to precipitate, and the mixture was warmed to room temperature. After the solvent was removed in vacuum, the subsequent sublimation in a high vacuum at 90 °C gave the product as a colorless crystalline solid (2.46 g, 10.37 mmol, 74%).

^{1}H NMR (300 MHz, $CDCl_3$): δ (ppm) = 7.71 (dd, ^3J = 8.3, 0.7 Hz, 1 H, Naph*H*), 7.69 (dd, ^3J = 8.3, 0.7 Hz, 1 H, Naph*H*), 7.63-7.58 (m, ^3J = 8.3, 6.3 Hz, 2 H, Naph*H*), 7.45 (dd, 2 H, Naph*H*), 3.90 (sept, ^3J = 6.7 Hz, 2 H, $CH(CH_3)_2$), 1.50-1.46 [d, 12 H, $CH(CH_3)_2$].

^{11}B{^{1}H} NMR (96 MHz, $CDCl_3$): δ (ppm) = 37.4 (s, $\omega_{1/2}$ = 190 Hz).

Compound 1 was synthesized according to the reported preparation.[1] The analytical data obtained are in accordance with reported spectra.

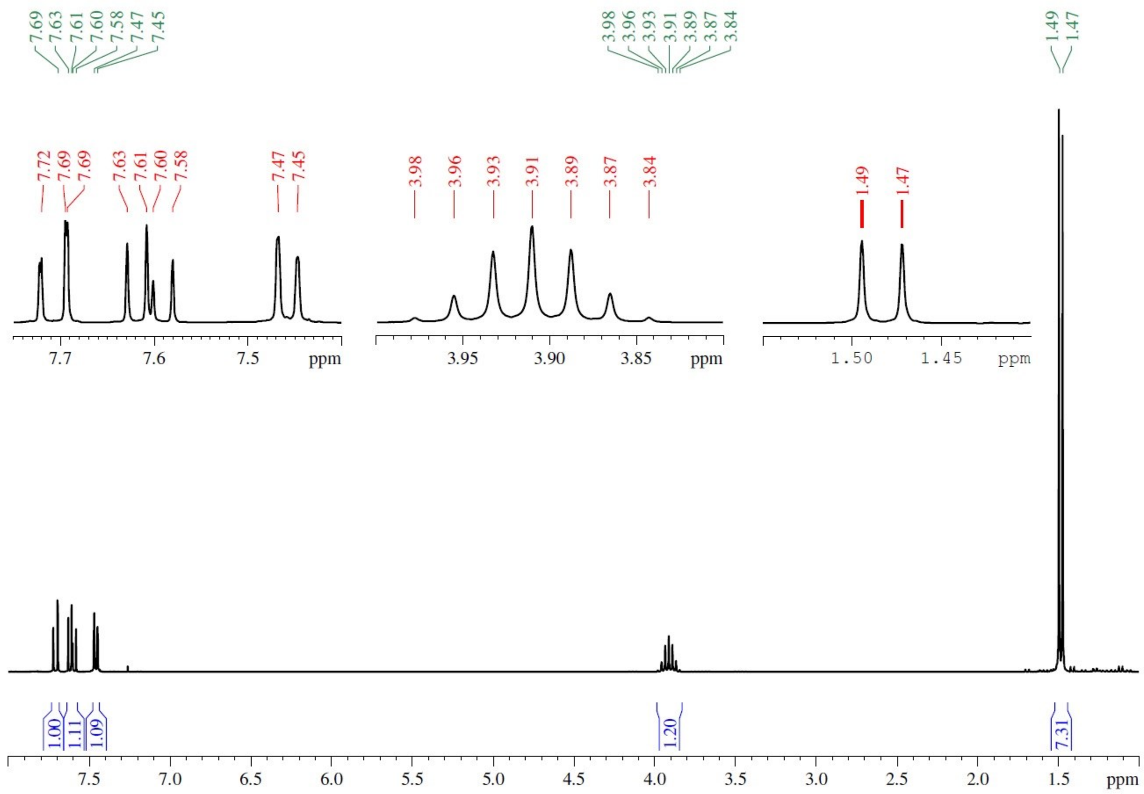

Figure S1 ^{1}H NMR spectrum of compound **1**, (300 MHz, $CDCl_3$).

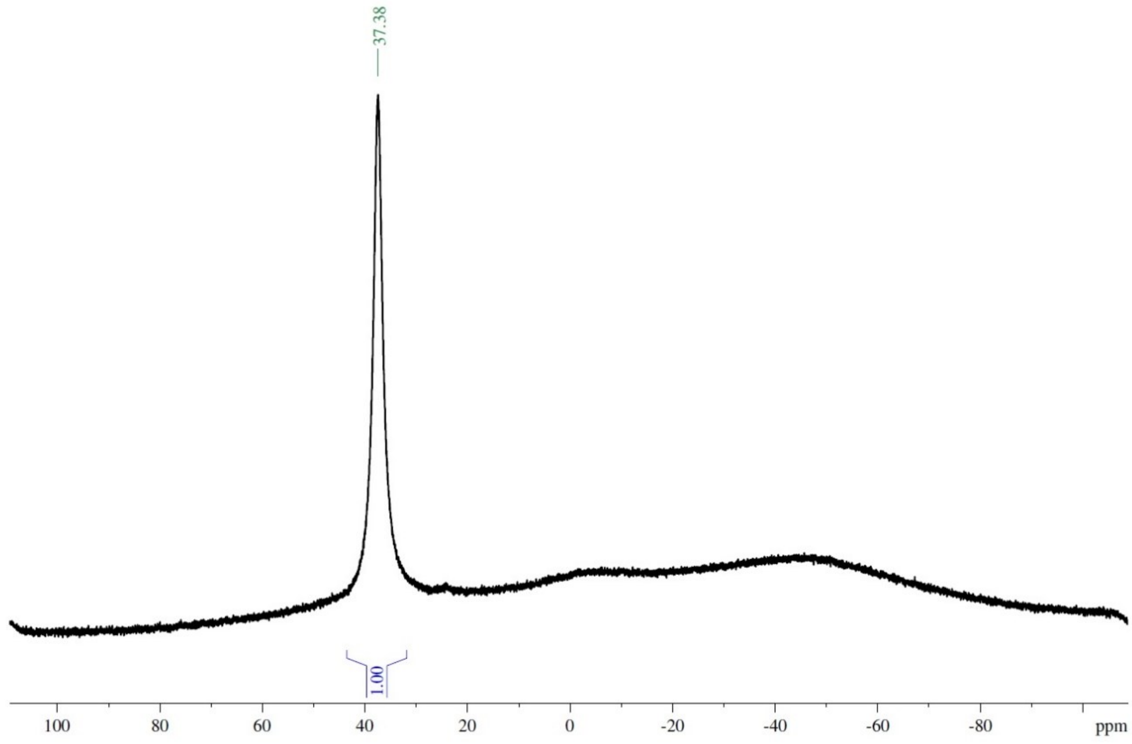

Figure S2: $^{11}B\{^{1}H\}$ NMR spectrum of compound **1**, (96 MHz, $CDCl_3$).

2.2. *Synthesis of Compound 6*

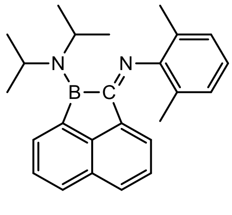

6

Compound **1** (200 mg, 0.84 mmol, 1.0 eq) and 2,6-Xylyl isocyanide (**5,** 112 mg, 0.84 mmol, 1.0 eq) were dissolved in toluene (1 mL) and allowed to stand at ambient temperature for 5 min. The product was recrystallized by diffusion of *n*-pentane into the solution. After decantation and washing of the residue with *n*-pentane the product **6** was obtained as yellow-orange crystals suitable for X-ray crystallography (232 mg, 0.63 mmol, 75%).

^{1}H-NMR (400 MHz, $CDCl_3$): *δ* (ppm) = 8.17 (br. s, 1 H, Naph*H*), 7.90-7.87 (m, 1 H, Naph*H*), 7.79-7.76 (m, 1 H, Naph*H*), 7.67-7.62 (m, 1 H, Naph*H*), 7.21-7.17 (m, 1 H, Naph*H*), 7.13-7.11 (m, 2 H, *m*-Xyl*H*), 7.00-6.96 (m, 1 H, *p*-Xyl*H*), 6.67-6.65 (m, 1 H, Naph*H*), 2.02 (s, 6 H, XylCH_3), 1.63 [br. s, 6 H, $CH(CH_3)_2$], 1.43 [br. s, 6 H, $CH(CH_3)_2$]. Not observed: C*H*(CH_3)

^{13}C{^{1}H}-NMR (75 MHz, $CDCl_3$): *δ* (ppm) = 152.2 (s, Xyl*C*), 147.3 (s, Naph*C*), 137.8 (s, Naph*C*), 131.9 (s, Naph*C*), 128.1 (4 × overlapped s, Naph*C* + 3 Naph*C*H), 127.8 (s, Naph*C*H), 127.7 (s, Xyl*C*H), 126.6 (s, Naph*C*H), 126.5 (s, Xyl*C*), 123.2 (s, Xyl*C*), 122.1 (s, *p*-Xyl*C*H), 120.5 (s, Naph*C*H), 47.5 [s, *C*H(CH_3)$_2$], 23.2 [s, br, CH(CH_3)$_2$], 18.1 (s, XylCH_3). Not observed: B−*C*-Naph and B−*C*=NXyl.

^{11}B{^{1}H} NMR (108 MHz, $CDCl_3$): δ (ppm) = 35.8 (s, $\omega_{½}$ = 408 Hz).

Elemental Analysis:	Calculated for $C_{25}H_{29}BN_2$:	C 81.52, H 7.94, N 7.61.
	Found:	C 82.05, H 7.85, N 7.65.

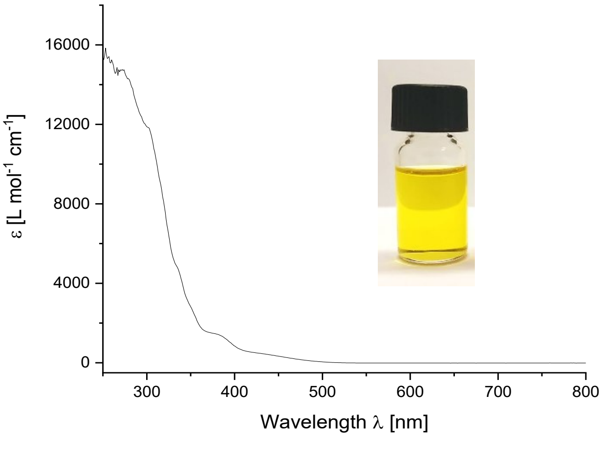

Figure S3: UV-VIS absorption spectrum of compound **6** dissolved in dichloromethane and image of the recorded solution.

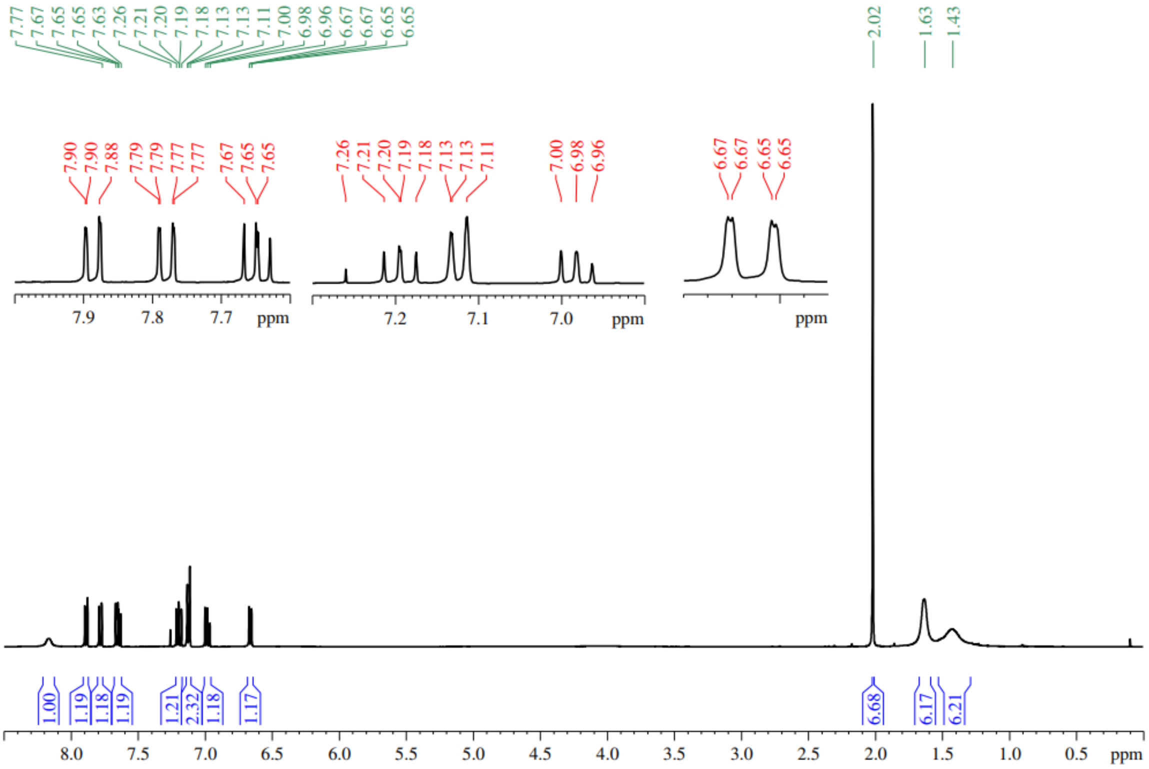

Figure S4: ^{1}H NMR spectrum of compound **6**, (400 MHz, $CDCl_3$).

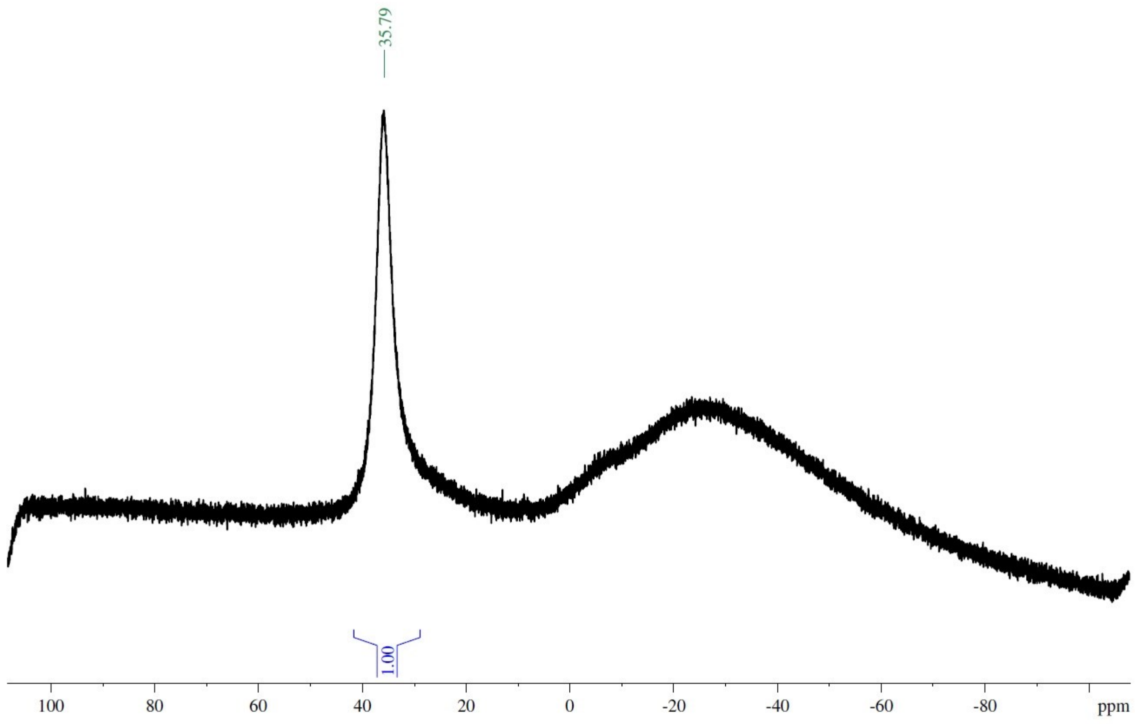

Figure S5: $^{11}B\{^{1}H\}$ NMR spectrum of compound **6**, (128 MHz, $CDCl_3$).

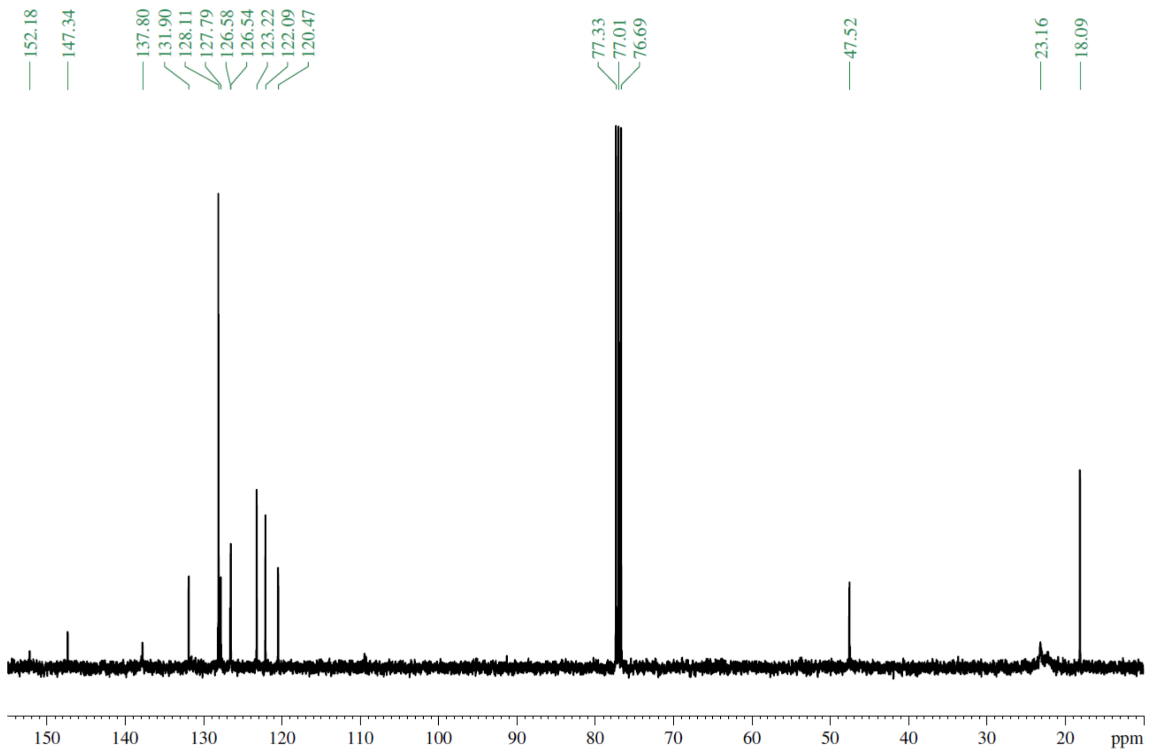

Figure S6: $^{13}C\{^1H\}$ NMR spectrum of compound **6**, (75 MHz, $CDCl_3$).

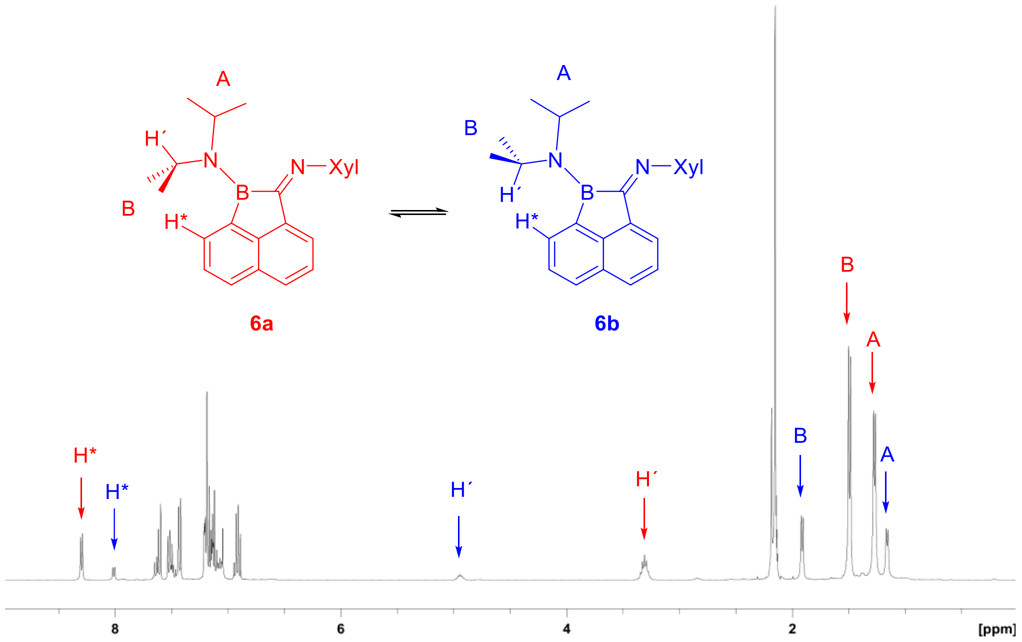

Figure S7: ^1H NMR spectrum of compound **6**, (600 MHz, toluene-D_8, −60 °C). Xyl = 2,6-dimethylphenyl.

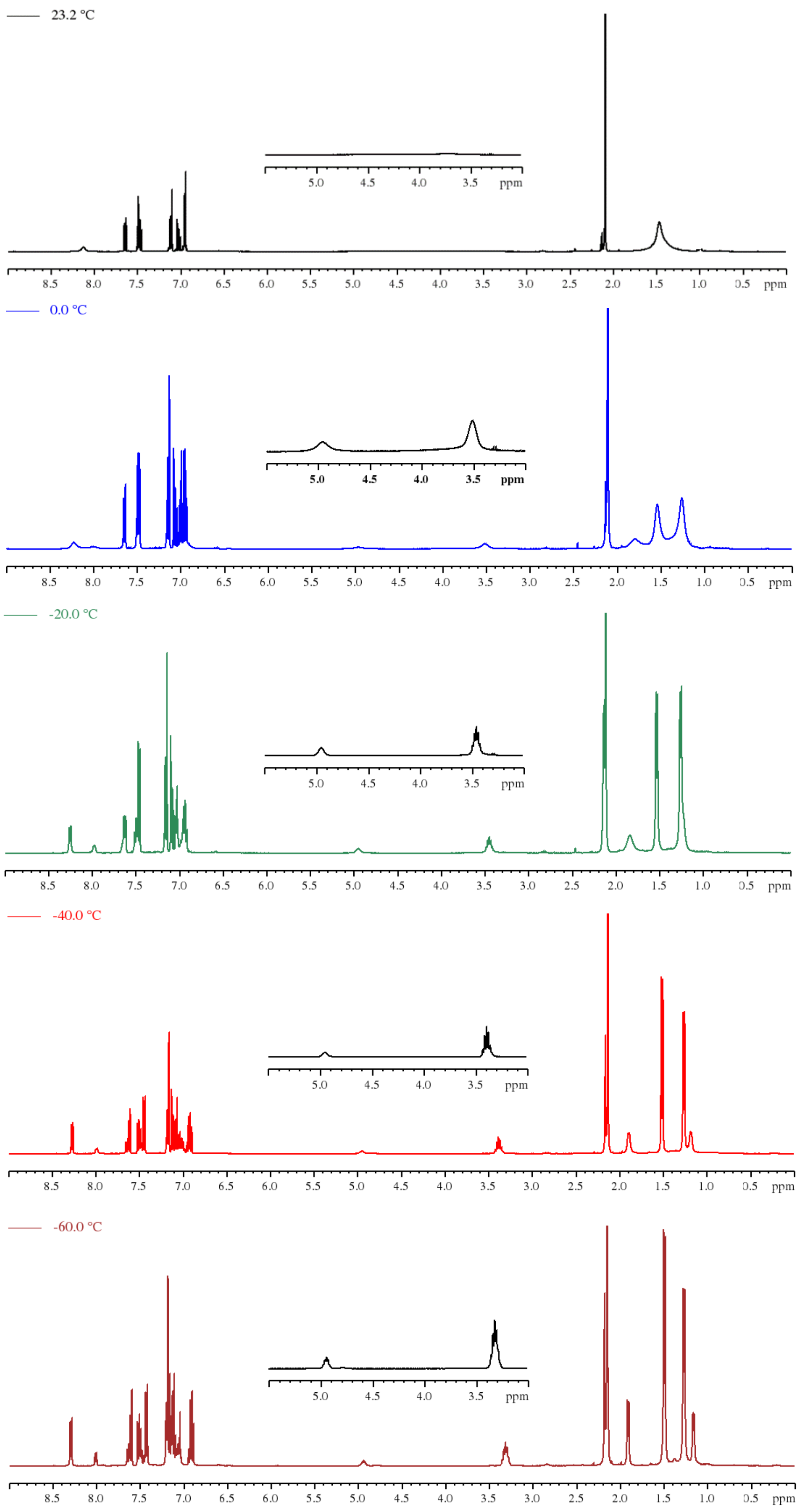

Figure S8: ^{1}H NMR spectra of compound **6**, (600 MHz, toluene-D$_8$, variable temperature).

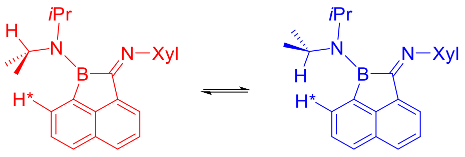

6a **6b**

Table S1: Temperature dependent integrals in vt ^{1}H NMR spectra. H* and H* reflect the assigned signals as indicated in Figure S7. Xyl = 2,6-dimethylphenyl.

θ [°C]	T [K]	**6a** Integral H*	**6b** Integral H*	ln K
-10	263.15	1	0.5111	-1.3643
-20	253.15	1	0.4801	-1.4269
-30	243.15	1	0.4453	-1.5022
-40	233.15	1	0.4085	-1.5884
-50	223.15	1	0.3650	-1.7010
-60	213.15	1	0.3312	-1.7982
-70	203.15	1	0.3030	-1.8872
-80	193.15	1	0.2761	-1.9801

Equilibrium constant: K = [B] / 2[A]

The factor 2 in the denominator is applied due to the formation of two degenerated forms of **6b**, which cannot be distinguished in the ^{1}H NMR spectra.

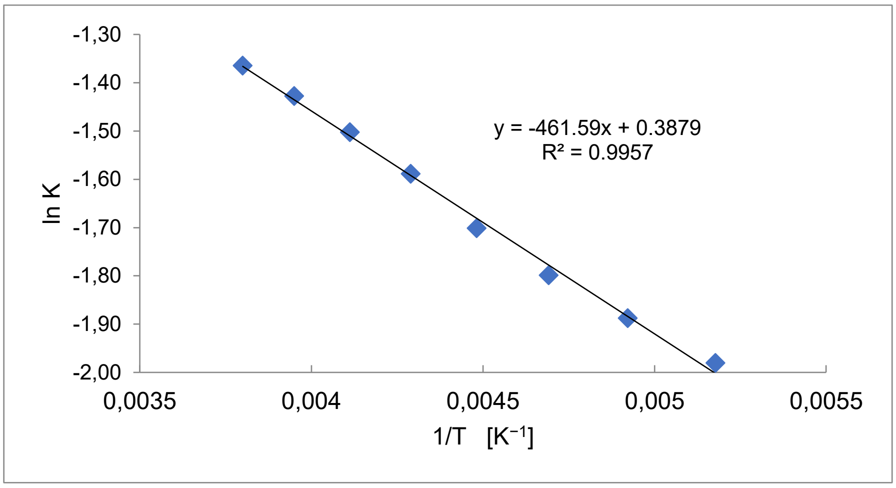

Figure S9: The plot of ln K vs. 1/T (van´t Hoff plot) as calculated according to table S1 gives a linear correlation.

For the rotational process **6a** → **6b** the thermodynamic value for ΔH° and ΔS° at standard conditions (T = 298.15 K, p = 1.01325 bar, a = 1 M) were determined as stated:

Enthalpy: ΔH° = 3.8 ± 1.3 kJ/mol and Entropy: ΔS° = 3.2 ± 1.5 kJ/mol

2.3. *Synthesis of Compound 8*

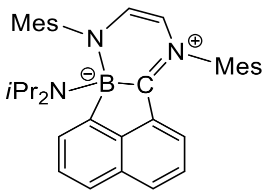

8

Compound **1** (100 mg, 0.42 mmol, 1.0 eq.) and IMes (**7**, 135 mg, 0.44 mmol, 1.05 eq) were dissolved in toluene (1 mL). The solution turned immediately dark red. The product was recrystallized by adding *n*-hexane (5 mL) and cooling the solution at 5 °C overnight. After decantation and washing of the residue with *n*-hexane product **8** was obtained as dark red crystals suitable for X-ray crystallography (120 mg, 0.22 mmol, 53%).

^{1}H-NMR (400 MHz, CDCl$_3$): *δ* (ppm) = 7.63 (dd, ^{3}J = 8.3 Hz, ^3J = 0.7 Hz, 1 H, Naph*H*), 7.41 (dd, ^{3}J = 8.3 Hz, ^{3}J = 0.7 Hz, 1 H, Naph*H*), 7.15-7.10 (m, 1 H, Naph*H*), 7.06-6.95 (m, 3 H, Naph*H*), 6.79 (m, 2 H, *m*-Mes*H*), 6.64 (m, 1 H, *m*-Mes*H*), 6.59 (d, ^{3}J = 4.8 Hz, 1 H, CHC*H*), 6.05 (dd, ^{3}J = 7.3 Hz, ^{3}J = 0.8 Hz, 1 H, *m*-Mes*H*), 5.51 (d, ^{3}J = 4.8 Hz, 1 H, C*H*CH,), 3.16 (sept, ^{3}J = 6.6 Hz, 2 H, C*H*(CH$_3$)$_2$), 2.77 (s, 3 H, MesC*H*$_3$), 2.48 (s, 3 H, MesC*H*$_3$), 2.32 (s, 3 H, MesC*H*$_3$), 2.25 (s, 3 H, MesC*H*$_3$), 1.40 (s, 3 H, MesC*H*$_3$), 1.26 (s, 3 H, MesC*H*$_3$), 0.83 [d, ^{3}J = 6.6 Hz, 6 H, CH(C*H*$_3$)$_2$], 0.47 [br. s, 6 H, CH(C*H*$_3$)$_2$].

^{13}C{^{1}H}-NMR (100 MHz, CDCl$_3$): δ (ppm) = 145.3 (s, Mes*C*), 142.5 (s, Naph*C*), 139.9 (s, Mes*C*), 139.4 (s, *C*H=CH), 139.1 (s, Mes*C*), 137.7 (s, Naph*C*), 136.6 (s, Mes*C*), 135.6 (s, Mes*C*), 133.5 (s, Mes*C*), 133.2 (s, Mes*C*), 131.5 (s, Naph*C*), 130.3 (s, m-Mes*C*H), 129.9 (s, Naph*C*H), 129.5 (s, Naph*C*H), 128.7 (s, Naph*C*H), 128.7 (s, m-Mes*C*H), 128.3 (s, m-Mes*C*H), 126.9 (s, Naph*C*H), 125.6 (s, Naph*C*H), 122.3 (s, Naph*C*H), 120.8 (s, m-Mes*C*H), 104.5 (s, CH=*C*H), 49.0 [br. s, *C*H(CH$_3$)$_2$], 26.3 [br. s, CH(*C*H$_3$)$_2$], 24.5 [br. s, CH(*C*H$_3$)$_2$], 21.2 (s, Mes*C*H$_3$), 21.1 (s, Mes*C*H$_3$), 20.7 (s, Mes*C*H$_3$), 19.9 (s, Mes*C*H$_3$), 18.0 (s, Mes*C*H$_3$), 17.5 (s, Mes*C*H$_3$). Not observed: B−*C*(Naph) and B−*C*R$_2^+$.

^{11}B{^{1}H}-NMR (108 MHz, CDCl$_3$): *δ* (ppm) = –1.2 (s, $\omega_{½}$ = 85 Hz).

Elemental Analysis:	Calculated for $C_{37}H_{44}BN_3$:	C 82.06, H 8.19, N 7.76.
	Found:	C 81.67, H 8.18, N 7.42.

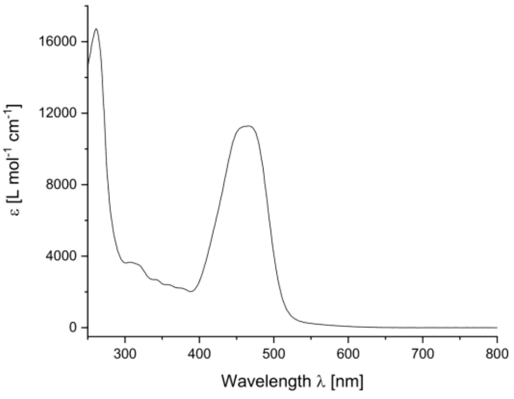

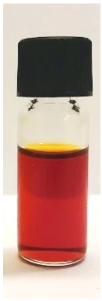

Figure S10: UV-VIS absorption spectrum of compound **8** in dichloromethane dissolved in dichloromethane and image of the recorded solution.

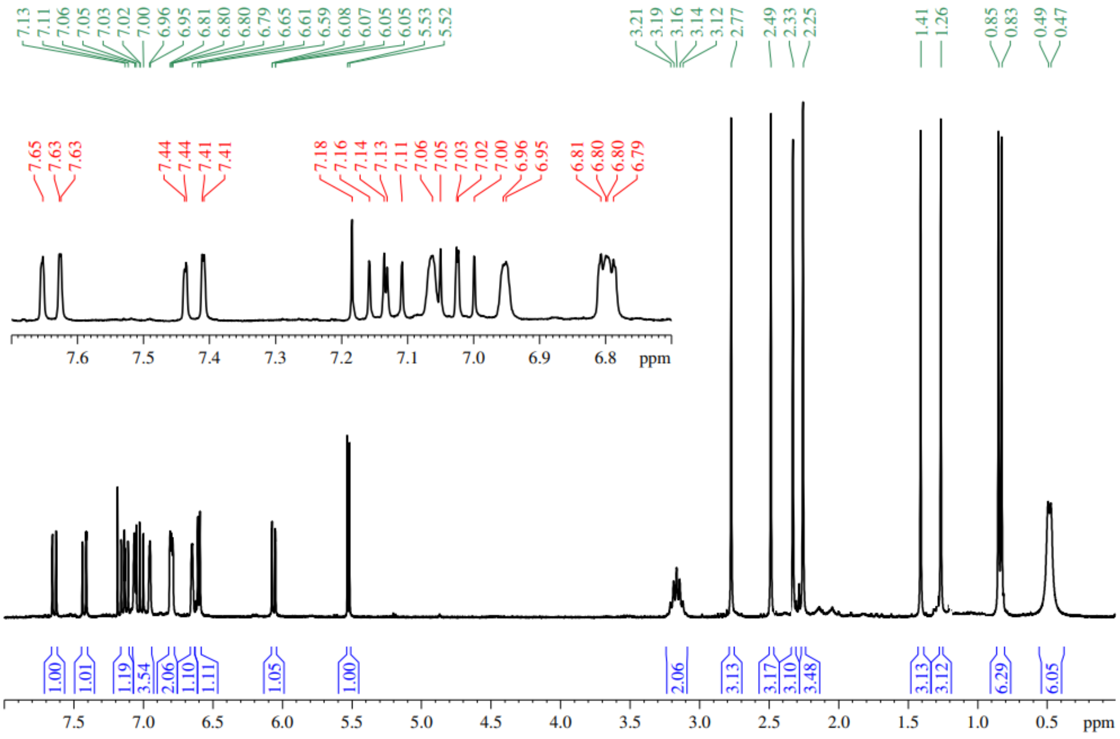

Figure S11: ^{1}H NMR spectrum of compound **8**, (300 MHz, $CDCl_3$).

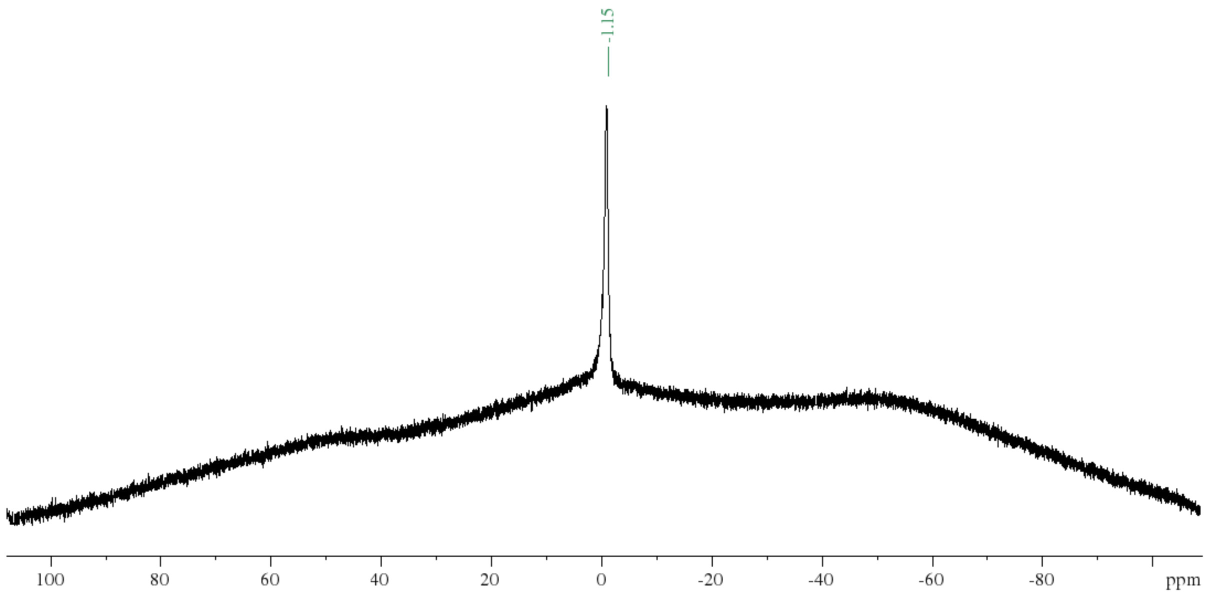

Figure S12: $^{11}B\{^{1}H\}$ NMR spectrum of compound **8**, (96 MHz, $CDCl_3$).

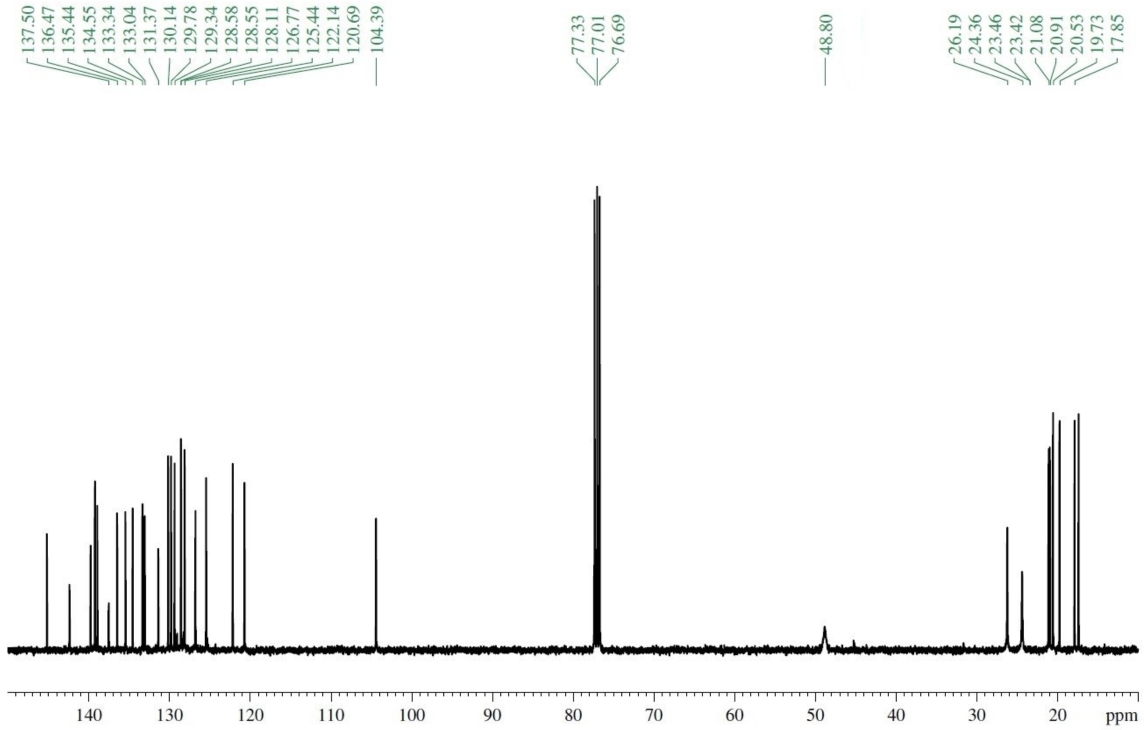

Figure S13: $^{13}C\{^{1}H\}$ NMR spectrum of compound **8**, (100 MHz, $CDCl_3$).

2.4. *Synthesis of Compound 10*

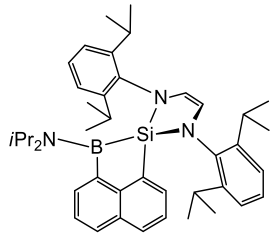

10

Compound **1** (200 mg, 0.84 mmol) dissolved in toluene (10 mL), and *N*-heterocyclic silylene **9** (700 mg, 1.73 mmol) dissolved in toluene (10 mL) was added to the solution. The mixture was stirred overnight at 80 °C. After evaporation of all volatiles under reduced pressure, the residue was extracted with pentane and concentrated to afford product **10** as pale yellowish crystals suitable for X-ray crystallography (160 mg, 0.25 mmol, 55%).

^{1}H NMR (300 MHz, C_6D_6): δ (ppm) = 8.22 (dd, ^{3}J = 6.7 Hz, 1 H, Naph*H*), 7.81 [br, s, 1 H, N−C*H*(CH_3)$_2$], 7.61 (d, ^{3}J = 8.3 Hz, 1 H, Naph*H*), 7.57 (d, ^{3}J = 8.4 Hz, 1 H, Naph*H*), 7.32 (dd, ^{3}J = 8.3 Hz, ^{3}J = 7.5 Hz, 1 H, Naph*H*), 7.18 (m, 2 H, Dipp*H*), 7.00 (m, 6 H, 4 Dipp*H* + 2 Naph*H*), 5.98 (s, C*H*=C*H*), 4.30 [br, s, 1 H, N−C*H*(CH_3)$_2$], 3.78 [sept, ^{3}J = 6.5 Hz, 2 H, C*H*(CH_3)$_2$], 3.60 [sept, ^{3}J = 6.7 Hz, 2 H, C*H*(CH_3)$_2$], 1.36 [d, ^{3}J = 6.5 Hz, 6 H, N−CH*(CH_3)*$_2$], 1.19 [m, 18 H, 1 N−CH*(CH_3)*$_2$ + 2 CH*(CH_3)*$_2$], 0.94 [br. d, ^{3}J = 6.6 Hz, 6 H, CH*(CH_3)*$_2$), 0.6 [d, ^{3}J = 6.7 Hz, 6 H, CH*(CH_3)*$_2$].

^{13}C{^{1}H} NMR (75 MHz, C_6D_6): δ (ppm) = 149.2 (s, br, Naph*C*), 147.6 (s, Dipp*C*), 147.1 (s, Dipp*C*), 142.2 (s, Dipp*C*), 140.3 (s, br, Naph*C*), 133.7 (s, Si−*C*), 131.0 (s, Naph*C*H), 130.5 (s, Naph*C*H), 130.0 (s, Naph*C*H), 126.67 (s, Naph*C*H), 126.3 (s, Naph*C*H), 125.7 (s, Naph*C*H), 125.0 (s, Dipp*C*H), 124.9 (s, Dipp*C*H), 124.4 (s, Dipp*C*H), 122.0 (*C*H=*C*H), 29.3 and 29.2 [both, s, NCH(*C*H$_3$)$_2$], 27.4, 26.1, 25.3 and 25.2 [all four, s, Ar-CH(*C*H$_3$)$_2$], 25.0 [s, Ar-*C*H(CH_3)$_2$, overlapped signal]. Not Observed: B−N*C*H(CH_3)$_2$.

^{11}B{^{1}H} NMR (96 MHz, C_6D_6): *δ* (ppm) = 44.5 ($\omega_{1/2}$ = 1033 Hz).

Elemental Analysis:	Calculated for $C_{42}H_{56}BN_3Si$:	C 78.60, H 8.79, N 6.55.
	Found:	C 79.05, H 8.65, N 6.40.

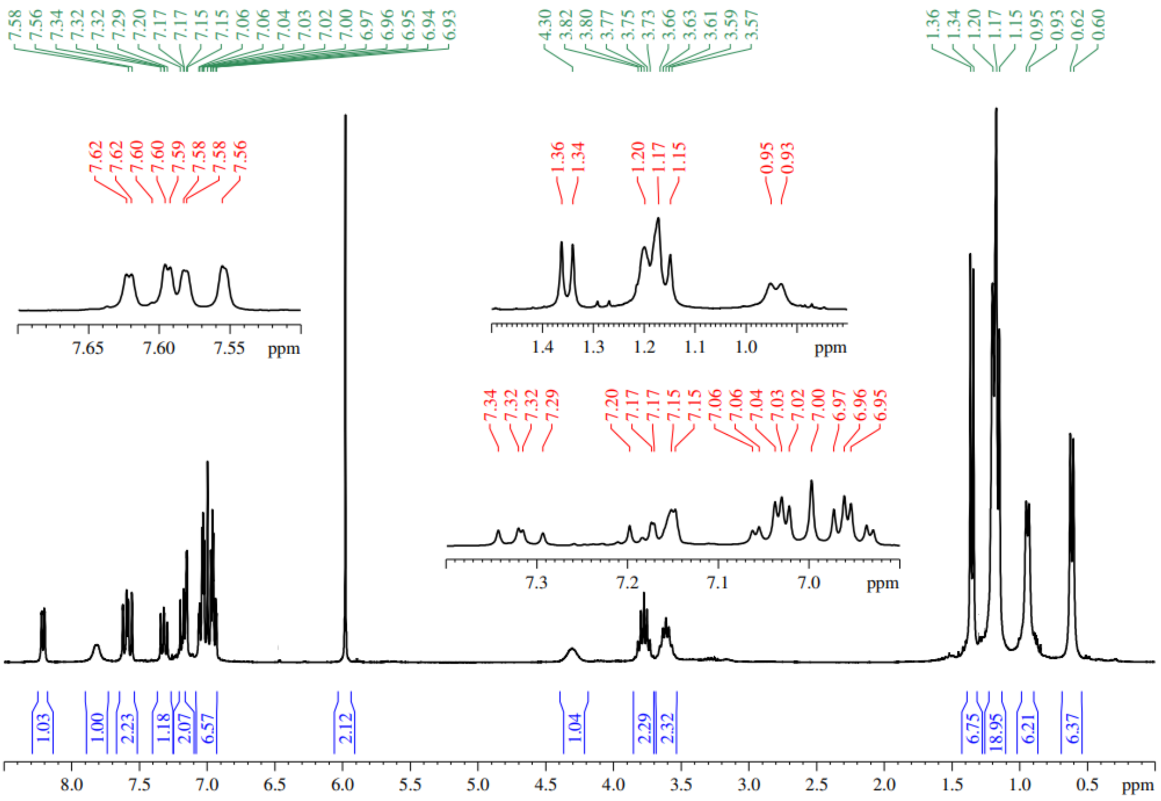

Figure S14: ^{1}H NMR spectrum of compound **10**, (300 MHz, C_6D_6).

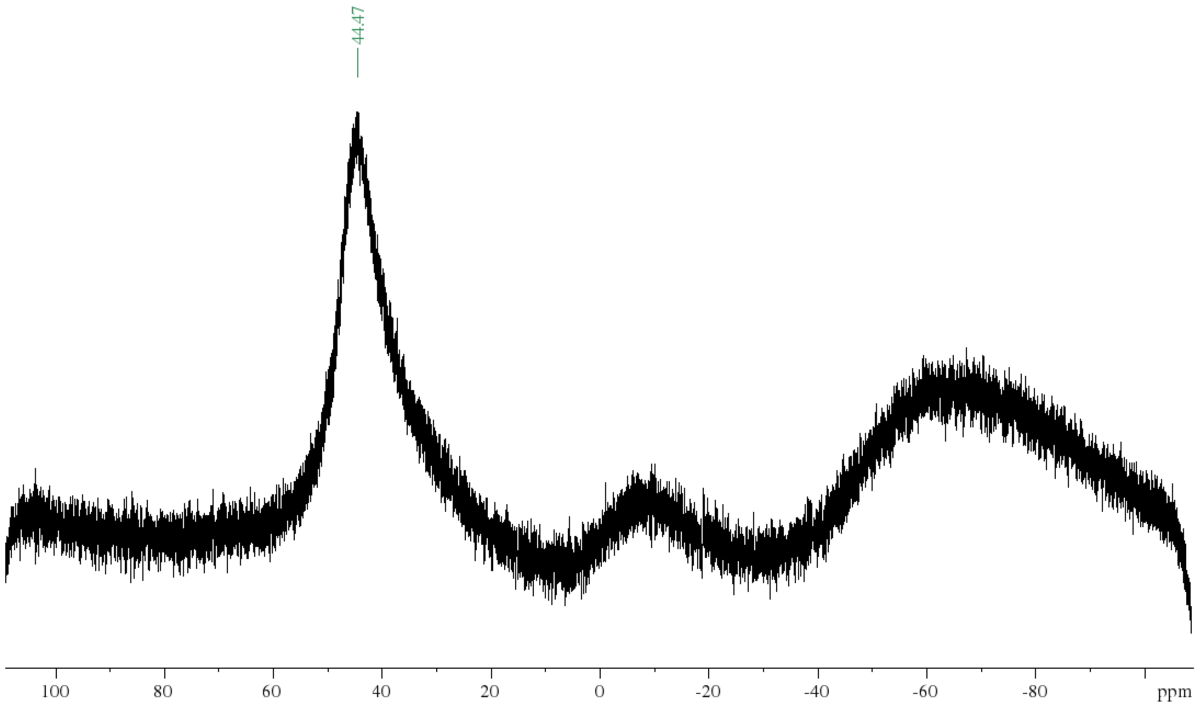

Figure S15: $^{11}B\{^{1}H\}$ NMR spectrum of compound **10**, (96 MHz, C_6D_6).

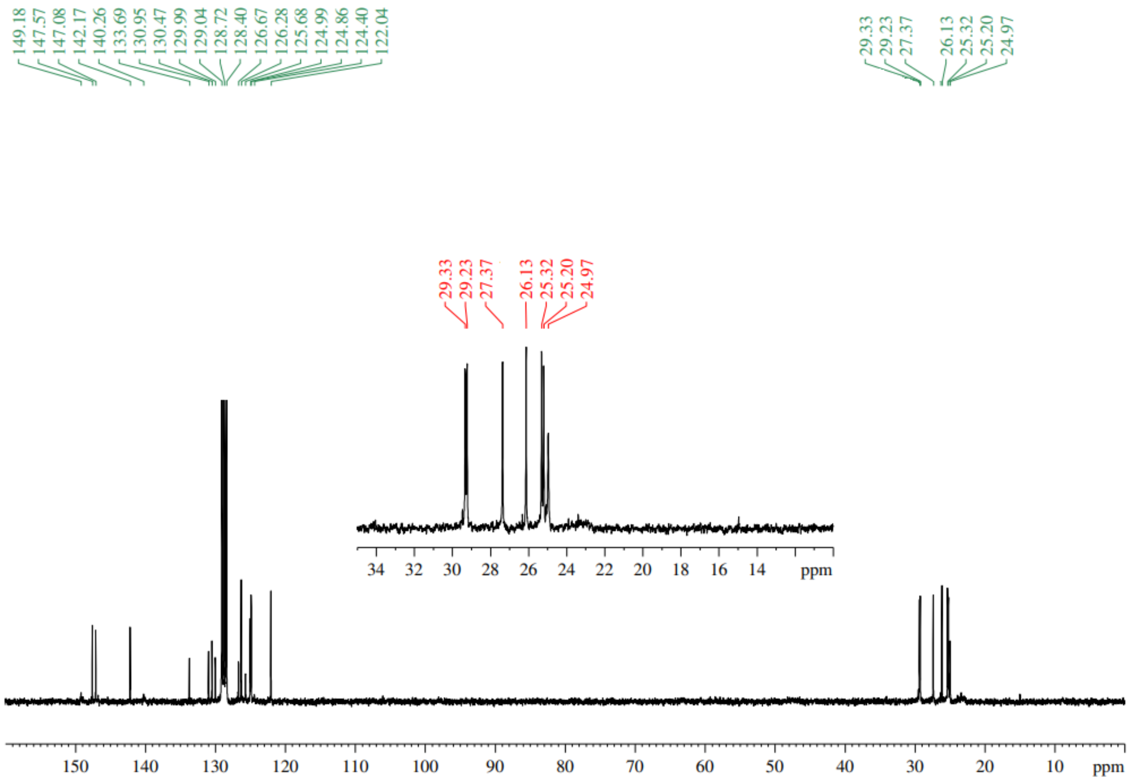

Figure S16: $^{13}C\{^{1}H\}$ NMR spectrum of compound **10**, (75 MHz, C_6D_6).

2.5. *Synthesis of Compound 12*

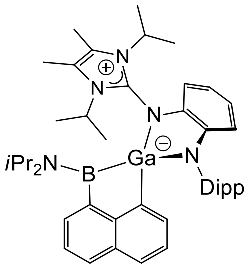

12

Compound **1** (72 mg, 0.30 mmol) dissolved in THF (1 mL), and Ga(AmIm) (156 mg, 0.30 mmol) was added to the solution. The mixture was stirred overnight at 50°C. Storage of the solution at −40 °C afforded product **12** as yellowish crystals suitable for X-ray crystallography (125 mg, 0.17 mmol, 55%).

^{1}H NMR (300 MHz, THF-D$_8$): δ (ppm) = 7.62 (m, 2 H, Naph*H*), 7.51 (m, 2 H, Naph*H*), 7.22 (m, 2 H, Naph*H*), 7.02 (dd, ^3J = 7.2 Hz 3.1 Hz, 1 H, Dipp*H*), 6.9 (m, 2 H, Dipp*H*), 6.39 (td, ^3J = 5.1, 2.7 Hz, 1 H, C3-*H*), 6.15 (td, ^3J = 5.1, 2.7 Hz, 1 H, C2-*H*), 6.02 (m, ^3J = 5.12 Hz, 1 H, C1-*H*), 5.84 (m, ^3J = 5.61 Hz, 1 H, C4-*H*), 4.88 [m, 2 H, signal overlap 1 BN*CH*(CH$_3$)$_2$ and 1 ImN*CH*(CH$_3$)$_2$], 4.40 [sept, ^3J = 5.44 Hz, 1 H, Im-N*CH*(CH$_3$)$_2$], 3.55 (sept, ^3J = 6.34 Hz, 1 H, BN*CH*(CH$_3$)$_2$), 3.45 [sept, ^3J = 6.5 Hz, 1 H, Ar-*CH*(CH$_3$)$_2$], 3.20 [sept, ^3J = 6.8 Hz, 1 H, Ar-*CH*(CH$_3$)$_2$], 2.10 (s, 3 H, C=C-CH$_3$), 1.95 (s, 3 H, C=C-CH$_3$), 1.40 (d, ^3J = 7.02 Hz, 3 H, NCH(C*H$_3$*)$_2$), 1.30 (m, 6 H, CH$_3$), 1.18 (m, 12 H, ArCH(C*H$_3$*)$_2$), 1.05 (d, ^3J = 7.02 Hz, 3 H, CH$_3$), 0.98 (d, ^3J = 7.00 Hz, 3 H, CH$_3$), 0.93 [d, ^3J = 7.00 Hz, 3 H, CH$_3$], 0.88 [d, ^3J = 7.02 Hz, 3 H, CH$_3$], 0.81 (d, ^3J = 6.30 Hz, 3 H, CH$_3$], 0.41 [d, ^3J = 7.02 Hz, 3 H, BNCH*(CH$_3$)$_2$*], 0.14 [d, ^3J = = 7.0 Hz, 3 H, BN*C*H(C*H$_3$*)$_2$]

^{13}C{^{1}H}-NMR (100 MHz, THF-D$_8$): δ (ppm) = 163.1 (s, C, CN$_3$), 150.2, 150.0 (both, s, C), 149.5, 148.3, 146.6, 146.4, 141.8, 133.9 (all six, s, C), 131.2, 129.1, 127.2, 125.3, 125.2, 124.2, 123.9, 123.8, 123.5 (all nine, s, CH), 121.8, 121.5 (both, s, C), 113.4, 113.1, 113.0 (all three, CH), 50.9 (br, s, 2×BN*C*HMe$_2$,), 49.7, 49.2 (both, ImN*C*HMe$_2$), 28.6, 28.2 (both, s, *C*HMe$_2$), 27.4 [br, s, BNCH(*C*H$_3$)$_2$], 25.1, 23.3 (both, s, CH$_3$), 23.0 [br, s, BNCH(*C*H$_3$)$_2$], 21.8, 21.6, 20.5, 20.4, 9.9, 9.8 (all six, s, CH$_3$). Not observed: B−*C*.

^{11}B{^{1}H} NMR (96 MHz, THF-D$_8$): *δ* (ppm) = 53.5 ($\omega_{1/2}$ = 1394 Hz).

Elemental Analysis:	Calculated for $C_{59}H_{77}BGaN_5$:	C 75.64, H 8.28, N 7.48.
	Found:	C 75.15, H 8.35, N 7.40.

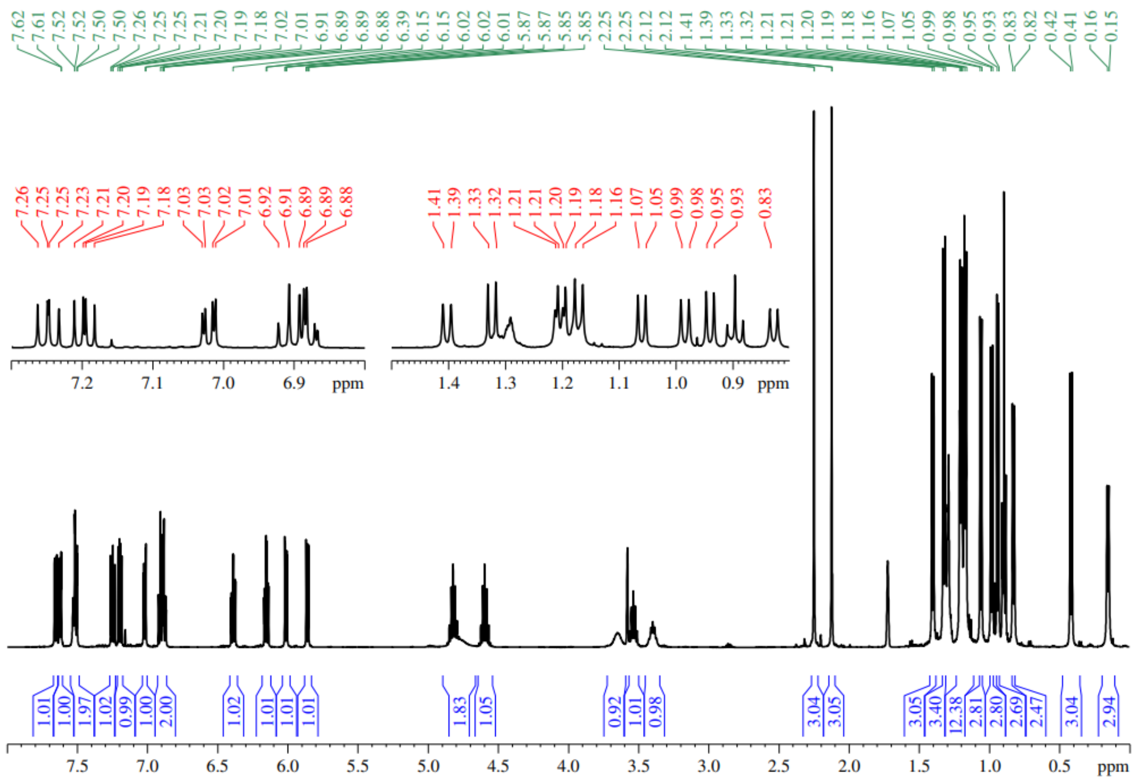

Figure S17: ^1H NMR spectrum of compound **12**, (300 MHz, THF-D_8).

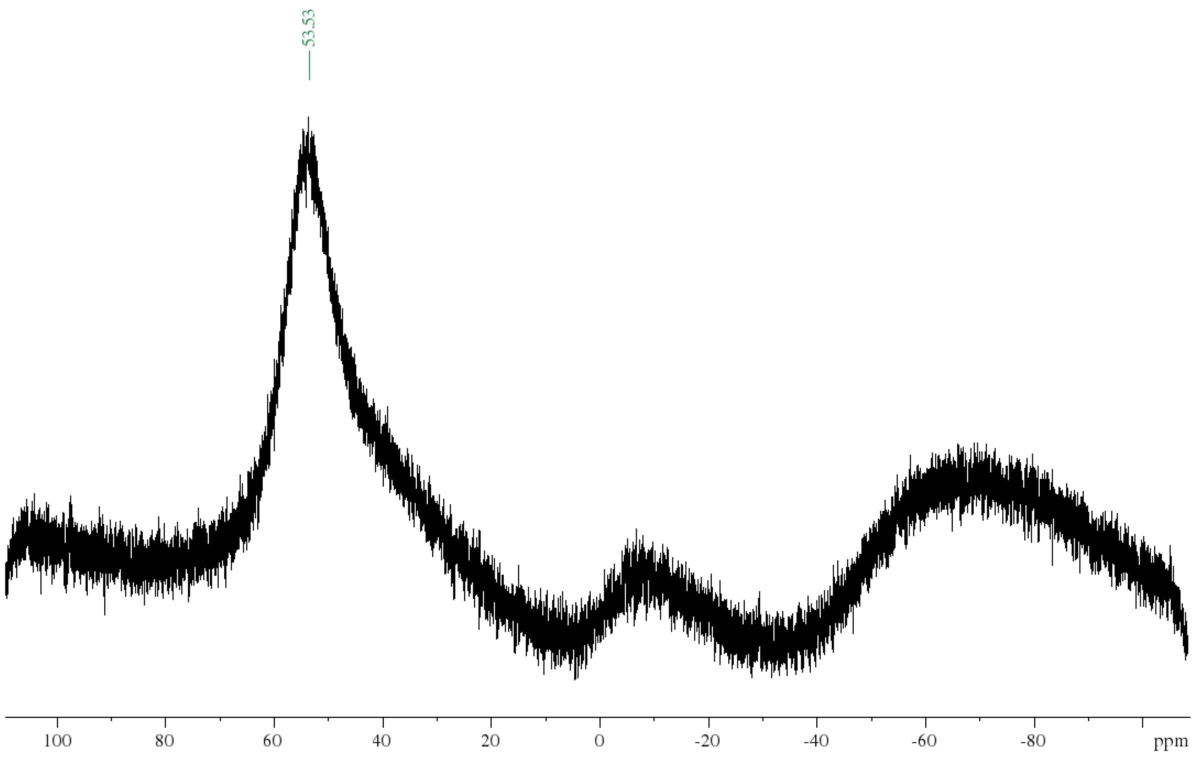

Figure S18: $^{11}B\{^1H\}$ NMR spectrum of compound **12**, (96 MHz, THF-D_8).

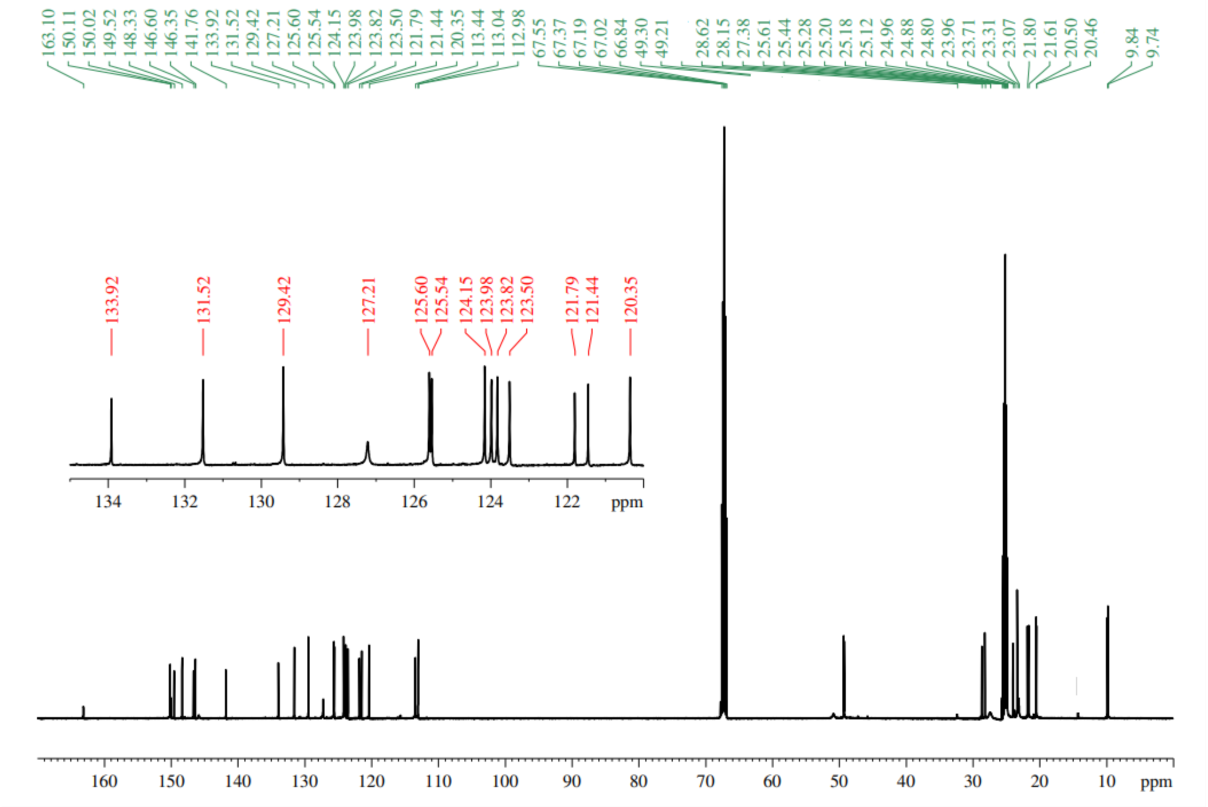

Figure S19: $^{13}C\{^1H\}$ NMR spectrum of compound **12**, (125 MHz, THF-D$_8$).

3. X-ray Crystallographic Information

3.1. Crystallographic Data for Compound 6

CCDC entry code:	2145545	
Empirical formula:	$C_{25}H_{29}BN_2$	
Formula weight:	368.31	
Temperature:	100(2) K	
Wavelength:	1.54184 Å	
Crystal system:	Monoclinic	
Space group:	$P2_1$	
Unit cell dimensions:	a = 10.184(10) Å	α = 90°.
	b = 7.981(5) Å	β = 101.59(1)°.
	c = 13.22030(10) Å	γ = 90°.
Volume:	1052.621(8) $Å^3$	Z = 2
Density (calculated):	1.162 g/cm^3	
Absorption coefficient:	0.504 mm^{-1}	
F(000):	396	
Θ-range for data collection:	3.413 to 77.707°.	
Index ranges:	-12<=h<=12, -10<=k<=10, -15<=l<=16	
Reflections collected:	85163	
Independent reflections:	4412 [R(int) = 0.0331]	
Completeness to θ = 67.684°:	100.0 %	
Absorption correction:	Semi-empirical from equivalents	
Max. and min. transmission:	1.00000 and 0.34302	
Refinement method:	Full-matrix least-squares on F^2	
Data / restraints / parameters:	4412 / 1 / 259	
Goodness-of-fit on F^2:	1.051	
Final R indices [I>2sigma(I)]:	R_1 = 0.0329, wR_2 = 0.0885	
R indices (all data):	R_1 = 0.0332, wR_2 = 0.0888	
Absolute structure parameter:	-0.13(6)	
Largest diff. peak and hole:	0.417 and -0.210 e.$Å^{-3}$	

3.2. Crystallographic Data for Compound 8

CCDC entry code:	2145546	
Empirical formula:	$C_{37}H_{44}BN_3$	
Formula weight:	541.56	
Temperature:	100(2) K	
Wavelength:	1.54184 Å	
Crystal system:	Triclinic	
Space group:	*P*-1	
Unit cell dimensions:	a = 10.53010(10) Å	α = 90.8680(10)°.
	b = 12.2000(2) Å	β = 94.0680(10)°.
	c = 12.5325(2) Å	γ = 107.4290(10)°.
Volume:	1531.11(4) $Å^3$	Z = 2
Density (calculated):	1.175 g/cm^3	
Absorption coefficient:	0.511 mm^{-1}	
F(000):	584	
θ-range for data collection:	3.538 to 77.543°.	
Index ranges:	-13<=h<=13, -15<=k<=15, -15<=l<=14	
Reflections collected:	31540	
Independent reflections:	6369 [R(int) = 0.0262]	
Completeness to θ = 67.684°:	100.0 %	
Refinement method:	Full-matrix least-squares on F^2	
Data / restraints / parameters:	6369 / 0 / 410	
Goodness-of-fit on F^2:	1.057	
Final R indices [I>2sigma(I)]:	R_1 = 0.0422, wR_2 = 0.1175	
R indices (all data):	R_1 = 0.0452, wR_2 = 0.1201	
Largest diff. peak and hole:	0.290 and -0.274 e.$Å^{-3}$	

3.3. *Crystallographic Data for Compound 10*

CCDC entry code:	2145547	
Empirical formula:	$C_{42}H_{56}BN_3Si$	
Formula weight:	641.79	
Temperature:	100(2) K	
Wavelength:	0.71073 Å	
Crystal system:	Monoclinic	
Space group:	$P2_1/c$	
Unit cell dimensions:	α = 10.7182(4) Å	α = 90°.
	b = 17.6784(4) Å	β = 125.349(5)°.
	c = 24.3615(9) Å	γ = 90°.
Volume:	3765.0(3) $Å^3$	Z = 4
Density (calculated):	1.132 g/cm^3	
Absorption coefficient:	0.095 mm-1	
F(000):	1392	
θ range for data collection:	2.050 to 32.502°.	
Index ranges:	-15<=h<=16, -25<=k<=25, -36<=l<=35	
Reflections collected:	120950	
Independent reflections:	12162 [R(int) = 0.0221]	
Completeness to θ = 25.242°:	100.0 %	
Refinement method:	Full-matrix least-squares on F^2	
Data / restraints / parameters:	12162 / 0 / 436	
Goodness-of-fit on F^2:	1.037	
Final R indices [I>2sigma(I)]:	R_1 = 0.0390, wR_2 = 0.1079	
R indices (all data):	R_1 = 0.0444, wR_2 = 0.1111	
Largest diff. peak and hole:	0.467 and -0.226 e.$Å^{-3}$	

3.4. Crystallographic Data for Compound 12

CCDC entry code:	2145548	
Empirical formula:	$C_{59}H_{77}BGaN_5$	
Formula weight:	936.78	
Temperature:	100(2) K	
Wavelength:	0.71073 Å	
Crystal system:	Orthorhombic	
Space group:	$P2_12_12_1$	
Unit cell dimensions:	a = 11.0104(2) Å	α = 90°.
	b = 20.8096(4) Å	β = 90°.
	c = 22.8480(5) Å	γ = 90°.
Volume:	5234.98(18) $Å^3$	Z = 4
Density (calculated)	1.189 g/cm^3	
Absorption coefficient	0.568 mm^{-1}	
F(000):	2008	
θ range for data collection:	2.034 to 32.517°.	
Index ranges:	-16<=h<=16, -31<=k<=31, -33<=l<=34	
Reflections collected:	273526	
Independent reflections:	17487 [R(int) = 0.0651]	
Completeness to θ = 25.242°:	99.9 %	
Refinement method:	Full-matrix least-squares on F^2	
Data / restraints / parameters:	17487 / 0 / 611	
Goodness-of-fit on F^2:	1.045	
Final R indices [I>2sigma(I)]:	R_1 = 0.0438, wR_2 = 0.0933	
R indices (all data):	R_1 = 0.0550, wR_2 = 0.0967	
Absolute structure parameter:	-0.002(2)	
Largest diff. peak and hole:	1.123 and -0.377 e.$Å^{-3}$	

4. Computational studies

4.1 Computational methods

All calculations were performed using the ω-B97X-D density functional [5] in Jaguar version 11.2 software [6]. For all stationary points the starting point of the calculations was the crystal structure of **8**, which was modified accordingly. In the reaction mechanism studies stationary point optimization was performed in gas phase using 6-31G* basis set for all atoms excluding Ga, for which we used LANL2DZ basis set and the Gibbs free energy was defined as the sum of the energy obtained from the single-point 6-311++G**/LANL2DZ basis set calculations, zero-point energy, correction to enthalpy, negative product of entropy and temperature (298.15 K) and single-point polarizable continuum model (PCM) solvation energy term for the respective solvent. [7] In the case of the UV-VIS spectrum modelling the optimization was performed using the same method and basis set, but using the PCM dichloromethane model during the optimization.

4.2 Computational results

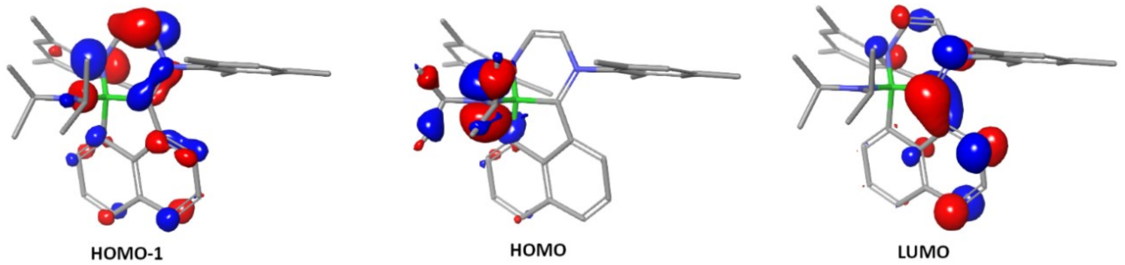

Figure S20: HOMO-1, HOMO and LUMO orbitals of compound **8**.

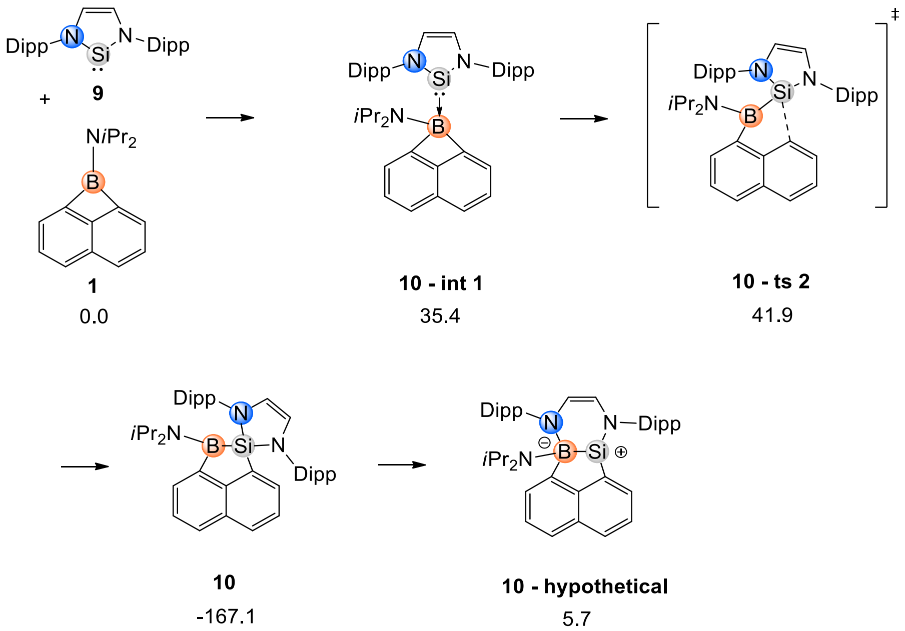

Figure S21: Proposed mechanism for the formation of **10** based on DFT calculations. Values of the standard free enthalpy of intermediates and transition states (ΔG° or ΔG‡) are reported in kJ/mol. Dipp = 2,6-diisopropylphenyl.

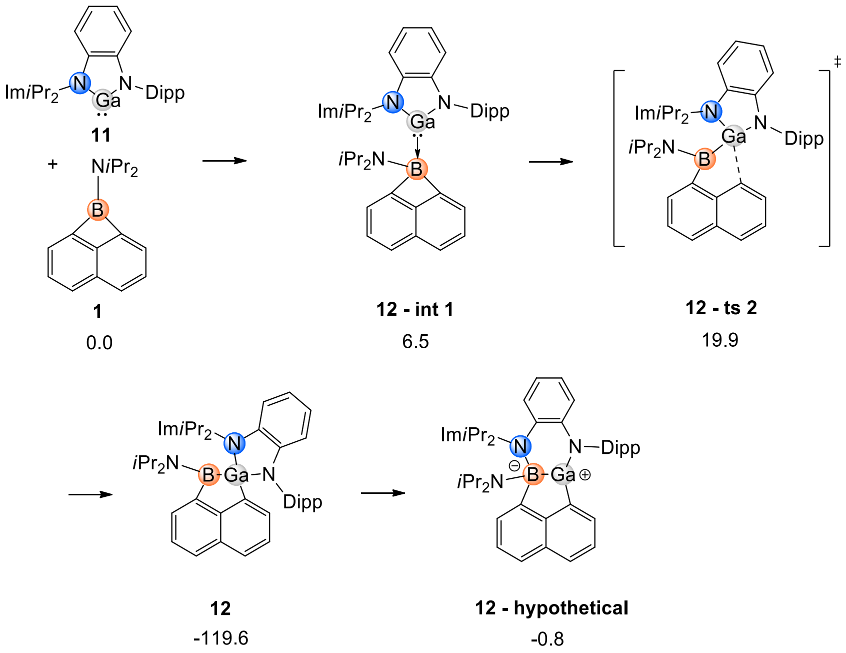

Figure S22: Proposed mechanism for the formation of **12** based on DFT calculations. Values of the standard free enthalpy of intermediates and transition states (ΔG° or ΔG‡) are reported in kJ/mol. Dipp = 2,6-diisopropylphenyl.

Table S2: Total energy values (E) and Gibbs free enthalpy values (G, as defined in the manuscript) for the reaction pathway.

System	E [Hartree]	Zero-point energy [kcal/mol]	Entropy [cal/mol K]	Correction to enthalpy [kcal/mol]	Solvation energy [Hartree]	G [Hartree]
**1 + 7**	-1625.434	462.5	217.3	24.9	-0.012	-1624.773
**8 - int 1**	-1625.459	463.4	211.4	24.3	-0.014	-1624.797
**8 - ts 2**	-1625.419	462.4	210.8	24.2	-0.011	-1624.754
**8 - int 2**	-1625.476	464.7	208.3	24.0	-0.011	-1624.807
**8 - ts 3**	-1625.455	463.1	207.8	23.9	-0.010	-1624.788
**8**	-1625.504	464.1	210.0	24.1	-0.010	-1624.836
**1 + 9**	-2112.755	570.2	241.9	29.0	-0.011	-2111.926
**10 - int1**	-2112.743	570.2	236.2	28.6	-0.012	-2111.913
**10 - ts2**	-2112.739	569.3	236.3	28.5	-0.012	-2111.910
**10**	-2112.824	571.9	232.9	28.3	-0.011	-2111.990
**10 - hypothetical**	-2112.758	570.9	233.2	28.3	-0.010	-2111.924
**1 + 11**	-1973.550	587.1	255.6	31.0	-0.020	-1972.707
**12 - int1**	-1973.545	586.8	253.7	30.8	-0.023	-1972.704
**12 - ts2**	-1973.546	586.8	249.0	30.3	-0.019	-1972.699
**12**	-1973.604	588.4	248.0	30.3	-0.017	-1972.752
**12 - hypothetical**	-1973.560	588.5	244.9	30.0	-0.016	-1972.707

4.3 Cartesian coordinates

Structures **1** and **7**

N3	-1.0046008573	5.3512487251	8.8346665915
N2	-1.3661864718	6.8415764414	7.3800288053
N1	1.4427585367	9.4173429111	9.5166245966
C12	-1.7435792183	4.7356157317	7.8322137036
C2	-0.2304877696	8.4667021923	11.5352178260
C13	-1.9744239883	5.6881357470	6.9020499725
C1	-0.7561282875	6.6695655235	8.5878439128
C3	-1.3660226843	9.3035329865	11.4663823291
C4	-1.1196704896	10.0894806705	10.3203915890
C8	-2.4459700777	9.3744406575	12.3484083639
C24	-0.4312471898	8.4064166264	5.7638271733
C14	-0.6016208473	4.7143466962	10.0500994603
C23	-1.3969832529	8.1130667699	6.7293613835
C21	-1.4271253420	4.8382048291	11.1710462350
C20	-1.0071573874	4.2454340431	12.3594227559
H20	-1.6314890299	4.3445069799	13.2444233154
C11	-0.1741359607	7.5955881752	12.5970079420
H11	0.6403368088	6.8940444562	12.7515164916
C30	-2.3760353667	9.0352937221	7.1069241538
C9	-2.3565504456	8.4540396816	13.4289420711
H9	-3.1355004755	8.4088603061	14.1847007256
C7	-3.3989154293	10.3691145964	11.9962486072
H7	-4.2885401179	10.5218469074	12.6006225856
C27	-1.3832253107	10.6365376995	5.5632286128
C15	0.6042967925	4.0116253421	10.0900777413
C26	-0.4448215775	9.6750581519	5.1891821588
H26	0.3095544480	9.9260378815	4.4467777143
C18	0.2029571629	3.5592010093	12.4481453292
C5	-2.0494803816	11.0591899501	10.0252534167
H5	-1.9556317997	11.7290251378	9.1743431938
C10	-1.2579917081	7.6144183335	13.5251860228
H10	-1.2171551785	6.9232665057	14.3639953470
C29	-2.3469666648	10.2955813957	6.5109545853
H29	-3.0923421733	11.0312985443	6.8041242639
C22	-2.7038775550	5.6304484308	11.0978877445
H22A	-3.3412971560	5.2914070892	10.2751396858
H22B	-2.4761290895	6.6865387476	10.9242257444
H22C	-3.2647688524	5.5555907462	12.0313972565
C17	0.9902532254	3.4441604864	11.3035625460
H17	1.9324627395	2.9032432965	11.3565014418
C6	-3.1846607929	11.1666443120	10.8823725660
H6	-3.9274627224	11.9268450132	10.6518431166
C31	-3.4168037039	8.6703455022	8.1310527911
H31A	-4.0922085337	9.5070196040	8.3188391405
H31B	-2.9431533686	8.4009864437	9.0784433214
H31C	-4.0067296366	7.8095347440	7.8003679481
C32	2.5848606202	8.5633231944	9.8783379179
H32	2.2673143159	8.0830159100	10.8088141748
C25	0.6074113363	7.3834939424	5.3834014874
H25A	1.1325960500	7.0156308246	6.2708794898
H25B	1.3424859645	7.8104938173	4.6967058996
H25C	0.1531221721	6.5137375202	4.8985409002
C33	2.8172744596	7.4250696795	8.8776570705
H33A	3.3287096863	7.7556154139	7.9699171703
H33B	1.8419325672	7.0045037124	8.6147189791
H33C	3.4296910617	6.6425683637	9.3382190182
C19	0.6684580614	2.9926245003	13.7662091790
H19A	1.1925211219	3.7567310555	14.3519072797

H19B	1.3587085711	2.1569343851	13.6226177950
H19C	-0.1739375156	2.6404057863	14.3675506830
C16	1.4572116689	3.8688162387	8.8567902430
H16A	1.6414038677	4.8393262858	8.3898259185
H16B	0.9654309397	3.2401041625	8.1074278115
H16C	2.4202236065	3.4139852584	9.1001397565
C28	-1.3242421446	12.0265326702	4.9806642157
H28A	-0.5693028813	12.6293913145	5.4985692279
H28B	-2.2826844221	12.5429310803	5.0782533303
H28C	-1.0547904570	12.0046149580	3.9206393613
B1	0.2591399119	9.2621053895	10.2246965227
C34	3.8639603089	9.3370329656	10.2052708128
H34A	4.6178577534	8.6459179587	10.5956424371
H34B	3.6719774428	10.0997773805	10.9652655530
H34C	4.2934041565	9.8260403781	9.3262312334
C74	1.8870123907	11.8174169053	9.0368595237
H37A	1.2467291003	12.0764954233	9.8843699018
H37B	1.7807416775	12.5962525156	8.2739245779
H37C	2.9270781534	11.8176063055	9.3764394633
C78	1.4812293010	10.4536091341	8.4657520293
H79	0.4457976525	10.5390659619	8.1163629191
C80	2.3146261918	10.1046032426	7.2334718477
H36A	3.3879439421	10.0797821780	7.4428126091
H36B	2.1453452819	10.8699913343	6.4696473993
H36C	2.0158123296	9.1447343477	6.8133273299
H84	-2.0319961016	3.6976352878	7.8788997947
H85	-2.5075311061	5.6582090841	5.9652141796

Structure **8 - int 1**

N3	-1.3125594402	5.2526852190	8.7676020057
N2	-1.7300013584	6.7143893744	7.2652718783
N1	1.0716106973	8.0545871080	8.7197332116
C12	-2.0530023140	4.6138666608	7.7887847973
C2	-0.4569730679	7.5271315883	10.9710337964
C13	-2.3165190794	5.5371945543	6.8422058833
C1	-1.1019198017	6.5549359291	8.4560390961
C3	-1.3687439367	8.5981352283	10.9657389755
C4	-1.3829143233	9.0055768060	9.6183637492
C8	-2.0042959332	9.1988112894	12.0585438054
C24	-0.7313670073	8.1847431697	5.6086814853
C14	-0.8227343151	4.5368963892	9.9111297169
C23	-1.7905562587	7.9121721064	6.4778212019
C21	-1.6317547199	4.4519761391	11.0472470510
C20	-1.1465481793	3.7218284300	12.1268694724
H20	-1.7487269311	3.6583533290	13.0296891830
C11	-0.1437375736	7.0076462990	12.2064883490
H11	0.5662834987	6.1941149339	12.3399792683
C30	-2.9388308369	8.7013020782	6.5432149798
C9	-1.7001559642	8.5948742106	13.3090323900
H9	-2.1450016657	8.9679934376	14.2282519113
C7	-2.7831897320	10.3388522550	11.7197077541
H7	-3.3287393896	10.8850993351	12.4844147001
C27	-1.9222849524	10.1834217240	4.8989200484
C15	0.4080850580	3.8885389917	9.8106313477
C26	-0.8136157302	9.3392308897	4.8355504255
H26	0.0079886615	9.5832724196	4.1667098645
C18	0.1026863203	3.1011039142	12.0929356508
C5	-2.1067956741	10.1435685425	9.3394479076
H5	-2.1556003515	10.5831967178	8.3458970216
C10	-0.7957233846	7.5474613741	13.3553259791
H10	-0.5599018170	7.1169035240	14.3264601021
C29	-2.9793652929	9.8406653484	5.7407968682
H29	-3.8563207339	10.4818122971	5.7853812356
C22	-2.9538865038	5.1652270365	11.1154763012
H22A	-3.5740147929	4.9581996631	10.2375175829
H22B	-2.7935355579	6.2469972326	11.1647213199
H22C	-3.5074756689	4.8640993975	12.0063191005
C17	0.8606190910	3.1858229458	10.9273424806
H17	1.8266450538	2.6891172011	10.8787256757
C6	-2.8100082262	10.7779715211	10.4080757093
H6	-3.3908566176	11.6689604259	10.1785841330
C31	-4.0884626047	8.3321815742	7.4430394616
H31A	-4.7988394434	9.1578960284	7.5172290060
H31B	-3.7359649337	8.1043358490	8.4518545323
H31C	-4.6242540576	7.4562742177	7.0611796838
C32	2.1566090487	7.2562319325	9.3034866773
H32	1.6518234411	6.3765465642	9.7227502870
C25	0.4430550751	7.2510462088	5.5278316327
H25A	0.9270445961	7.2048968882	6.5107226675
H25B	1.1686003699	7.5968885548	4.7884666289
H25C	0.1265809512	6.2392974638	5.2504239348
C33	3.1773906889	6.7108486039	8.2878083506
H33A	3.8704383319	7.4810359125	7.9412094273
H33B	2.6843765386	6.2871015234	7.4084511425
H33C	3.7785239175	5.9206820368	8.7529390553
C19	0.6297562280	2.3823165152	13.3088513952
H19A	1.0848541072	3.0943930212	14.0062601646
H19B	1.3929674783	1.6471141753	13.0411452398
H19C	-0.1714400071	1.8653440570	13.8444738535
C16	1.1994673025	3.8924126701	8.5284555790

H16A	1.0719672035	4.8213280242	7.9699234212
H16B	0.8811190628	3.0682265324	7.8795352713
H16C	2.2655870663	3.7668814397	8.7296020093
C28	-1.9627773170	11.4532918193	4.0874847409
H28A	-1.4649574345	12.2668085541	4.6258760819
H28B	-2.9900738675	11.7689201584	3.8881526610
H28C	-1.4495596194	11.3310698306	3.1297036607
B1	-0.3004323116	7.7514784337	9.3092546984
C34	2.9112778824	7.8973482481	10.4811411021
H34A	3.5226306374	7.1415702095	10.9874935838
H34B	2.2142420568	8.3180344361	11.2086218696
H34C	3.5873827375	8.6887958211	10.1417007013
C74	1.4529856090	10.4909811724	9.4278935519
H37A	0.7448096315	10.3131618731	10.2376558105
H37B	1.2596840662	11.4931699207	9.0282258651
H37C	2.4623153826	10.4951468959	9.8487640400
C78	1.2969720959	9.4451950821	8.3008769304
H79	0.3790642187	9.7124259027	7.7655739835
C80	2.4432823529	9.6354702044	7.2989762230
H36A	3.4225333265	9.5548653613	7.7825084621
H36B	2.3825559687	10.6401777078	6.8665791626
H36C	2.4035857595	8.9097288728	6.4832735236
H84	-2.3118786313	3.5715373884	7.8717299313
H85	-2.8580582015	5.4816801231	5.9125740178

Structure **8 - ts 2**

N3	-1.0675533301	5.5096496384	9.8216490164
N2	-2.0706063829	6.9599237614	8.5092240112
N1	1.4881058312	7.7896337511	9.9761543535
C12	-2.3047389928	4.9708075653	9.4367282834
C2	-1.5358029774	8.1512019955	10.9088495961
C13	-2.9152170080	5.8579971813	8.6450173390
C1	-0.9922361906	6.8421154566	9.4006778755
C3	-1.4475142508	9.4225037422	10.2995143941
C4	-0.4709314346	9.4260774630	9.2774277380
C8	-2.2264231100	10.5365381724	10.6643914198
C24	-1.5191999041	8.2465298105	6.4990866349
C14	-0.0538440125	4.6150292363	10.2893648646
C23	-2.3603352471	8.0216434098	7.6002371191
C21	-0.1204382555	4.1371611655	11.6054177659
C20	0.8241321590	3.2014981193	12.0232770949
H20	0.7812995976	2.8355848972	13.0465411410
C11	-2.4214679909	7.9965576010	11.9480517875
H11	-2.5710537268	7.0430088167	12.4455793136
C30	-3.5128164259	8.7973094772	7.7897526258
C9	-3.1292108911	10.3413986223	11.7444057892
H9	-3.7604184580	11.1608817665	12.0771308302
C7	-2.0134899050	11.7190692704	9.9081912851
H7	-2.5814502285	12.6176622514	10.1332468678
C27	-2.9538051706	10.0808178942	5.7941120957
C15	0.9238169518	4.1433535872	9.4034206478
C26	-1.8304139764	9.2761413797	5.6183651328
H26	-1.1733931195	9.4560099847	4.7701391886
C18	1.8228860470	2.7337505493	11.1725865526
C5	-0.2882918795	10.5910425073	8.5733664139
H5	0.4291617124	10.6684469255	7.7598081153
C10	-3.2035636752	9.1129349714	12.3640904990
H10	-3.9024596759	8.9810025400	13.1867590191
C29	-3.7841904134	9.8159659123	6.8769896754
H29	-4.6687629656	10.4297656298	7.0323706719
C22	-1.1399960950	4.6690925560	12.5756626410
H22A	-1.1277344993	4.0971543573	13.5063071540
H22B	-2.1537565865	4.6428560544	12.1666562132
H22C	-0.9172993698	5.7143821042	12.8137129424
C17	1.8509394954	3.2126292787	9.8652978234
H17	2.6148061822	2.8502041364	9.1810440841
C6	-1.0802745253	11.7262794154	8.8939620130
H6	-0.9347182755	12.6379935048	8.3201811393
C31	-4.4666777299	8.5650916413	8.9325761424
H31A	-4.9101785953	9.5109976444	9.2522569547
H31B	-3.9717432922	8.1191242673	9.7946017923
H31C	-5.2848353905	7.9004228235	8.6294730343
C32	1.7690359347	7.0710617220	11.2257503307
H32	0.9867281839	6.3199203197	11.2926921464
C25	-0.2821962856	7.4246370414	6.2728029108
H25A	0.5072623187	7.6905444810	6.9872898780
H25B	0.1138644254	7.5880069664	5.2677034562
H25C	-0.4871088025	6.3597128738	6.3958792302
C33	3.0915950707	6.3052287174	11.2482185479
H33A	3.9537875753	6.9748385034	11.3333726964
H33B	3.2046640976	5.6941671781	10.3504660318
H33C	3.1049733008	5.6373640404	12.1154635710
C19	2.8679122730	1.7629161945	11.6596357770
H19A	3.7636079790	2.2982812065	11.9947004252
H19B	3.1741554267	1.0755519039	10.8659767013
H19C	2.5022478324	1.1705272164	12.5024727810
C16	0.9798495666	4.6392167237	7.9875788225

H16A	1.2709664599	5.6931411186	7.9705455717
H16B	-0.0002709396	4.5560809383	7.5078930297
H16C	1.7040873421	4.0706248318	7.3993609381
C28	-3.2437687751	11.2249698064	4.8568416965
H28A	-2.8315672210	12.1595728652	5.2531866314
H28B	-4.3196341771	11.3707987910	4.7241338530
H28C	-2.7981265274	11.0582569846	3.8722256030
B1	0.1142536470	7.9561907874	9.5562118208
C34	1.6097564766	7.9272874664	12.4946508634
H34A	1.5659263292	7.2691134754	13.3703464512
H34B	0.6790494120	8.4980113763	12.4546437839
H34C	2.4406670197	8.6199267535	12.6459823687
C74	3.0672305025	9.7558489182	10.3368214151
H37A	2.2818498653	10.2810373389	10.8860246856
H37B	3.6328134035	10.4934971876	9.7580073180
H37C	3.7593248210	9.3030680217	11.0533318698
C78	2.4598779251	8.7211882885	9.3859806443
H79	1.8870168956	9.2928934209	8.6522518459
C80	3.5499928342	8.0043312254	8.5742618361
H36A	4.2826742920	7.4990426190	9.2073622304
H36B	4.0912034425	8.7298365270	7.9570422069
H36C	3.1021145679	7.2577234081	7.9113400417
H84	-2.5962474631	3.9811030765	9.7481733599
H85	-3.8643154242	5.8158202274	8.1370562566

Structure **8 - int 2**

N3	0.1165857364	5.4734141735	8.9345868551
N2	-0.6339672663	7.0085406019	7.3148753848
N1	2.4197576086	7.8016324993	8.0183041101
C12	-0.1301085918	4.8367588200	7.6974265901
C2	-1.0207425964	7.5992855043	9.7280218455
C13	-0.5667609206	5.7085399361	6.7881267589
C1	-0.0769134182	6.9435559344	8.7357291108
C3	-0.5763761968	8.9051478691	10.0424195286
C4	0.7026837007	9.2510972136	9.5127229131
C8	-1.3365462476	9.7629189413	10.8673158248
C24	-1.2920443933	9.2098670855	6.4661776220
C14	-0.0489496803	4.8086902444	10.1810229591
C23	-1.6588987627	7.9311666640	6.9260566285
C21	-1.0009540697	3.7843267076	10.3806460900
C20	-1.0838020990	3.1676640558	11.6310546735
H20	-1.8263960194	2.3835681788	11.7665362282
C11	-2.1824862267	7.1306053280	10.2845171680
H11	-2.5160279091	6.1142570567	10.1120428030
C30	-3.0288873719	7.5886361760	6.9635651666
C9	-2.5604561439	9.2677417970	11.3851608229
H9	-3.1683172662	9.9073534212	12.0181452565
C7	-0.7978165844	11.0441881604	11.1426679714
H7	-1.3582437733	11.7391577866	11.7611656945
C27	-3.6375518786	9.8386498758	6.2461869655
C15	0.7942599167	5.1653881195	11.2570647015
C26	-2.2863805279	10.1397325892	6.1538755087
H26	-1.9820282250	11.1272655282	5.8125116984
C18	-0.2870454990	3.5332400727	12.7072752064
C5	1.1955400482	10.4964858673	9.8548476583
H5	2.1785704422	10.8265188418	9.5368000534
C10	-2.9545338935	7.9810074637	11.1121258476
H10	-3.8781748837	7.6009141966	11.5375897309
C29	-3.9832378458	8.5445220022	6.6288676996
H29	-5.0347892465	8.2687189708	6.6754265643
C22	-1.9750579727	3.2999996325	9.3298939525
H22A	-1.5568354223	2.4784378494	8.7380333602
H22B	-2.2617727498	4.0776771718	8.6259427165
H22C	-2.8812455479	2.9234285477	9.8121524745
C17	0.6491840781	4.5391388469	12.4904550650
H17	1.3060629807	4.8389476477	13.3044598194
C6	0.4367763410	11.3895102324	10.6498169615
H6	0.8526578134	12.3655079420	10.8824327827
C31	-3.5053088764	6.2044126393	7.3141248088
H31A	-4.5556574565	6.2238191933	7.6156808911
H31B	-2.9249355191	5.7799757282	8.1283710024
H31C	-3.4115128462	5.5223255390	6.4620948609
C32	2.7888948504	6.4854805253	7.4584077568
H32	2.0744307332	5.7950388817	7.9031302528
C25	0.1379926788	9.6272911598	6.2393297034
H25A	0.8002543950	8.7654335262	6.1819856412
H25B	0.4945658364	10.2719052952	7.0486517605
H25C	0.2146413646	10.1955276725	5.3078927800
C33	2.6073306469	6.3683494950	5.9382147210
H33A	3.4275289337	6.8169724810	5.3731670120
H33B	1.6680368017	6.8328561486	5.6305175462
H33C	2.5561652183	5.3090009147	5.6666576653
C19	-0.4439492654	2.8916289250	14.0618175276
H19A	-1.0668844989	3.5101685192	14.7175303177
H19B	0.5234695809	2.7647331084	14.5567993566
H19C	-0.9180959517	1.9093880753	13.9864712594
C16	1.8754090037	6.1989697745	11.1031586091

H16A	1.4753529070	7.2171895639	11.1480161769
H16B	2.3907387003	6.0863004152	10.1482484162
H16C	2.6137058154	6.1073436698	11.9039806643
C28	-4.6968228731	10.8683788274	5.9493366388
H28A	-5.0715913459	11.3169016408	6.8758737022
H28B	-5.5521052736	10.4247207800	5.4310461540
H28C	-4.3032762018	11.6765073675	5.3276898451
B1	1.2093726046	7.9728093990	8.7069659247
C34	4.1723999048	5.9900198289	7.8916329407
H34A	4.2884801055	4.9475608139	7.5797591445
H34B	4.2885562747	6.0313005309	8.9782085230
H34C	4.9892925765	6.5578557532	7.4378115028
C74	4.3256620201	9.0509964575	9.0488366414
H37A	3.8068415129	8.9349038786	10.0043154632
H37B	4.8229224812	10.0271356707	9.0440209986
H37C	5.0989569629	8.2818705426	8.9825213673
C78	3.3248572775	8.9601674197	7.8912402594
H79	2.6701226089	9.8290886409	7.9610003459
C80	4.0138683829	9.1121196713	6.5335474729
H36A	4.7667732846	8.3434112870	6.3440441539
H36B	4.5251693691	10.0796550205	6.5127341802
H36C	3.2862658474	9.0991158233	5.7185310388
H84	0.1171564740	3.7954257470	7.5673433355
H85	-0.7822746669	5.5392053872	5.7437795079

Structure **8 - ts 3**

N3	0.1129225748	5.5394330025	9.0975004016
N2	-0.4070941684	7.4299411699	7.5570802608
N1	2.3201651068	7.8307820259	7.8777219619
C12	0.1235411785	5.1843230702	7.7501085412
H12	0.3735027355	4.1712202763	7.4706327441
C2	-0.6821122543	7.6338906223	10.2979937910
C13	-0.2236263675	6.1838545289	6.9276009677
H13	-0.3774033371	6.0996298314	5.8603293879
C1	0.0841859610	6.9418653868	9.2316675205
C3	-0.4450669066	9.0272452439	10.1741317528
C4	0.4564599613	9.3843729311	9.1308742071
C8	-1.0916972440	9.9595146726	11.0113867615
C24	-1.2796731792	9.2313746216	6.1663674646
C14	-0.2027975312	4.5930117380	10.1239793603
C23	-1.5273977336	8.2148230135	7.1065783856
C21	-1.2478704818	3.6614720976	9.9960582281
C20	-1.4653396526	2.7627518180	11.0452366866
H20	-2.2732990762	2.0408513516	10.9412517089
C11	-1.5393581462	7.1709390580	11.2714780968
H11	-1.7515117063	6.1144988993	11.3852127814
C30	-2.8356102039	7.9595947296	7.5403433005
C9	-1.9572679958	9.4532086507	12.0148455511
H9	-2.4564640995	10.1469518020	12.6862308792
C7	-0.8214124214	11.3339063951	10.7884889536
H7	-1.2981988294	12.0845436478	11.4127335512
C27	-3.6429468872	9.8144779223	6.1781585180
C15	0.6082256692	4.5946763646	11.2796454669
C26	-2.3421902560	10.0093723854	5.7193117102
H26	-2.1465162432	10.7870203025	4.9836638487
C18	-0.7099575723	2.7697023129	12.2087534656
C5	0.6763452051	10.7336962243	8.9596860944
H5	1.3513294619	11.1028978037	8.1935046698
C10	-2.1625933013	8.0990548357	12.1387397462
H10	-2.8252978740	7.7251793105	12.9133856977
C29	-3.8658881456	8.7794409448	7.0774859634
H29	-4.8783535112	8.5836662328	7.4255046779
C22	-2.1955695917	3.5466700082	8.8203322343
H22A	-1.9786342385	4.2318148758	8.0055102784
H22B	-3.2212751795	3.7364246324	9.1528768693
H22C	-2.1674473649	2.5279749787	8.4192875301
C17	0.3276889150	3.6969227942	12.3024683799
H17	0.9514585645	3.7087166691	13.1931463442
C6	0.0391240888	11.6972319032	9.7837040308
H6	0.2454773044	12.7496717350	9.6101782084
C31	-3.2110458008	6.8176699697	8.4485969925
H31A	-3.6768992141	7.1902628331	9.3646038888
H31B	-2.3556768144	6.2158604267	8.7367467307
H31C	-3.9317008338	6.1634230994	7.9459672415
C32	3.0193643778	6.5482563188	7.9938544698
H32	2.3344101291	5.9104462196	8.5499216193
C25	0.0912166209	9.4824204929	5.5950590833
H25A	0.7562791670	8.6255238896	5.7137081761
H25B	0.5580600416	10.3364608568	6.0962386657
H25C	0.0201378351	9.7272865367	4.5313367581
C33	3.2744410389	5.8140888987	6.6627422070
H33A	4.1532782779	6.1878196969	6.1315169922
H33B	2.4070517882	5.9082297473	6.0060539262
H33C	3.4415433325	4.7478176767	6.8561327231
C19	-1.0116265219	1.8181431399	13.3385960181
H19A	-1.7827112071	2.2293742276	13.9999974189
H19B	-0.1232378007	1.6277982919	13.9468008701

H19C	-1.3791875505	0.8587539606	12.9635347690
C16	1.7774559016	5.5350881625	11.4221797529
H16A	1.4600488959	6.5798122609	11.4925572099
H16B	2.4494888200	5.4583142571	10.5643121373
H16C	2.3481846044	5.2977116946	12.3227780215
C28	-4.7681958644	10.7010845123	5.7097616391
H28A	-4.8117549543	11.6196936830	6.3049201332
H28B	-5.7359066925	10.2007516235	5.8026868157
H28C	-4.6347006613	10.9939207729	4.6641386423
B1	0.9744938030	8.0066770098	8.4364490551
C34	4.3098819263	6.5850197454	8.8260054186
H34A	4.6433891843	5.5614450669	9.0323086782
H34B	4.1455701765	7.0932463415	9.7805542779
H34C	5.1276507228	7.0896501358	8.3023385577
C76	3.8772460664	9.7222036661	8.5161494753
H37A	3.3084813844	9.7859474678	9.4466628831
H37B	4.1291974293	10.7413956227	8.1998095982
H37C	4.8160680327	9.2015054339	8.7196888615
C80	3.0429599715	9.0233984459	7.4262279244
H81	2.2648081320	9.7315331109	7.1434743680
C82	3.8792425292	8.8366848593	6.1550527315
H36A	4.7787736208	8.2397962187	6.3325788044
H36B	4.2089418694	9.8160166231	5.7925769592
H36C	3.2966326407	8.3565011163	5.3638437924

Structure **8**

N3	-0.0824964705	5.4685470889	9.2700986942
N2	-0.6071939029	7.3227779161	7.2119854685
N1	1.7438416646	8.1411150583	8.0257532371
C12	-0.3165369716	5.0627607221	7.9439598268
H12	-0.3440303512	3.9984198095	7.7707291246
C2	-0.0156946020	7.3669448357	10.8549554833
C13	-0.6578815603	5.9944936134	7.0054519146
H13	-1.0247987287	5.6460198303	6.0416005936
C1	0.1271702872	6.7386927339	9.5425946464
C3	-0.3825011978	8.7240585161	10.6073867505
C4	-0.3865806686	9.1088847614	9.2363824071
C8	-0.7346993555	9.5915392969	11.6607952410
C24	-0.8233735287	8.7598863762	5.2244908651
C14	-0.2654828887	4.4642556500	10.2929140188
C23	-1.3858314793	8.1781739224	6.3692503364
C21	-1.5386151738	4.2899114095	10.8459445887
C20	-1.6803043289	3.3443961225	11.8568814374
H20	-2.6596099013	3.2009555250	12.3075442887
C11	0.0734793510	6.9052133965	12.1537803055
H11	0.3650156944	5.8850663212	12.3767731552
C30	-2.7103117039	8.4597376109	6.7357036792
C9	-0.6302285741	9.0869303299	12.9819122423
H9	-0.8740728844	9.7359919020	13.8185544866
C7	-1.1692416128	10.8965343935	11.3103310729
H7	-1.4549020896	11.5943374771	12.0920831890
C27	-2.9043679631	9.9669496903	4.8342557811
C15	0.8381583335	3.7189342428	10.7054334669
C26	-1.5956321740	9.6477396070	4.4773881459
H26	-1.1581148640	10.1050014934	3.5921441251
C18	-0.6006786670	2.5852234433	12.3115149700
C5	-0.8418300953	10.3741604534	8.9491747684
H5	-0.8960431691	10.7189802077	7.9191589737
C10	-0.2225429147	7.7921440526	13.2132423448
H10	-0.1404264038	7.4329442819	14.2345488943
C29	-3.4461434869	9.3550772971	5.9613439683
H29	-4.4690803900	9.5836766658	6.2526481818
C22	-2.6990061161	5.1326857047	10.3926339568
H22A	-2.7777754386	5.1366482509	9.3020570108
H22B	-2.5687231659	6.1715851579	10.7148383727
H22C	-3.6372914466	4.7610708666	10.8095879120
C17	0.6448570178	2.7803565873	11.7204555890
H17	1.4951605620	2.1939500314	12.0597871197
C6	-1.2372798554	11.2584736686	9.9874973604
H6	-1.5876724103	12.2532000893	9.7259164218
C31	-3.3330127121	7.8071994197	7.9411131570
H31A	-4.3288106358	8.2136051857	8.1334644024
H31B	-2.7197210631	7.9633400467	8.8319443516
H31C	-3.4307088939	6.7263422506	7.7948636674
C32	2.6735547476	7.0123349655	8.0532660681
H32	2.0675596220	6.1521141079	8.3438989526
C25	0.5740700563	8.4076449479	4.7926439986
H25A	1.2227245424	8.2999769943	5.6631242301
H25B	0.9832101892	9.1704410716	4.1247645270
H25C	0.5852214510	7.4522820631	4.2531965152
C33	3.2659862328	6.6037863866	6.6920111564
H33A	3.9834515930	7.3287793870	6.3015483531
H33B	2.4676362615	6.4779220716	5.9561627914
H33C	3.7884317118	5.6442267920	6.7896141554
C19	-0.7899128123	1.5857020481	13.4247039569
H19A	-1.0713895863	2.0886935637	14.3557250534
H19B	0.1243952166	1.0178850884	13.6134610466

H19C	-1.5870014439	0.8751836836	13.1845219616
C16	2.2019416829	3.9450964999	10.1081231187
H16A	2.5850544305	4.9329081684	10.3810300704
H16B	2.1760383143	3.9018296945	9.0148706948
H16C	2.9112062595	3.1954763348	10.4656416322
C28	-3.7017122747	10.9587609200	4.0246213655
H28A	-3.2939582385	11.9697054603	4.1319817181
H28B	-4.7468048051	10.9857281202	4.3441783597
H28C	-3.6802265197	10.7103783068	2.9587932691
B1	0.2844967410	7.8862410882	8.3895218526
C34	3.7787289466	7.1068112777	9.1178064800
H34A	4.3118660787	6.1518970593	9.1966178610
H34B	3.3484076190	7.3489842251	10.0952482384
H34C	4.5227882296	7.8713630723	8.8759496098
C76	2.6718753847	10.1267109244	9.3165092176
H37A	1.9514901496	9.8957676933	10.1032085639
H37B	2.7410299135	11.2174337964	9.2335997035
H37C	3.6516847626	9.7581620802	9.6331059251
C80	2.2369129431	9.5210589783	7.9644491113
H81	1.3789472440	10.1134466390	7.6301000958
C82	3.3379787371	9.7851814764	6.9280067223
H36A	4.2889037648	9.3197118554	7.2054423245
H36B	3.5160395240	10.8638522666	6.8644016617
H36C	3.0589161624	9.4304336463	5.9331133667

Structures **1** and **9**

N3	-1.0113526046	4.9006001423	9.1455010953
N2	-1.5878860237	6.7120801690	7.6488132538
N1	1.4787102169	10.2575445117	10.0428130386
C12	-1.9561961291	4.5286312416	8.1964661409
C2	-0.9433814009	9.5412601661	11.2596307743
C13	-2.2695808294	5.5357164290	7.3538976552
Si7	-0.4820164358	6.5802442579	9.0164403125
C3	-1.7416988667	10.6994954704	11.1449316612
C4	-0.9311192893	11.5794747701	10.3955304123
C8	-3.0140635146	10.9313220163	11.6706085689
C24	-0.6877573049	7.6498445743	5.5944950520
C14	-0.3779062406	3.9130242761	9.9626902416
C23	-1.5685221460	7.7694175175	6.6853748664
C21	-0.7367525814	3.7926377484	11.3166370480
C20	-0.0747542479	2.8361843486	12.0866758246
H20	-0.3320594206	2.7181046566	13.1342276523
C11	-1.4840088531	8.4909422961	11.9670733922
H11	-0.9562573709	7.5564414812	12.1370441392
C30	-2.4007849562	8.8928806884	6.8493466621
C9	-3.5498748144	9.8158262245	12.3691531262
H9	-4.5414097535	9.8617226208	12.8098210315
C7	-3.5139141245	12.2326072398	11.3918051453
H7	-4.4951560079	12.5359580887	11.7448852299
C27	-1.5336512493	9.7521801801	4.7510476055
C15	0.6241702149	3.1083801892	9.3880419225
C26	-0.6987090133	8.6533361238	4.6253203454
H26	-0.0384834333	8.5752650455	3.7657883278
C18	0.9147563399	2.0325805811	11.5368296790
C5	-1.4461258538	12.8310897969	10.1471042906
H5	-0.9114243871	13.5946880181	9.5884631885
C10	-2.7972386812	8.6581737941	12.4969852809
H10	-3.2352953525	7.8248303145	13.0412370244
C29	-2.3631999832	9.8742233193	5.8588651080
H29	-2.9882826442	10.7547754405	5.9528345563
C22	-1.8468265170	4.6459480373	11.9116676153
H36	-1.8733156752	5.5881887260	11.3566061105
C37	1.2608001247	2.1707465264	10.2003416087
H38	2.0453259459	1.5455298679	9.7843387434
C39	-2.7448307072	13.1243689571	10.6613376221
H40	-3.1576784106	14.1111078010	10.4673265219
C41	-3.3156655623	9.0294413053	8.0616453179
H42	-2.7409279341	8.7216227107	8.9434379816
C43	2.2312873483	9.0537851047	10.4470805395
H44	1.4700223080	8.4191702730	10.9104271960
C45	0.2758983936	6.4786651392	5.4524720989
H46	0.2308111471	5.8890174563	6.3712850661
C47	2.8177087990	8.2333075026	9.2898753264
H48	3.7621107381	8.6367708362	8.9176948232
H49	2.1070828847	8.1685332870	8.4627808959
H50	3.0101763760	7.2141304438	9.6415154604
H51	1.4223025101	1.2986744077	12.1556053182
C52	1.0594681396	3.2844674596	7.9383377326
H53	0.2844013152	3.8521765446	7.4171844648
H54	-1.5301131014	10.5274532304	3.9906019431
B55	0.1521362904	10.3871600301	10.4350592544
C56	3.2974630284	9.3436013509	11.5079239398
H57	3.6935370797	8.4011145359	11.8992437886
H58	2.8758737622	9.9139773291	12.3404238728
H59	4.1401922029	9.9053230873	11.0916303819
C60	2.6250847629	12.4383125306	10.4828751488
H61	1.8391975479	12.6706383057	11.2066654055

H62	2.9323975311	13.3717848915	9.9996734547
H63	3.4880718824	12.0414501595	11.0238343925
C64	2.1036473060	11.4513820630	9.4307776950
H65	1.2800263737	11.9500760023	8.9108407913
C66	3.1678877971	11.1584162872	8.3750438572
H67	4.0895208274	10.7665650118	8.8153197893
H68	3.4226826294	12.0933071558	7.8669433379
H69	2.8064489039	10.4518941452	7.6243917870
H70	-2.3479684790	3.5201899650	8.1797178075
H71	-2.9622234864	5.4976392033	6.5239106472
C72	1.7263468444	6.9565492354	5.3028664059
H73	2.4090649525	6.1005842937	5.2863777725
H74	1.8780788015	7.5173614927	4.3744807945
H75	2.0144374337	7.6030980156	6.1376824616
C76	-0.1296347920	5.5491897009	4.3019892014
H77	0.5522588910	4.6937309959	4.2425701924
H78	-1.1437190283	5.1658919841	4.4491167585
H79	-0.1003749859	6.0719182956	3.3394353990
C80	-3.7877380543	10.4634435918	8.3063224624
H81	-4.3322845804	10.5101858077	9.2517725798
H82	-2.9512635888	11.1647311052	8.3724570723
H83	-4.4708382450	10.8053058219	7.5198519753
C84	-4.5349152673	8.1002538732	7.9554032006
H85	-5.1773262347	8.2256746067	8.8330307156
H86	-5.1244662454	8.3426551800	7.0638313635
H87	-4.2439201681	7.0499556179	7.9018701318
C88	2.3596907165	4.0995562801	7.8623887824
H89	2.6641554697	4.2422485506	6.8194843639
H90	2.2340449289	5.0880602896	8.3167969547
H91	3.1738756297	3.5865757497	8.3869849564
C92	1.2033424482	1.9512468586	7.1956697115
H93	1.3998202373	2.1347785753	6.1343550814
H94	2.0351199084	1.3517703268	7.5807722667
H95	0.2907219428	1.3531689501	7.2750424436
C96	-3.2079287727	3.9663223118	11.7051944376
H97	-4.0140725134	4.5882743517	12.1080223294
H98	-3.4053986894	3.8020129027	10.6420989796
H99	-3.2362494167	2.9955159393	12.2126517601
C100	-1.6212362684	4.9900743531	13.3861209325
H101	-2.3609737921	5.7258434044	13.7166316078
H102	-1.7283119912	4.1144263783	14.0353289064
H103	-0.6253430788	5.4129008731	13.5490171003

Structure **10 - int 1**

N3	-1.5061130600	4.8111569543	8.4547957408
N2	-2.1034819907	6.5300602852	6.8191624328
N1	1.3106837379	7.8102467922	9.0721240644
C12	-2.3116708418	4.3317236119	7.4213428195
C2	-0.5758848873	7.1368063797	11.0246357906
C13	-2.6440631248	5.2772741528	6.5220595902
Si7	-1.1639074340	6.4879667673	8.2664623064
C3	-1.3667642539	8.2929060327	11.1626572793
C4	-1.1208049159	8.9994336105	9.9700373849
C8	-2.1703485696	8.6658582882	12.2414877818
C24	-1.4084905125	7.5421586640	4.7158137669
C14	-1.0811146258	3.9269160356	9.4971543819
C23	-2.2070218183	7.5988326827	5.8710996583
C21	-2.0504505957	3.4173788468	10.3714691710
C20	-1.6372413758	2.5125177248	11.3506372380
H20	-2.3695638159	2.0986538054	12.0380524409
C11	-0.5907069643	6.2493080715	12.0772806446
H11	-0.0303051631	5.3178980671	12.0793821131
C30	-3.0976509138	8.6604221545	6.1213920817
C9	-2.1607458288	7.7300119866	13.3132597960
H9	-2.7406174196	7.9124988405	14.2143614201
C7	-2.8106372378	9.9254700291	12.0714975257
H7	-3.4594578646	10.3264893117	12.8458647126
C27	-2.4378319063	9.6068547838	3.9880583195
C15	0.2862029422	3.6022420659	9.6115586384
C26	-1.5533093462	8.5616441742	3.7741058181
H26	-0.9584400496	8.5396156828	2.8659176779
C18	-0.3063205795	2.1484198231	11.4643135441
C5	-1.7390750817	10.2226304312	9.8488272937
H5	-1.6080689973	10.8686922183	8.9827821119
C10	-1.3918955981	6.5817076327	13.2128898463
H10	-1.4044343379	5.8834271679	14.0466172033
C29	-3.1921755911	9.6593231815	5.1538120832
H29	-3.8646181481	10.4952882871	5.3103017934
C22	-3.5123349043	3.8347312882	10.3047713568
H36	-3.6221578644	4.5842204338	9.5172692745
C37	0.6437968219	2.6977867647	10.6100748799
H38	1.6826560565	2.4145650012	10.7330769474
C39	-2.5849108229	10.6560542950	10.9170640814
H40	-3.0779456848	11.6204732938	10.8171599621
C41	-3.9368769159	8.7059784917	7.3935482770
H42	-3.2859400104	8.4254023588	8.2285934530
C43	2.2054195299	8.3599932576	10.1061650992
H44	1.6771589377	8.1565977729	11.0429771602
C45	-0.4129032142	6.4163985135	4.4711771179
H46	-0.2703165850	5.8806647535	5.4145816623
C47	3.5489546534	7.6291545538	10.2422805700
H48	4.2717304803	7.9070901855	9.4713729936
H49	3.3946766641	6.5490040981	10.1970265597
H50	3.9998336511	7.8711623819	11.2108524152
H51	0.0020064549	1.4432335171	12.2302227999
C52	1.3405087580	4.2371490228	8.7051472236
H53	1.1928208033	5.3258974884	8.7348879791
H54	-2.5347734871	10.3946285008	3.2472967718
B55	-0.1069731456	7.7337608747	9.5275279806
C56	2.4069902342	9.8815125180	10.0555740210
H57	2.9316228421	10.2192075170	10.9558259993
H58	1.4431076914	10.3957464230	10.0086731834
H59	3.0096008041	10.1872857440	9.1934086352
C60	0.9119925610	9.4116136350	7.1409584753
H61	-0.1470858528	9.4815486267	7.4069356392

H62	0.9808722520	9.4742946094	6.0490647444
H63	1.4234147645	10.2806454800	7.5626741201
C64	1.5241087479	8.0925566057	7.6511251486
H65	0.9754851510	7.2896931595	7.1182303017
C66	2.9740837093	7.9504265368	7.1868435197
H67	3.6089325171	8.7570149598	7.5642151148
H68	3.0164138541	7.9907149783	6.0942974787
H69	3.3950235415	6.9943778465	7.5099612392
H70	-2.6077596671	3.2927726042	7.4109501182
H71	-3.2635390245	5.1550104163	5.6459837891
C72	0.9623309807	6.9385692501	4.0377813037
H73	1.6922547258	6.1227456726	4.0453652665
H74	0.9417202527	7.3493627323	3.0230137145
H75	1.3185033871	7.7215987953	4.7104916673
C76	-0.9477817914	5.4121656363	3.4405210438
H77	-0.2308484397	4.5983376911	3.2908959639
H78	-1.8959441878	4.9725439432	3.7594745411
H79	-1.1113632801	5.9020217442	2.4745300765
C80	-4.4772662081	10.0989338105	7.7250548708
H81	-4.8992986329	10.0935242224	8.7338887664
H82	-3.6875739732	10.8538103509	7.7027845263
H83	-5.2704463543	10.4039727866	7.0327856755
C84	-5.0940579763	7.6967952755	7.3348214289
H85	-5.6788312836	7.7410243679	8.2588507469
H86	-5.7616914422	7.9286510039	6.4975952991
H87	-4.7364160093	6.6720475078	7.2124891094
C88	2.7740710819	4.0185189381	9.1953339096
H89	3.4607693699	4.6130903572	8.5854760140
H90	2.8984454528	4.3301273786	10.2363130333
H91	3.0823780184	2.9700701211	9.1083424107
C92	1.2262531184	3.7634424933	7.2465695929
H93	1.9452004487	4.3040033722	6.6209774627
H94	1.4533011602	2.6941886920	7.1741627954
H95	0.2293864142	3.9214467246	6.8281317494
C96	-4.4107122350	2.6451439176	9.9414497514
H97	-5.4527060034	2.9660376069	9.8466913711
H98	-4.1075986957	2.1876898421	8.9942071940
H99	-4.3686045512	1.8678052260	10.7119903769
C100	-3.9624916570	4.4976342652	11.6126065903
H101	-5.0015047651	4.8323881285	11.5271561485
H102	-3.9061201168	3.8017545438	12.4567196633
H103	-3.3388564559	5.3634107274	11.8481106603

Structure **10 - ts 2**

N3	-1.0633901767	5.4063046680	9.8388945827
N2	-2.1739961995	7.0481225914	8.3441772513
N1	1.6565799907	8.1014894820	10.5844464874
C12	-2.1925666994	4.8957848052	9.1688541099
C2	-1.1341637798	8.8489244185	11.1128524384
C13	-2.7835579428	5.7825656131	8.3500384248
Si7	-0.8169342386	7.0568146666	9.4024866795
C3	-1.1982951793	10.0147316638	10.3193200631
C4	-0.1318066540	9.8997433200	9.4121489614
C8	-2.0738889036	11.0934275253	10.4617046740
C24	-1.6078086160	8.0382328509	6.1867920594
C14	-0.1100808316	4.4894424249	10.3915059913
C23	-2.4515650857	8.0088215669	7.3155189706
C21	0.0732640323	4.4278410864	11.7842273833
C20	0.9466102558	3.4644422234	12.2858408620
H20	1.1026837752	3.3873821692	13.3560318898
C11	-2.0410815262	8.7647856742	12.1481189981
H11	-2.0919603003	7.9138250956	12.8230075268
C30	-3.5335177411	8.8957455893	7.4555979304
C9	-2.9970518186	10.9653208801	11.5357469052
H9	-3.7211828896	11.7500653544	11.7358776408
C7	-1.8549588489	12.1491244092	9.5360933220
H7	-2.4738588672	13.0419434935	9.5623468156
C27	-2.9959114530	9.7903724635	5.2653520153
C15	0.6255055066	3.6716456640	9.5146170963
C26	-1.9065876107	8.9387731203	5.1662546905
H26	-1.2786025349	8.9761231683	4.2819226081
C18	1.6334263901	2.6039627701	11.4381550815
C5	0.0575393412	10.9397110023	8.5339295190
H5	0.8584015103	10.9516653034	7.7983363173
C10	-2.9627799430	9.8398818398	12.3362624338
H10	-3.6771855785	9.7649104995	13.1521915351
C29	-3.7916680100	9.7720653758	6.4006944604
H29	-4.6206879852	10.4668298306	6.4735731102
C22	-0.6535213705	5.3991961706	12.7011676547
H36	-0.5993609985	6.3825404913	12.2217857962
C37	1.4864910818	2.7200127294	10.0652645246
H38	2.0519144878	2.0670017769	9.4065415795
C39	-0.8321598880	12.0502897554	8.6100789518
H40	-0.6881376420	12.8724413239	7.9134951241
C41	-4.3941547983	8.9276399404	8.7122344217
H42	-3.7355981308	8.7148336134	9.5615287371
C43	2.4483238896	7.0555913371	9.9395780693
H44	1.7106301225	6.3803711845	9.4853371005
C45	-0.4192332050	7.0986477172	6.0340265077
H46	-0.0926240344	6.7916764676	7.0331610571
C47	3.3070168427	7.5604419711	8.7653601509
H48	4.1439028418	8.1774458203	9.1003135469
H49	2.6935531392	8.1587162499	8.0837159635
H50	3.7254917379	6.7181989586	8.2016465750
C51	0.5364066839	3.8125410643	8.0014308298
H52	-0.0880724509	4.6800381007	7.7759367732
B53	0.3475242597	8.4750851302	10.0623230899
C54	3.2628836797	6.1715363170	10.8854728705
H55	3.6773151045	5.3272622252	10.3250012388
H56	2.6250149168	5.7661879982	11.6740856928
H57	4.0990048992	6.7069941268	11.3456391521
C58	2.1724927502	8.4354440043	13.0060651008
H59	1.1675727976	8.0619232591	13.2211994468
H60	2.4198846407	9.2037369368	13.7468122190
H61	2.8823950266	7.6122488240	13.1269172251

C62	2.2037914579	9.0213970264	11.5880781004
H63	1.5082965670	9.8694093655	11.6018040975
C64	3.5731372195	9.6210594579	11.2514492984
H65	4.3720532262	8.8731724579	11.2652218091
H66	3.8303405979	10.3862461781	11.9909263090
H67	3.5548658695	10.0930152728	10.2652696847
H68	-2.4971563974	3.8741859592	9.3463531970
H69	-3.6460616487	5.6040427329	7.7244680620
H70	-3.2167089689	10.4843071721	4.4599560091
H71	2.3028333751	1.8565356299	11.8526513370
C72	-0.0006660057	5.5474308044	14.0769450024
H73	-0.4667931378	6.3782651607	14.6151513072
H74	-0.1304093240	4.6486438603	14.6902615655
H75	1.0689270441	5.7608024558	13.9954409660
C76	-2.1340340199	5.0182951221	12.8499224967
H77	-2.6471226181	5.7222677205	13.5135822386
H78	-2.6508641601	5.0257245702	11.8873040829
H79	-2.2271034823	4.0169845032	13.2848007107
C80	-5.0366691261	10.2987112255	8.9520224159
H81	-5.4377515441	10.3407658990	9.9684865008
H82	-4.3112113156	11.1070490090	8.8404127828
H83	-5.8688585207	10.4787725166	8.2614659723
C84	-5.5003355935	7.8605909156	8.6847760544
H85	-6.1426353416	7.9642499317	9.5654367208
H86	-6.1260557792	7.9813929609	7.7933291540
H87	-5.0988230989	6.8464429088	8.6885903298
C88	0.8003886147	7.7638862115	5.3882003955
H89	1.6678743799	7.1008986402	5.4687578748
H90	0.6456258671	7.9697185516	4.3237779096
H91	1.0424961642	8.7064955541	5.8881847940
C92	-0.8300917459	5.8262737936	5.2806551837
H93	0.0229938319	5.1486240235	5.1702693218
H94	-1.6226294399	5.2917378175	5.8128786045
H95	-1.1976083162	6.0757450052	4.2797837803
C96	-0.1342801295	2.5949082888	7.3548347660
H97	-0.2201849231	2.7359034449	6.2722953110
H98	0.4464969226	1.6832299807	7.5319483055
H99	-1.1390031094	2.4385321586	7.7573162160
C100	1.9155560863	4.0779670736	7.3836828869
H101	1.8204246150	4.2702999680	6.3097406077
H102	2.3918476619	4.9485303717	7.8427030306
H103	2.5877557552	3.2224459021	7.5062175121

Structure **10**

N3	0.0467465216	5.2816763559	8.9266863816
N2	-0.7957238096	6.9055106223	7.1480682646
N1	2.5636200913	8.3506172461	8.2587102109
C12	-0.1105519246	4.7340870714	7.6287072426
C2	-1.2102865231	7.8971266833	10.0398270860
C13	-0.5599234404	5.5881572521	6.6950602601
Si7	-0.1460312900	7.0310453141	8.7874835831
C3	-0.5722033372	9.1069309860	10.4685552771
C4	0.7450544741	9.4565075639	9.9688515064
C8	-1.2379698455	9.9312219748	11.4168851865
C24	-1.3669590719	8.9183576803	5.8550779768
C14	0.0088018678	4.4672625215	10.0963503824
C23	-1.7654163041	7.7322683978	6.5011716931
C21	-1.0318933744	3.5309991777	10.2959005513
C20	-1.0118170644	2.7327160915	11.4396852907
H20	-1.7998231654	1.9988543024	11.5850680885
C11	-2.4325173601	7.5406637520	10.5591719119
H11	-2.8982090067	6.6100604431	10.2481844788
C30	-3.1275056171	7.3576425671	6.5123483087
C9	-2.5110452215	9.5375901442	11.9045941882
H9	-3.0124431413	10.1782164360	12.6245515817
C7	-0.5993329167	11.1141573872	11.8658963131
H7	-1.1094993406	11.7496659826	12.5852165204
C27	-3.6478982843	9.2635055012	5.1094091761
C15	1.0148650702	4.6117968039	11.0786304652
C26	-2.3236244559	9.6668243617	5.1681310756
H26	-2.0189970254	10.5783280686	4.6606044335
C18	-0.0335792369	2.8764916276	12.4099274732
C5	1.3149510968	10.6091910604	10.4797296975
H5	2.3128449094	10.9089977379	10.1849133291
C10	-3.0938984420	8.3663154476	11.4934946681
H10	-4.0613518057	8.0698293625	11.8869092894
C29	-4.0421345561	8.1193633019	5.7873412234
H29	-5.0870709699	7.8230380539	5.7711137989
C22	-2.2092184603	3.3759138542	9.3430886951
H36	-2.1344474073	4.1477579764	8.5781046671
C37	0.9589801318	3.8252801369	12.2285670486
H38	1.7203972658	3.9520133316	12.9932042015
C39	0.6500893745	11.4379359552	11.4105439772
H40	1.1449154644	12.3356638029	11.7686940637
C41	-3.6330125378	6.1841682520	7.3354501331
H42	-2.8324927230	5.9100693173	8.0262327762
C43	2.9911864516	7.0858630678	7.6047865758
H44	2.3192831407	6.3265649285	8.0109155709
C45	0.0685579172	9.4096979774	5.8749611942
H46	0.6456345321	8.7120690672	6.4791722579
C47	2.7944815193	7.0161366860	6.0865054913
H48	3.4281938213	7.7102770152	5.5325728769
H49	1.7520431921	7.1983898032	5.8241055511
H50	3.0401844783	6.0021100342	5.7545850381
H51	-0.0498127020	2.2607717400	13.3038666015
C52	2.1612359101	5.6028896697	10.9495312439
H53	2.1109193040	6.0463460361	9.9549820357
H54	-4.3749095236	9.8482610169	4.5540619296
B55	1.3543529171	8.3674008858	8.9744922584
C56	4.4025735173	6.6488172536	8.0045499612
H57	4.5579630253	5.6120983921	7.6920981764
H58	4.5339094077	6.6970193491	9.0871309116
H59	5.1819166505	7.2532651887	7.5319580246
C60	4.4701214928	9.5714084777	9.3477172179
H61	4.0157468168	9.2737849236	10.2969964005

H62	4.8734559765	10.5833983717	9.4627290014
H63	5.3091332226	8.9020976995	9.1451044797
C64	3.4272906266	9.5489646195	8.2219379749
H65	2.7403358346	10.3790160925	8.3963800114
C66	4.0655015071	9.8514448927	6.8653854589
H67	4.8323762957	9.1269628472	6.5807395309
H68	4.5478035327	10.8323792756	6.9193689742
H69	3.3102822136	9.8909760913	6.0767286123
H70	0.1769282819	3.7054270316	7.4544001775
H71	-0.6979268534	5.3563156389	5.6467577411
C72	-2.1839291637	2.0213107623	8.6240396213
H73	-3.0119749753	1.9492390086	7.9111042600
H74	-1.2486004830	1.8821061929	8.0742323884
H75	-2.2779476674	1.1935106080	9.3357791056
C76	-3.5463177916	3.5776939736	10.0688594531
H77	-4.3745747183	3.5589138754	9.3524952071
H78	-3.7346893807	2.7908684547	10.8066760840
H79	-3.5701031365	4.5363471912	10.5957030645
C80	-4.8526571445	6.5726713334	8.1840662501
H81	-5.0826991943	5.7771641470	8.9002214521
H82	-4.6660259108	7.4941055838	8.7429617552
H83	-5.7457972122	6.7242130789	7.5691454379
C84	-3.9413369511	4.9573564940	6.4686812780
H85	-4.3069377074	4.1304774463	7.0874417071
H86	-4.7127580748	5.1894206270	5.7259020968
H87	-3.0481950438	4.6139303649	5.9411052987
C88	0.1876470273	10.7863131091	6.5414184312
H89	1.2374319929	11.0973160704	6.5875571089
H90	-0.3609969017	11.5550471506	5.9861647555
H91	-0.2019867079	10.7636333403	7.5635353448
C92	0.6725645250	9.4121475018	4.4652460526
H93	1.7303813359	9.6960156664	4.4941711238
H94	0.6017551827	8.4210224275	4.0065330680
H95	0.1556661347	10.1218621662	3.8104765806
C96	3.5270833380	4.9138039662	11.0636250389
H97	4.3346451046	5.6476891375	10.9741118618
H98	3.6498050101	4.4150748794	12.0301945053
H99	3.6570037327	4.1631155493	10.2784890545
C100	2.0323890583	6.7402898909	11.9725668700
H101	2.7947319187	7.5068440062	11.7943824721
H102	1.0518847677	7.2210968717	11.9149232643
H103	2.1620883174	6.3659778627	12.9940191629

Structure **10 - hypothetical**

N3	-0.1662095554	5.2192991431	9.4442706008
N2	-0.4259794491	7.2949810584	7.3698310223
N1	2.0223885138	8.3092025417	7.5973012076
C12	-0.3468057654	4.9456418121	8.0745841107
H12	-0.3702896656	3.8992074706	7.7925424124
C2	-0.3340879783	7.8373820977	11.0741822892
C13	-0.5036426982	5.9214515957	7.1481270728
H13	-0.7687026514	5.6154054423	6.1376302859
Si9	0.4061571226	6.7974095042	9.7573891189
C3	-0.4714840066	9.1136425929	10.4168865043
C4	-0.0602987131	9.2913388754	9.0400091991
C8	-1.0826971925	10.1744054145	11.1406747445
C24	-0.9745492023	8.5990632383	5.3485406431
C14	-0.3173972210	4.1696635404	10.4108816919
C23	-1.3643414471	8.0666700519	6.5933858650
C21	-1.5896086419	3.8350747477	10.8996368636
C20	-1.6756003800	2.8544096463	11.8888128909
H20	-2.6497772585	2.5879798127	12.2891386199
C11	-0.7513822705	7.6563633124	12.3733609097
H11	-0.6469562043	6.6857089080	12.8508247273
C30	-2.6639175767	8.2965318298	7.0838777370
C9	-1.4830643727	9.9519994780	12.4825626049
H9	-1.9363986871	10.7752006957	13.0289534601
C7	-1.3076659756	11.4130886773	10.4888677818
H7	-1.7751435619	12.2233176156	11.0406376154
C27	-3.1502784762	9.6408574120	5.1247336954
C15	0.8434721444	3.5126697686	10.8650972575
C26	-1.8807683494	9.3821953338	4.6334635034
H26	-1.5841189480	9.7905258301	3.6710359097
C18	-0.5417830602	2.2167609550	12.3696890644
C5	-0.3588014986	10.5053031129	8.4591562278
H5	-0.1299908235	10.6600794690	7.4082564513
C10	-1.3238652061	8.7295642092	13.0876527694
H10	-1.6463459456	8.5835826441	14.1137621110
C29	-3.5339323458	9.0917903020	6.3378652162
H29	-4.5352431646	9.2800404165	6.7153841523
C22	-2.8538568086	4.4954704595	10.3824769317
H22A	-2.5648337588	5.1931698516	9.5957834781
C39	0.7057222771	2.5409140915	11.8551078344
H40	1.5887317361	2.0321017072	12.2305011711
C41	-0.9708210179	11.5625198884	9.1705917616
H42	-1.1741649298	12.4988521835	8.6594794353
C43	-3.1819011277	7.6827214416	8.3705832522
H44	-2.3395836280	7.2109032077	8.8817876332
C45	2.7845225368	7.0800205870	7.3247289110
H46	2.0235218134	6.2940860133	7.2664892913
C47	0.3841463998	8.3163206536	4.7395908756
H48	0.9401735006	7.7270316384	5.4640125522
C49	3.5181162388	7.0162055560	5.9722794896
H50	4.4526022483	7.5806959681	5.9705931548
H51	2.8965894150	7.3934270968	5.1590666324
H52	3.7694839853	5.9734889673	5.7489262434
H53	-0.6303987447	1.4611155907	13.1441341469
C54	2.2261540746	3.8538398680	10.3294392577
H55	2.1013733518	4.4784283577	9.4404969426
H56	-3.8425941311	10.2573190206	4.5592431581
B57	0.6703856930	8.0629301823	8.2138713950
C58	3.7574664239	6.6563252183	8.4398251590
H59	4.0773151249	5.6181679399	8.2919403223
H60	3.2891300225	6.7305925504	9.4258934407
H61	4.6572970606	7.2791252493	8.4472943137

C62	3.1609731567	9.6498318087	9.4557404948
H63	2.3396990579	9.3909511351	10.1292581854
H64	3.4541670558	10.6818822584	9.6794958197
H65	4.0128763568	9.0067354684	9.6914614320
C66	2.7335100584	9.5392224633	7.9766837806
H67	2.0105228941	10.3443413292	7.8221552600
C68	3.9227810759	9.8906782846	7.0772885063
H69	4.7884537781	9.2464194023	7.2607158753
H70	4.2380225796	10.9182196543	7.2864691463
H71	3.6570912527	9.8239710283	6.0198364800
C72	-3.5564538872	5.2988470460	11.4837095313
H73	-4.4707598745	5.7619714782	11.0991903826
H74	-3.8386005503	4.6605946092	12.3283247851
H75	-2.9080995741	6.0957902721	11.8578545259
C76	-3.7912897421	3.4556791776	9.7547669487
H77	-4.6749033525	3.9410397338	9.3303400330
H78	-3.2856034391	2.9098264013	8.9528192682
H79	-4.1355641465	2.7256432289	10.4948566624
C80	-3.7748059224	8.7245361710	9.3263594696
H81	-3.9359077161	8.2801042928	10.3142717916
H82	-3.1065215826	9.5795136102	9.4413621131
H83	-4.7417839027	9.0949800426	8.9679002186
C84	-4.2145060686	6.5961799650	8.0403085864
H85	-4.6662371834	6.1992463197	8.9537298180
H86	-5.0216084434	7.0035822801	7.4225573339
H87	-3.7540742319	5.7680609614	7.4931995751
C88	1.1765079320	9.6055993194	4.5088153888
H89	2.1715570449	9.3776515099	4.1109372787
H90	0.6775800279	10.2676097801	3.7922831811
H91	1.3072298803	10.1467995435	5.4493728467
C92	0.2604081603	7.5013436512	3.4461155903
H93	1.2530714937	7.2337249365	3.0671262398
H94	-0.2990997442	6.5763727733	3.6142573590
H95	-0.2553911451	8.0645413765	2.6603686790
C96	3.0180773853	4.6642867089	11.3648944574
H97	4.0016669466	4.9403829655	10.9732357605
H98	2.4937938214	5.5882940966	11.6392734751
H99	3.1632206163	4.0870933013	12.2844004793
C100	3.0083990395	2.6118139688	9.8867185853
H101	3.9458336301	2.9137789385	9.4085474947
H102	3.2651722963	1.9636892636	10.7310236167
H103	2.4335867041	2.0204124365	9.1683700733

Structures **1** and **11**

N3	-1.2779144148	5.2292445473	8.8993527469
N2	-1.7941307543	7.1067835456	7.1587361223
N1	1.2449994485	10.2867120079	10.1825803761
C2	-0.0031793028	9.2571437120	12.4390628761
Ga5	-0.7081013731	7.0993749369	9.0377561088
C3	-1.3747684302	9.5451036975	12.2761728440
C4	-1.4232091565	10.2067335889	11.0292179878
C8	-2.4298947798	9.2347751967	13.1356340439
C14	-0.7331904160	4.2280682098	9.7467814562
C21	-1.3706802070	3.9015618285	10.9588472694
C20	-0.7766223125	2.9579972692	11.7984700963
H20	-1.2509044030	2.7075456259	12.7436435769
C11	0.3541707101	8.5847480060	13.5841108136
H11	1.3776001346	8.2970664163	13.8110805833
C9	-2.0202328262	8.5553947349	14.3140340119
H9	-2.7493092545	8.2597705168	15.0639904490
C7	-3.6990858198	9.6588594938	12.6559776957
H7	-4.6002323898	9.4735011658	13.2336457272
C15	0.4797346592	3.6054609192	9.3827504613
C18	0.4108939034	2.3323414590	11.4425150506
C5	-2.6683206645	10.6126879779	10.6054401843
H5	-2.8357150138	11.1525755247	9.6765282876
C10	-0.6817244203	8.2519912664	14.5052109971
H10	-0.4044629871	7.7215378139	15.4129522535
C22	-2.6624620125	4.5954122278	11.3550198921
H36	-3.0487433853	5.0846733274	10.4562245742
C37	1.0297294897	2.6545983785	10.2413824759
H38	1.9611576028	2.1624642583	9.9705297333
C39	-3.7871753629	10.3188893529	11.4413928809
H40	-4.7712744005	10.6356520141	11.1037050567
C43	2.5956996046	9.8707670440	10.6114634403
H44	2.5062866711	9.7670408495	11.6973078952
C47	3.0018471799	8.4910291038	10.0762140932
H48	3.2526881806	8.5131959362	9.0120086153
H49	2.1845579893	7.7785411919	10.2194484706
H50	3.8824483983	8.1286885068	10.6176825734
H51	0.8578008050	1.5952970859	12.1036732022
C52	1.1871267868	3.9594526910	8.0818210531
H53	0.6391932184	4.7949757472	7.6377724358
B55	0.1754467435	9.9697728843	11.0121210725
C56	3.6823419259	10.9180608410	10.3638512222
H57	4.6084544050	10.5965114898	10.8506064059
H58	3.3985538467	11.8893310012	10.7782993868
H59	3.9025677171	11.0435636866	9.2991327138
C60	1.2537860184	12.3920301809	8.8179043646
H61	0.8317029035	12.8939610679	9.6928362838
H62	0.8044566475	12.8318459906	7.9200161905
H63	2.3256437871	12.6011390402	8.7832405194
C64	0.9510646451	10.8897361376	8.8736151443
H65	-0.1347561971	10.7880135669	8.7800111435
C66	1.5572455339	10.1422742783	7.6838402421
H67	2.6466579115	10.2365864348	7.6440275249
H68	1.1596543089	10.5539966978	6.7494222283
H69	1.2950445399	9.0810145192	7.7288688242
C88	2.6225788209	4.4418729547	8.3257090522
H89	3.0952451441	4.7414578125	7.3830024345
H90	2.6340080268	5.3042909820	9.0009564424
H91	3.2452455405	3.6581580785	8.7708429872
C92	1.1369970959	2.7979595994	7.0805656309
H93	1.6111476731	3.0795264262	6.1317769418
H94	1.6643140531	1.9177275047	7.4661639053

H95	0.1011969892	2.5128874543	6.8730891835
C96	-3.7335317185	3.6139625644	11.8420642007
H97	-4.6761953784	4.1426709536	12.0192155778
H98	-3.9162749600	2.8314135833	11.0989474689
H99	-3.4499264396	3.1284080514	12.7827245415
C100	-2.3867125739	5.6867937177	12.3983931625
H101	-3.3035859834	6.2310954558	12.6499982883
H102	-1.9829357403	5.2561634952	13.3223013532
H103	-1.6568353995	6.4165400674	12.0313552193
C104	-2.0526364889	4.8334781675	7.8287717286
C105	-2.3369399905	5.8367395977	6.8715553384
C106	-3.1243197897	5.5567033809	5.7585222984
C107	-3.6130301225	4.2669156347	5.5513055104
C108	-3.3202835694	3.2690068459	6.4750652058
C109	-2.5551041838	3.5444514792	7.6059437102
H112	-3.3469442299	6.3522263141	5.0503450494
H113	-4.2228945927	4.0510147600	4.6795531337
H114	-3.6971677657	2.2617112383	6.3219865392
H115	-2.3336013800	2.7624397938	8.3252929652
C81	-1.3422749483	7.8471727259	6.1495823290
N82	-0.5898126325	7.4668774678	5.0768167642
C83	-0.3106996060	8.5832345916	4.3045549847
C84	-0.8766171994	9.6489685720	4.9070134469
N85	-1.5113884974	9.1918568911	6.0517394163
H87	0.2699986528	8.5220081228	3.3990289921
H88	-0.8826034680	10.6857172610	4.6184592676
C89	-2.3215068083	9.9684594854	7.0071552778
H92	-1.9820339223	9.6637546961	8.0021562409
C90	0.0839599052	6.1642383490	4.9421584404
H96	-0.3446765101	5.5271277270	5.7160890108
C93	1.5790057805	6.3279951881	5.2110964460
H100	2.0652341349	5.3482776011	5.1896567284
H104	2.0576527202	6.9626542449	4.4571226160
H105	1.7491191576	6.7699898101	6.1978486441
C97	-0.2249390192	5.5403761751	3.5865851298
H106	-1.3035226309	5.4175901958	3.4628104644
H107	0.1639231128	6.1481288161	2.7617760480
H108	0.2411490125	4.5535601509	3.5252383915
C101	-3.8005143260	9.6169931790	6.8613611811
H109	-4.3789977881	10.1488155666	7.6227268266
H110	-4.1709197927	9.9109311167	5.8733064175
H111	-3.9550188673	8.5449624622	7.0004567833
C110	-2.0659808715	11.4596782156	6.8282086078
H116	-1.0004054859	11.7007962435	6.8752700370
H117	-2.4709742773	11.8266642595	5.8789193377
H118	-2.5669521115	12.0079213790	7.6296377419

Structure **12 - int 1**

N3	-1.6506980805	4.8538314514	8.6784685266
N2	-1.7626754399	6.7020490405	6.8766424952
N1	0.8130992983	9.3294526224	9.2747686456
C2	0.0327230744	7.8740417637	11.4664893479
Ga5	-0.7926927118	6.5663310240	8.6595524287
C3	-1.2881715013	8.3164401712	11.6760256087
C4	-1.6641402367	8.8416575179	10.4210788713
C8	-2.0473611794	8.2416602472	12.8441286270
C14	-1.2975159204	3.8140021203	9.5830199842
C21	-1.8590604722	3.7821393739	10.8755383364
C20	-1.4548710528	2.7639213111	11.7403051202
H20	-1.8645514229	2.7172318883	12.7434898440
C11	0.6647986041	7.2995893209	12.5438642481
H11	1.6788895209	6.9096029266	12.5006989351
C9	-1.3519254538	7.6687878677	13.9435606973
H9	-1.8353927891	7.5673089969	14.9115390318
C7	-3.3701206220	8.7490058592	12.7088257455
H7	-4.0567175511	8.7393117199	13.5517619885
C15	-0.3414139851	2.8584755443	9.1799412080
C18	-0.5261187597	1.8082238890	11.3491219456
C5	-2.9495621620	9.3279869660	10.3305948626
H5	-3.3605649410	9.7736055674	9.4253668411
C10	-0.0532819143	7.2211665467	13.7752104707
H10	0.4504445051	6.7794844093	14.6322790113
C22	-2.8707601418	4.8383678330	11.3072835178
H36	-2.4755178234	5.8150807695	11.0015652994
C37	0.0297801600	1.8606834348	10.0793198775
H38	0.7722249885	1.1220619296	9.7877292438
C39	-3.7810699693	9.2635124681	11.4920784409
H40	-4.7958968075	9.6474896709	11.4138878863
C43	2.2406964958	9.0964393485	9.4789551189
H44	2.2904070513	8.5850559408	10.4459145720
C47	2.8864932509	8.1349017274	8.4613575059
H48	3.0686169423	8.6105288332	7.4923547803
H49	2.2409896208	7.2628348611	8.3066478070
H50	3.8513830882	7.7758310979	8.8361971155
H51	-0.2267970172	1.0275649743	12.0426213129
C52	0.3291896835	2.9345617284	7.8131436922
H53	-0.2362811990	3.6389799433	7.2001282791
B55	-0.1212250406	8.4352902580	9.9348677879
C56	3.0720118715	10.3745134341	9.6294552733
H57	4.0933652354	10.1189353104	9.9317942773
H58	2.6395885494	11.0271063288	10.3928999525
H59	3.1425004135	10.9377204105	8.6926525231
C60	0.1893809635	11.6826687559	8.7285646828
H61	-0.3193442028	11.7188887186	9.6953267314
H62	-0.3775094625	12.2932699176	8.0141701662
H63	1.1769187208	12.1384550206	8.8403834235
C64	0.2811258230	10.2243645178	8.2547110349
H65	-0.7543279992	9.8903645070	8.1121480583
C66	0.9456138799	10.1276401819	6.8762781650
H67	1.9767864518	10.4926828562	6.8849909155
H68	0.3938090974	10.7361467989	6.1510760374
H69	0.9510539740	9.0941893686	6.5149705944
C88	1.7549707669	3.4886442029	7.9420195214
H89	2.2233261236	3.5924510202	6.9553688883
H90	1.7508374310	4.4727146921	8.4247979306
H91	2.3850515554	2.8259553844	8.5455477525
C92	0.3066745113	1.5957343132	7.0676931441
H93	0.7252559024	1.7138719371	6.0618034080
H94	0.8963887068	0.8271733109	7.5791880349

H95	-0.7189984177	1.2287459727	6.9666222711
C96	-4.2229774893	4.6464771255	10.6044515029
H97	-4.9142057375	5.4439919595	10.8983931841
H98	-4.1247104231	4.6651258693	9.5179340557
H99	-4.6684544812	3.6863157633	10.8904862190
C100	-3.0815606640	4.9018020262	12.8204789003
H101	-3.7119987376	5.7627842462	13.0621014241
H102	-3.5871220852	4.0042474723	13.1970984635
H103	-2.1356233989	5.0257727755	13.3538079107
C104	-2.4020954985	4.5256821424	7.5644733993
C105	-2.4590764471	5.5168308592	6.5570988626
C106	-3.1547866644	5.2878425521	5.3730984271
C107	-3.8017645125	4.0679499122	5.1677435437
C108	-3.7548039644	3.0908495632	6.1551020339
C109	-3.0654076690	3.3124388722	7.3454556334
H112	-3.1933954164	6.0613147974	4.6081257741
H113	-4.3427903010	3.8926630390	4.2431172471
H114	-4.2606010044	2.1419242238	6.0032594682
H115	-3.0249098968	2.5452906365	8.1122124790
C81	-1.4427205678	7.5177096303	5.8579793054
N82	-0.5553118458	7.2713525197	4.8662464010
C83	-0.5321537732	8.3494398537	4.0052414247
C84	-1.4038605662	9.2679647825	4.4855519893
N85	-1.9552412715	8.7467642421	5.6387095652
H87	0.1094448327	8.3816934478	3.1402459931
H88	-1.6559284730	10.2475464872	4.1164486496
C89	-3.0108743109	9.3270160597	6.4904414427
H92	-2.7727404720	8.9801862442	7.4988925130
C90	0.2953341053	6.0709736008	4.8029422444
H96	0.0006810078	5.4762704801	5.6710302878
C93	1.7616941148	6.4680732171	4.9478948739
H100	2.3828685998	5.5688626311	4.9724760788
H104	2.0954690134	7.0861784853	4.1072808585
H105	1.9268313148	7.0218098804	5.8761847311
C97	-0.0005035885	5.2762765881	3.5362707453
H106	-1.0548007069	4.9929862502	3.5022714259
H107	0.2482807395	5.8495947151	2.6363605871
H108	0.6001531932	4.3630906830	3.5305073309
C101	-4.3728044416	8.7685775912	6.0867953555
H109	-5.1447188473	9.1731578644	6.7473489944
H110	-4.6177646923	9.0498635675	5.0566945409
H111	-4.3866281315	7.6791819485	6.1711640608
C110	-2.9589944697	10.8478658483	6.4490017962
H116	-1.9537901527	11.2229477141	6.6571892254
H117	-3.2944986380	11.2393639248	5.4822876565
H118	-3.6301902261	11.2451263990	7.2142784238

Structure **12 - ts 2**

N3	-1.0646076222	5.1414122464	9.8779735785
N2	-2.3408666474	6.9439045806	8.5407867607
N1	1.9331254224	8.4795799523	10.1850188192
C2	-0.8938901170	9.0558179735	11.0765939348
Ga5	-0.6534296806	6.9362702544	9.5099406229
C3	-1.0631102114	10.1132376470	10.1513278981
C4	-0.1219360732	9.8991356772	9.1233659959
C8	-1.9155365319	11.2203251135	10.2710428558
N9	-2.1702199809	8.0958525086	6.4372724411
C14	-0.1480812090	4.2247866781	10.4602924819
C23	-2.6863862722	7.9213864054	7.6728664393
C21	-0.1102590416	4.0861395890	11.8629501624
C20	0.8236307203	3.2126002236	12.4204798176
H20	0.8734697199	3.0919555864	13.4976384292
C11	-1.6726267403	9.1330711234	12.2144896789
H11	-1.6331066851	8.3760851521	12.9978011490
N17	-3.5341656933	8.9446536386	7.9056879821
C9	-2.6997214973	11.2505183159	11.4573135147
H9	-3.3880660385	12.0718149010	11.6418605741
C7	-1.8378038617	12.1620253582	9.2069655427
H7	-2.4457380092	13.0640331950	9.2265465459
C15	0.7568853762	3.5263154189	9.6376494535
C26	-2.6781388004	9.2562074941	5.8977189665
C18	1.7081374347	2.5038316786	11.6162467813
C5	-0.0658761173	10.8342177131	8.1130913450
H5	0.6497058136	10.7688083180	7.2956908653
C10	-2.5681884079	10.2337122734	12.3832102680
H10	-3.1764389787	10.2732747596	13.2850227578
C29	-3.5264183976	9.7835833268	6.8119069033
C22	-1.0685650144	4.8901631836	12.7311856919
H36	-1.1554096754	5.8834759387	12.2755712788
C37	1.6775276554	2.6677554447	10.2388365425
H38	2.3878342902	2.1260878712	9.6188552474
C39	-0.9469100351	11.9532799156	8.1714086461
H40	-0.8934849592	12.6951399929	7.3771401513
C41	-4.3178427377	9.1049866848	9.1559620518
H42	-3.5876958389	9.0643488973	9.9682133134
C43	2.4385590058	7.1926043059	10.6667005835
H44	1.9240802256	6.4461842357	10.0454359581
C45	-1.1540704914	7.2184160039	5.8335995943
H46	-1.1146531006	6.3433696836	6.4869484930
C47	3.9319979823	6.9493474893	10.4371176280
H48	4.5613913663	7.5617132686	11.0908397828
H49	4.2068408090	7.1549302283	9.3983966302
H50	4.1595781626	5.8997213444	10.6519977257
C51	0.7766427916	3.7170629394	8.1270028529
H52	-0.0671897056	4.3577574351	7.8596750488
B53	0.5319021313	8.5738900602	9.8047106513
C54	2.0470246321	6.8473187780	12.1155533707
H55	2.1898268196	5.7765832094	12.2952086224
H56	0.9926815943	7.0881092227	12.2899922491
H57	2.6407198685	7.3985929766	12.8500024575
C58	2.9448456951	9.9319852261	12.0028584802
H59	2.0599855515	9.7497967438	12.6192990358
H60	3.2795934026	10.9604352651	12.1782476545
H61	3.7476161144	9.2666192351	12.3377343273
C62	2.6102908400	9.7412516148	10.5182055171
H63	1.8803446166	10.5205078370	10.2799634000
C64	3.8259715063	10.0374374347	9.6261376190
H65	4.6947776609	9.4222701640	9.8750125165
H66	4.1238739580	11.0869338275	9.7338995027

H67	3.5689299424	9.8583916536	8.5777598141
H71	2.4317747784	1.8312703703	12.0675808883
C72	-0.5759182426	5.0933682011	14.1660932791
H73	-1.2308999445	5.8008524140	14.6849200661
H74	-0.5910859255	4.1591493255	14.7395132722
H75	0.4419748489	5.4944336168	14.1900585223
C76	-2.4733142499	4.2682647819	12.7247945102
H77	-3.1638839589	4.8754973543	13.3206732672
H78	-2.8740171359	4.1932655023	11.7110281108
H79	-2.4472018420	3.2611211337	13.1567701708
C80	-5.0179061667	10.4571609926	9.1702692137
H81	-5.4669530783	10.5985341512	10.1556887552
H82	-4.3175890149	11.2780325919	9.0072835126
H83	-5.8192885682	10.5002847029	8.4235844922
C84	-5.3224937886	7.9660377400	9.3064392254
H85	-5.9015000609	8.1250177408	10.2201767079
H86	-6.0170884309	7.9497557110	8.4593975066
H87	-4.8319818983	6.9952740472	9.3795852318
C88	0.2051645110	7.9134688146	5.8366998698
H89	0.9664100281	7.2334462271	5.4444416513
H90	0.1894572860	8.8101906062	5.2080018020
H91	0.4894467184	8.2053215547	6.8513075011
C92	-1.6100952137	6.7713831042	4.4487514983
H93	-0.8945515707	6.0503506973	4.0456898460
H94	-2.5911193137	6.2919196117	4.5007636663
H95	-1.6632224891	7.6134201669	3.7501560471
C96	0.5803078430	2.3925887120	7.3799833453
H97	0.5401942020	2.5662924000	6.2990384626
H98	1.4009190773	1.6925775072	7.5724169421
H99	-0.3562313046	1.9146488082	7.6814072703
C100	2.0572251290	4.4339440688	7.6757861946
H101	2.0518135683	4.5848872922	6.5897911057
H102	2.1534950877	5.4143521720	8.1541358537
H103	2.9501196643	3.8511066067	7.9270406006
C104	-2.1523030168	4.6637886587	9.1584201450
C105	-2.8468593204	5.6340030771	8.3928967137
C106	-3.9046978586	5.2619921783	7.5743347883
C107	-4.3062713223	3.9247790416	7.5138042898
C108	-3.6472353222	2.9719149736	8.2785861664
C109	-2.5764910843	3.3353938651	9.0973133414
H112	-4.4200372888	6.0175463074	6.9846106324
H113	-5.1335720535	3.6409348952	6.8710723286
H114	-3.9582449285	1.9325242611	8.2395667370
H115	-2.0502035955	2.5884158414	9.6837348870
H106	-4.1005665249	10.6922171369	6.7775338461
H107	-2.3882323334	9.6175473796	4.9253413360

Structure **12**

N3	-0.0847608289	5.0604917366	9.0877213683
N2	-0.4950933880	6.7692188223	7.1179833001
N1	2.5567406807	8.7172704312	8.6666427356
C2	-1.5410070191	8.1726506889	9.7966508512
Ga5	-0.0706791432	6.9893281958	9.1074405486
C3	-0.9933905924	9.4646949459	10.1160253468
C4	0.4356013415	9.7248367195	9.9927419755
C8	-1.8621623899	10.4921026112	10.5960531407
N9	-1.3687055730	8.7559017485	6.1540676401
C14	0.0236967822	4.1887950987	10.2036552274
C23	-1.4756072721	7.4587243054	6.5331756516
C21	-1.1507417768	3.8014551962	10.8836102549
C20	-1.0462125215	2.9524436532	11.9843391738
H20	-1.9441148145	2.6456636459	12.5133518532
C11	-2.8968887523	7.9800627686	9.9519092183
H11	-3.3229366851	6.9970233819	9.7656288278
N17	-2.7513559012	7.0710768788	6.2557345226
C9	-3.2574770999	10.2438755830	10.6867540572
H9	-3.9071847696	11.0421473458	11.0366563112
C7	-1.3172878493	11.7339689989	11.0148725247
H7	-1.9888480971	12.5077899128	11.3798051747
C15	1.2791799685	3.7105023410	10.6256476365
C18	0.1903171762	2.4847613865	12.4126506993
C5	0.8962252470	10.9341006131	10.4898842393
H5	1.9598373799	11.1464345667	10.5067114088
C10	-3.7675778170	9.0117114372	10.3741082522
H10	-4.8332113171	8.8186939899	10.4703496722
C22	-2.5081978742	4.2580628515	10.3746971235
H36	-2.3314128927	5.1758876265	9.8096082600
C37	1.3397171401	2.8641887259	11.7339172089
H38	2.3058223854	2.4941272088	12.0691912000
C39	0.0364774918	11.9337755729	10.9975730940
H40	0.4587242370	12.8670471334	11.3596335774
C41	-3.3917471170	5.8516620965	6.7844323611
H42	-2.7114593133	5.4913487889	7.5612456326
C43	3.2180930404	7.4935582873	8.1459225978
H44	2.7344354828	6.6879452565	8.6989118833
C45	-0.1111530153	9.5147997918	6.1493999309
H46	0.5550659303	8.9749143160	6.8244153775
C47	2.9456661539	7.2058780012	6.6627005573
H48	3.4860009066	7.8756687306	5.9887774894
H49	1.8752569149	7.2724674198	6.4538343168
H50	3.2566506724	6.1809840155	6.4324287193
H51	0.2562540811	1.8233134627	13.2715251359
C52	2.5585053128	4.1026042903	9.9047942179
H53	2.2721698457	4.6767705464	9.0197638737
B55	1.3224516146	8.5648447453	9.3404825630
C56	4.7094476236	7.3895493295	8.4701552340
H57	5.0521794642	6.3802815697	8.2192499699
H58	4.8941154598	7.5550613040	9.5348462252
H59	5.3232340570	8.0894564743	7.8956342320
C60	4.2561691939	10.3859000999	9.4830983859
H61	3.9368595439	10.0886014226	10.4861116909
H62	4.4392047553	11.4666396401	9.4855008655
H63	5.2069684940	9.8903150189	9.2720117552
C64	3.1752325150	10.0395905793	8.4473146853
H65	2.3582876311	10.7492264747	8.5899194781
C66	3.6800048792	10.2950440566	7.0245667483
H67	4.5623522817	9.7037626084	6.7672292711
H68	3.9571060560	11.3505983941	6.9340553773
H69	2.9007982070	10.0855296218	6.2877842642

C72	-3.0832284996	3.2252793132	9.3937157825
H73	-4.0531019679	3.5555559885	9.0009614715
H74	-2.4053292010	3.0658512758	8.5494732854
H75	-3.2357188350	2.2612034469	9.8914483117
C76	-3.5047828428	4.5888533200	11.4874728077
H77	-4.4112785644	5.0319930964	11.0595360061
H78	-3.8138650744	3.6978513743	12.0455496899
H79	-3.0804384320	5.3075376109	12.1947291091
C80	-4.7355641211	6.2072406928	7.4228325461
H81	-5.0783313788	5.3644678537	8.0290896498
H82	-4.6524965045	7.0858813516	8.0656237075
H83	-5.5016847610	6.3977958137	6.6632700955
C84	-3.5501080488	4.7825637517	5.7083797391
H85	-4.0174522737	3.8962777939	6.1470769718
H86	-4.1956115082	5.1442160935	4.8998112728
H87	-2.5848123336	4.4902733377	5.2934141857
C88	-0.3234582618	10.9207639073	6.6974857462
H89	0.6485305288	11.4111097383	6.7984477925
H90	-0.9352508078	11.5338246866	6.0260024556
H91	-0.7934231485	10.8894994353	7.6835449704
C92	0.4781306108	9.4954606989	4.7395183487
H93	1.4210817385	10.0480808270	4.7112467984
H94	0.6735315463	8.4679224994	4.4189983888
H95	-0.2077891647	9.9609090346	4.0230196572
C96	3.3446916610	2.8817786959	9.4103462230
H97	4.2231766253	3.2029060729	8.8398197457
H98	3.6986056228	2.2601684102	10.2405460419
H99	2.7278568348	2.2590824837	8.7560734086
C100	3.4263117135	5.0020764370	10.7942784851
H101	4.3560919859	5.2728078413	10.2832717818
H102	2.8960728587	5.9256283075	11.0513016285
H103	3.6931539511	4.4943252041	11.7278812477
C104	-0.0418790279	4.5230866466	7.8162955139
C105	-0.2553483413	5.4263023510	6.7442831128
C106	-0.1427567180	5.0070193619	5.4276175775
C107	0.1098976647	3.6655357055	5.1292490610
C108	0.2607179926	2.7604180990	6.1716536736
C109	0.1905918757	3.1773479631	7.5008898621
H112	-0.2665692354	5.7378396261	4.6308651335
H113	0.1898718702	3.3422660314	4.0961510591
H114	0.4483464132	1.7120602128	5.9566201367
H115	0.3150895739	2.4599290058	8.3060411654
C110	-2.5712664756	9.1759018984	5.6130780887
C111	-3.4232338270	8.1321680031	5.6675748060
H106	-2.7128633353	10.1759315814	5.2388371343
H107	-4.4522791987	8.0569779679	5.3595431376

Structure **12 - hypothetical**

N3	-0.4362860059	5.2028875640	9.6633754599
N2	-0.2099021524	7.2050994433	7.5330950363
N1	2.2632513996	8.1580802130	7.5795690946
C2	-1.0457309648	8.3020920911	10.6311607523
Ga5	0.2875309335	6.9565702095	10.0047181543
C3	-0.7680823048	9.4708724145	9.8437796032
C4	0.2516751909	9.4335117597	8.8104775602
C8	-1.4646438195	10.6835162694	10.1214673049
N9	-1.0596705188	8.8444097842	5.9230164074
C14	-0.3834398591	4.2278301478	10.7043110606
C23	-1.1988027143	7.9012805003	6.8900023413
C21	-1.5173821003	4.0171408045	11.5154230521
C20	-1.4406886688	3.1032530732	12.5660356214
H20	-2.3130014094	2.9308219187	13.1899984663
C11	-2.0186261935	8.3672380853	11.6040895683
H11	-2.2191037757	7.5026086319	12.2322659139
N17	-2.5254160878	7.7466961358	7.0978037817
C9	-2.4896983973	10.6813355877	11.1045467682
H9	-3.0324803259	11.6043924688	11.2955651250
C7	-1.0981158108	11.8739828951	9.4404493522
H7	-1.6344583236	12.7947305625	9.6553358744
C15	0.8193495597	3.5378620768	10.9818743728
C26	-2.3123426659	9.2753672581	5.5365004743
C18	-0.2695732393	2.4058640529	12.8278089365
C5	0.6049910700	10.6431675490	8.2495299594
H5	1.4092312401	10.6606011099	7.5190819453
C10	-2.7657236674	9.5481223671	11.8262983652
H10	-3.5407918778	9.5632253847	12.5884138620
C29	-3.2212813289	8.5834658708	6.2554586649
C22	-2.8261947351	4.7239848764	11.2116419616
H22A	-2.5764720819	5.6506897737	10.6876158798
C39	0.8496248521	2.6294922155	12.0395714599
H40	1.7720322590	2.0945905981	12.2534300507
C41	-0.0536716670	11.8571339130	8.5562535234
H42	0.2624067752	12.7749476558	8.0655001335
C43	-3.1632148509	6.7938975654	8.0232439540
H44	-2.3642408595	6.4145635690	8.6609523111
C45	2.6048452177	7.1674898129	6.5555500267
H46	1.6679135448	7.0087405021	6.0170267511
C47	0.1904653206	9.3062901037	5.2812380636
H48	0.9889152828	9.1079253603	6.0011522695
C49	3.6027860389	7.6704544943	5.5069528014
H50	4.6270782357	7.6928417074	5.8913383469
H51	3.3459355438	8.6782509443	5.1636837571
H52	3.5967691611	6.9971251057	4.6421369633
H53	-0.2281028389	1.6963296520	13.6493189893
C54	2.0836277482	3.7745624076	10.1713765810
H55	1.8458110202	4.5049866662	9.3988489507
B57	0.9464362479	8.0326069290	8.2853673308
C58	3.0185909065	5.7654201338	7.0429682523
H59	2.8635855506	5.0323404546	6.2431976948
H60	2.4010513433	5.4633015393	7.8886576766
H61	4.0671682076	5.7155823336	7.3484258832
C62	4.2224657894	7.7552049250	9.1420818418
H63	3.6299187832	6.9584162915	9.6000485942
H64	4.7855397855	8.2618365219	9.9332690618
H65	4.9513762257	7.3057179171	8.4596437681
C66	3.3082593536	8.7573174202	8.4232351581
H67	2.7525145899	9.2744198798	9.2122045317
C68	4.1467236666	9.8407125911	7.7254479828
H69	4.9116062218	9.4211249607	7.0663687466

H70	4.6622496396	10.4511668807	8.4752321256
H71	3.5139227075	10.5023567001	7.1260721342
C72	-3.6285772499	5.0984810630	12.4615159450
H73	-4.4430560972	5.7809209101	12.1947599872
H74	-4.0830128870	4.2216188496	12.9363021672
H75	-3.0006851525	5.5942424757	13.2080068605
C76	-3.6695609792	3.8510321658	10.2727491445
H77	-4.6183695136	4.3422098557	10.0243120113
H78	-3.1287568154	3.6383417197	9.3455063012
H79	-3.9054803614	2.8940612929	10.7524726085
C80	-4.1773801656	7.5183889362	8.9012030413
H81	-4.5142586820	6.8354953269	9.6863051427
H82	-3.7249199828	8.3884918143	9.3810355256
H83	-5.0604534058	7.8325230023	8.3334600132
C84	-3.7753035367	5.6533913619	7.2116209979
H85	-4.2915752226	4.9600312615	7.8764830841
H86	-4.5081098642	6.0408546311	6.4946193484
H87	-3.0057769163	5.1015764365	6.6657855096
C88	0.1180341069	10.8086465306	5.0107662617
H89	1.1263814421	11.1758806327	4.8010569841
H90	-0.5004550685	11.0350581920	4.1346418417
H91	-0.2711323481	11.3509791406	5.8741589404
C92	0.4460762708	8.5384654046	3.9844248354
H93	1.3407633681	8.9400976335	3.5002068729
H94	0.6203509367	7.4759670735	4.1610231252
H95	-0.3957208795	8.6466102147	3.2916079891
C96	3.1993417542	4.3822587441	11.0313332134
H97	4.0736110248	4.6128765870	10.4127548857
H98	2.8688166029	5.3120723519	11.5072929461
H99	3.5180505356	3.6948408958	11.8233302184
C100	2.5637294872	2.5042377684	9.4596937601
H101	3.4314486150	2.7302482664	8.8307184347
H102	2.8608230160	1.7289132376	10.1754884159
H103	1.7796469367	2.0947577705	8.8168682344
C104	-0.4029947188	4.8139989882	8.3424922292
C105	-0.2895998761	5.7715351353	7.2912989379
C106	-0.3502306668	5.3403120524	5.9668064609
C107	-0.4966259431	4.0047064004	5.6184445181
C108	-0.5866528960	3.0627105882	6.6390616443
C109	-0.5385480099	3.4615300039	7.9635807763
H112	-0.2981737102	6.0893094673	5.1835826397
H113	-0.5387071013	3.7111063781	4.5748086092
H114	-0.7020740727	2.0080046812	6.4053373593
H115	-0.6294897647	2.7218533163	8.7516431379
H106	-4.2971358561	8.6281225005	6.2531250221
H107	-2.4478899317	10.0438943732	4.7949332568

5. References

[1] A. Hergel, H. Pritzkow, W. Siebert, *Angew. Chem. Int. Ed.* **1994**, *33,* 1247-1248.

[2] L. Hintermann, *Beilstein J. Org. Chem.* **2007**, *3*, no. 22.

[3] a) P. Zark, A. Schäfer, A. Mitra, D. Haase, W. Saak, R. West, T, Müller, *J. Organomet. Chem.* **2010**, *695*, 398-408.

b) L. Kong, J. Zhang, H. Song, C. Cui, *Dalton Trans.* **2009**, 5444-5446.

[4] L. Denker, B. Trzaskowski, R. Frank, *Chem. Commun.* **2021**, *57*, 2816-2819.

[5] J.-D. Chaia, M. Head-Gordon, *Phys. Chem. Chem. Phys.* **2008**, *10*, 6615-6620.

[6] A. D. Bochevarov, E. Harder, T. F. Hughes, J. R. Greenwood, D. A. Braden, D. M. Philipp, D. Rinaldo, M. D. Halls, J. Zhang, R. A. Friesner, *Int. J. Quant. Chem.* **2013**, *113*, 2110-2142.

[7] S. Miertuš, E. Scrocco, J.Tomasi, *Chem. Phys.* **1981**, *55*, 117-129.
